# Supplementary material for: Polygenic associations with clinical and neuropathological trait heterogeneity across TDP-43 proteinopathies
Source: PLoS One. 2025 Dec 30;20(12):e0338398. doi: 10.1371/journal.pone.0338398 (PMC12752993; doi:10.1371/journal.pone.0338398)
Supplement: S1 File — SNPs that remain after pruning are listed for each cluster. (PDF) [file pone.0338398.s008.pdf]

Chromosome	Position_GRCh38	rsID	Cluster
chr1	1869595	rs4648592	1
chr1	4321193	rs2171985	1
chr1	4337468	rs684965	1
chr1	4560863	rs10915560	1
chr1	5238999	rs551536	1
chr1	6508327	rs4908554	1
chr1	7232712	rs4908608	1
chr1	7698700	rs12756299	1
chr1	13646755	rs12561812	1
chr1	17086006	rs2076616	1
chr1	18799290	rs12042960	1
chr1	20078672	rs521179	1
chr1	22170210	rs909813	1
chr1	22770325	rs7516175	1
chr1	22814207	rs2869511	1
chr1	23212517	rs10917360	1
chr1	29815360	rs2166884	1
chr1	33436150	rs7544329	1
chr1	34149975	rs4653395	1
chr1	48610454	rs320019	1
chr1	50644495	rs12567589	1
chr1	52590925	rs11591099	1
chr1	55539504	rs1740113	1
chr1	57175802	rs7530049	1
chr1	63121375	rs17124643	1
chr1	76373234	rs11162133	1
chr1	85996226	rs11161714	1
chr1	86022231	rs11161721	1
chr1	86116996	rs1507292	1
chr1	87774383	rs2436974	1
chr1	92080868	rs12739985	1
chr1	93748070	rs7552104	1
chr1	93769311	rs2747038	1
chr1	96649493	rs6673531	1
chr1	96973935	rs3002273	1
chr1	101048152	rs11166538	1
chr1	102451358	rs2376037	1
chr1	102765472	rs618072	1
chr1	103087379	rs1415363	1
chr1	106249336	rs7543568	1

chr1	110861171	rs12026259	1
chr1	114601683	rs12730318	1
chr1	114685275	rs2268699	1
chr1	150986360	rs267733	1
chr1	152286602	rs11204971	1
chr1	153668351	rs7536700	1
chr1	158766655	rs1864346	1
chr1	160407087	rs6663715	1
chr1	161234748	rs2501873	1
chr1	161242663	rs11584174	1
chr1	161751917	rs1063178	1
chr1	162044842	rs2880058	1
chr1	163397064	rs10799916	1
chr1	164680043	rs6426879	1
chr1	164847489	rs6426881	1
chr1	167446554	rs1554669	1
chr1	180169665	rs6703180	1
chr1	180200270	rs7513063	1
chr1	180572227	rs12030731	1
chr1	180815742	rs2944259	1
chr1	181033343	rs3806284	1
chr1	181070392	rs17302632	1
chr1	186179900	rs1407434	1
chr1	197999256	rs1125953	1
chr1	201750207	rs239989	1
chr1	204938656	rs2802822	1
chr1	204986288	rs11240326	1
chr1	204991111	rs3820336	1
chr1	207062609	rs2075863	1
chr1	209418897	rs11580645	1
chr1	211181496	rs10494934	1
chr1	213282877	rs1187802	1
chr1	216540972	rs17042779	1
chr1	223663031	rs1109223	1
chr1	224064136	rs7531519	1
chr1	224076680	rs10916260	1
chr1	224095362	rs1573185	1
chr1	224213894	rs6689187	1
chr1	228939221	rs6701969	1
chr1	233127221	rs6691107	1
chr1	234144826	rs12073596	1

chr1	234314248	rs561159	1
chr1	237032806	rs12032218	1
chr1	237291723	rs7538075	1
chr1	237643519	rs11583646	1
chr1	239898335	rs10802816	1
chr2	1618368	rs9752413	1
chr2	3337168	rs11885038	1
chr2	5690467	rs3922853	1
chr2	7826846	rs7601867	1
chr2	7986660	rs2111074	1
chr2	18312107	rs5005196	1
chr2	20543956	rs11096672	1
chr2	24267544	rs4268898	1
chr2	29731798	rs2631956	1
chr2	31004165	rs12991678	1
chr2	33818359	rs13417671	1
chr2	33822143	rs7565016	1
chr2	35118154	rs2119082	1
chr2	40581909	rs10198059	1
chr2	42235790	rs13416119	1
chr2	42388356	rs7560797	1
chr2	44675221	rs12612153	1
chr2	47004933	rs11899526	1
chr2	48352639	rs7606104	1
chr2	51785140	rs1516199	1
chr2	51793222	rs17864614	1
chr2	58758906	rs10176091	1
chr2	59251853	rs5008666	1
chr2	59884087	rs12991146	1
chr2	65168812	rs11903118	1
chr2	75959356	rs2197078	1
chr2	75999249	rs6708139	1
chr2	76005200	rs4853189	1
chr2	78968955	rs17693274	1
chr2	80343381	rs1373264	1
chr2	88460447	rs11693918	1
chr2	103919258	rs1441104	1
chr2	104873939	rs2060783	1
chr2	104983381	rs4851727	1
chr2	106583062	rs968914	1
chr2	111032214	rs13012948	1

chr2	112820380	rs7596461	1
chr2	112948594	rs3923566	1
chr2	114864622	rs13007523	1
chr2	119520595	rs4383344	1
chr2	127762973	rs6731093	1
chr2	129063127	rs10928866	1
chr2	135901925	rs12478902	1
chr2	137641336	rs881639	1
chr2	138690053	rs7594562	1
chr2	145586665	rs10186882	1
chr2	151475231	rs16830067	1
chr2	163169687	rs1489636	1
chr2	163975310	rs165231	1
chr2	164010159	rs357309	1
chr2	167646759	rs1147155	1
chr2	168793861	rs3845727	1
chr2	170572859	rs11693682	1
chr2	171847131	rs925881	1
chr2	177283476	rs2364731	1
chr2	179568810	rs999000	1
chr2	179585255	rs16866809	1
chr2	179643054	rs10930879	1
chr2	181264071	rs1899037	1
chr2	183606069	rs6710374	1
chr2	200779509	rs2348129	1
chr2	201999764	rs4675246	1
chr2	204192945	rs13401647	1
chr2	207807187	rs10932209	1
chr2	216663886	rs9341218	1
chr2	217077992	rs2372960	1
chr2	218741495	rs1554622	1
chr2	222014814	rs17315907	1
chr2	222231249	rs1549773	1
chr2	224151769	rs12619528	1
chr2	224346689	rs6745530	1
chr2	227706159	rs7585761	1
chr2	228291114	rs10498227	1
chr2	228318301	rs16824642	1
chr2	230920311	rs16827668	1
chr2	230925382	rs3111780	1
chr2	238590694	rs1510510	1

chr2	239892262	rs10933613	1
chr2	241130647	rs3213680	1
chr2	241454726	rs764081	1
chr3	376902	rs3773379	1
chr3	873706	rs1464701	1
chr3	899015	rs2313182	1
chr3	1074220	rs1857699	1
chr3	2293378	rs11915941	1
chr3	3013563	rs17024684	1
chr3	7798131	rs721257	1
chr3	10458120	rs12492242	1
chr3	13841863	rs9863149	1
chr3	14200648	rs2305843	1
chr3	14263841	rs13072044	1
chr3	14393563	rs721377	1
chr3	23157962	rs9818043	1
chr3	29669222	rs7634507	1
chr3	29749272	rs6792183	1
chr3	39481512	rs1768208	1
chr3	44570558	rs9873604	1
chr3	44645808	rs1402752	1
chr3	47998793	rs11711953	1
chr3	51058304	rs4927960	1
chr3	55275572	rs7649746	1
chr3	57985796	rs1866164	1
chr3	58669165	rs1444186	1
chr3	58868540	rs17360960	1
chr3	60103845	rs10510827	1
chr3	60424454	rs241692	1
chr3	65653197	rs1499497	1
chr3	66662140	rs9839845	1
chr3	66671258	rs6782747	1
chr3	70856430	rs2687195	1
chr3	71311557	rs7633896	1
chr3	71316220	rs17655524	1
chr3	72122732	rs17009412	1
chr3	72217160	rs4677113	1
chr3	75196623	rs9828201	1
chr3	97883893	rs10935130	1
chr3	98747191	rs828598	1
chr3	100874422	rs9863850	1

chr3	104041332	rs4420906	1
chr3	111604007	rs7431849	1
chr3	114298411	rs2693051	1
chr3	130020067	rs6439205	1
chr3	134655756	rs11719419	1
chr3	137488261	rs4234226	1
chr3	146126586	rs4681298	1
chr3	146143518	rs1707469	1
chr3	153790428	rs924825	1
chr3	157068755	rs11719102	1
chr3	171223294	rs7374644	1
chr3	171962723	rs7619106	1
chr3	175451941	rs6801770	1
chr3	186115970	rs7635103	1
chr3	187500711	rs2600990	1
chr3	191227844	rs6801419	1
chr3	196990442	rs569330	1
chr4	703059	rs12646225	1
chr4	1318606	rs17742599	1
chr4	1526039	rs7666926	1
chr4	2309634	rs4974674	1
chr4	2342124	rs2071678	1
chr4	2829656	rs231399	1
chr4	4937696	rs11946368	1
chr4	6876175	rs6828754	1
chr4	7432290	rs10033032	1
chr4	8116834	rs10938692	1
chr4	8727745	rs10755200	1
chr4	14007236	rs9291619	1
chr4	20482633	rs17612037	1
chr4	25016113	rs10488953	1
chr4	25940970	rs1456435	1
chr4	28024330	rs4140905	1
chr4	29724472	rs12641755	1
chr4	31865585	rs6827011	1
chr4	37769347	rs1030350	1
chr4	38425077	rs7679805	1
chr4	41130074	rs2256007	1
chr4	41207844	rs4590083	1
chr4	41705076	rs12650313	1
chr4	47509764	rs10000432	1

chr4	47531821	rs7674353	1
chr4	54675242	rs6820303	1
chr4	60268481	rs6853571	1
chr4	62329832	rs1478528	1
chr4	69441766	rs12650761	1
chr4	72278465	rs1369095	1
chr4	77776632	rs9307306	1
chr4	79477554	rs10027646	1
chr4	79677642	rs13123790	1
chr4	80078917	rs7693145	1
chr4	83239126	rs7658518	1
chr4	85818652	rs12498568	1
chr4	87622676	rs2627730	1
chr4	95414339	rs17439081	1
chr4	98546473	rs6532746	1
chr4	99321852	rs1159918	1
chr4	99831995	rs2162386	1
chr4	101786634	rs17199964	1
chr4	101866266	rs4698977	1
chr4	107678390	rs727137	1
chr4	108742458	rs17527308	1
chr4	109627238	rs10017480	1
chr4	109733484	rs2346841	1
chr4	112494246	rs7699752	1
chr4	114908763	rs7675371	1
chr4	117636163	rs17862059	1
chr4	120666114	rs1580743	1
chr4	121321648	rs12650310	1
chr4	123556545	rs4076851	1
chr4	123827822	rs979755	1
chr4	129942948	rs10012483	1
chr4	133166608	rs17020496	1
chr4	134242606	rs2421243	1
chr4	134500552	rs10006423	1
chr4	136930704	rs1373895	1
chr4	137358691	rs10519423	1
chr4	140891491	rs1863307	1
chr4	146076224	rs1035803	1
chr4	152714217	rs11930234	1
chr4	154051916	rs6535980	1
chr4	158698883	rs7679753	1

chr4	158756560	rs7666007	1
chr4	167041297	rs10517919	1
chr4	168675498	rs13137200	1
chr4	169393217	rs7672874	1
chr4	169619890	rs3797041	1
chr4	169991127	rs7661337	1
chr4	174304301	rs4695918	1
chr4	186410505	rs11945366	1
chr5	3503064	rs4866578	1
chr5	5938358	rs1174902	1
chr5	7983578	rs11748296	1
chr5	8439243	rs4461605	1
chr5	10219056	rs10866471	1
chr5	16837753	rs17651266	1
chr5	19048511	rs4333315	1
chr5	22826012	rs1661975	1
chr5	22849375	rs4701204	1
chr5	29249437	rs7717683	1
chr5	31951855	rs16901697	1
chr5	34826684	rs10461948	1
chr5	38036178	rs10473079	1
chr5	38135616	rs3812048	1
chr5	38735182	rs17456364	1
chr5	41617154	rs17276531	1
chr5	53537360	rs10078892	1
chr5	61677470	rs391101	1
chr5	68958125	rs11951431	1
chr5	76509280	rs6453227	1
chr5	76891483	rs7722773	1
chr5	77295873	rs12514694	1
chr5	81949312	rs11740142	1
chr5	82666969	rs2055438	1
chr5	82882038	rs4383715	1
chr5	88812757	rs700588	1
chr5	88861735	rs4518438	1
chr5	96291166	rs13158163	1
chr5	96773540	rs28096	1
chr5	108512271	rs9326729	1
chr5	108696765	rs2174973	1
chr5	109108853	rs4957798	1
chr5	113344640	rs1116547	1

chr5	113451146	rs26990	1
chr5	113591058	rs2303718	1
chr5	113718283	rs985411	1
chr5	113718683	rs6885608	1
chr5	113939678	rs4705607	1
chr5	113986227	rs12658199	1
chr5	116607498	rs607930	1
chr5	118459959	rs10046044	1
chr5	119079098	rs11742151	1
chr5	127320808	rs152120	1
chr5	128837115	rs2158175	1
chr5	129427451	rs12656498	1
chr5	133190859	rs7715671	1
chr5	133447495	rs25869	1
chr5	134594838	rs17167632	1
chr5	136413179	rs1499772	1
chr5	142283199	rs4912841	1
chr5	143176167	rs6894946	1
chr5	144672484	rs17387218	1
chr5	151022235	rs3828599	1
chr5	151052827	rs871269	1
chr5	151052885	rs17111695	1
chr5	151066407	rs3792789	1
chr5	151094113	rs999556	1
chr5	151165010	rs7731328	1
chr5	151181776	rs1061837	1
chr5	153418119	rs13361457	1
chr5	157914383	rs17608701	1
chr5	160656487	rs2217637	1
chr5	167475105	rs7378751	1
chr5	167596996	rs1903110	1
chr5	169973390	rs13165483	1
chr5	171141736	rs4868069	1
chr5	172920900	rs7717884	1
chr5	173184645	rs255313	1
chr5	173229188	rs3095872	1
chr5	173827349	rs1506726	1
chr5	174322572	rs6861937	1
chr5	178330763	rs2913788	1
chr5	178902845	rs953741	1
chr5	178964901	rs12719860	1

chr5	179517406	rs936817	1
chr5	179523760	rs1344158	1
chr5	180307312	rs3763131	1
chr6	1529552	rs2569865	1
chr6	2954323	rs2295767	1
chr6	2970334	rs7743193	1
chr6	5387280	rs2432755	1
chr6	9116165	rs6911727	1
chr6	9137048	rs10484328	1
chr6	9301633	rs11967465	1
chr6	10887018	rs9366663	1
chr6	11500384	rs9394119	1
chr6	11612742	rs515145	1
chr6	12302337	rs11755836	1
chr6	13597890	rs7759452	1
chr6	16805989	rs11752823	1
chr6	23035104	rs2498259	1
chr6	23089271	rs12523660	1
chr6	25151193	rs9356963	1
chr6	27332801	rs1156457	1
chr6	27427985	rs6926142	1
chr6	31113122	rs2233965	1
chr6	34514490	rs11753634	1
chr6	34515238	rs4713808	1
chr6	40885000	rs4711652	1
chr6	42252734	rs12194245	1
chr6	46407589	rs2153615	1
chr6	46850245	rs6458525	1
chr6	50661441	rs280317	1
chr6	52262783	rs1413920	1
chr6	52326459	rs6458829	1
chr6	53098273	rs2876	1
chr6	66268753	rs7763510	1
chr6	66950965	rs6930795	1
chr6	70956443	rs1320315	1
chr6	72405882	rs11754392	1
chr6	73970322	rs3003187	1
chr6	73975267	rs9293946	1
chr6	81725071	rs12206095	1
chr6	83116819	rs7764458	1
chr6	86234881	rs7765374	1

chr6	90902361	rs9451525	1
chr6	93321933	rs164302	1
chr6	103176933	rs494028	1
chr6	106497026	rs9480677	1
chr6	108714193	rs522611	1
chr6	108760394	rs566575	1
chr6	118278665	rs1889468	1
chr6	120608745	rs1203087	1
chr6	120635397	rs9401311	1
chr6	121452853	rs2389541	1
chr6	122657277	rs2316962	1
chr6	127725568	rs564461	1
chr6	135800421	rs7767870	1
chr6	140315494	rs1157002	1
chr6	148448972	rs7763869	1
chr6	148516555	rs17715583	1
chr6	151254115	rs7764018	1
chr6	151261497	rs9383562	1
chr6	152658699	rs9383636	1
chr6	153122024	rs6557273	1
chr6	155428270	rs231951	1
chr6	155539784	rs11156029	1
chr6	156068014	rs4870428	1
chr6	156265619	rs9478696	1
chr6	156360035	rs9384456	1
chr6	157035957	rs2207227	1
chr6	161902965	rs6455783	1
chr6	162651750	rs1893540	1
chr6	162847872	rs1333962	1
chr6	165384456	rs11754977	1
chr6	166947236	rs1079145	1
chr6	167283460	rs3010558	1
chr6	169040761	rs7765333	1
chr6	169217631	rs8089	1
chr6	170483679	rs12189977	1
chr7	4406242	rs1447402	1
chr7	5455046	rs6964614	1
chr7	5686391	rs4724712	1
chr7	8984751	rs6962574	1
chr7	8988208	rs6979515	1
chr7	8992665	rs10255740	1

chr7	9010163	rs13241564	1
chr7	9097190	rs2286111	1
chr7	13838346	rs10230244	1
chr7	13963480	rs2282869	1
chr7	19476869	rs2192491	1
chr7	21339069	rs6461554	1
chr7	22272446	rs17146488	1
chr7	22276689	rs1636899	1
chr7	22361019	rs7785685	1
chr7	24784176	rs17211952	1
chr7	25260370	rs2107124	1
chr7	26519873	rs10240247	1
chr7	26852046	rs7804356	1
chr7	30745210	rs4645485	1
chr7	36689719	rs4077337	1
chr7	36691170	rs4072404	1
chr7	37438260	rs17171046	1
chr7	37901650	rs1530820	1
chr7	38144185	rs1357647	1
chr7	42348598	rs1012325	1
chr7	42388980	rs12530988	1
chr7	43307079	rs12532870	1
chr7	43660747	rs10281917	1
chr7	46573011	rs10261793	1
chr7	53716938	rs1012052	1
chr7	55056275	rs11773818	1
chr7	71300110	rs1317429	1
chr7	78729198	rs4370470	1
chr7	88719132	rs1966296	1
chr7	95481979	rs43065	1
chr7	101049095	rs6968731	1
chr7	103537500	rs362710	1
chr7	103552269	rs362791	1
chr7	106508773	rs17153258	1
chr7	107836101	rs4730271	1
chr7	122677224	rs4140960	1
chr7	125053989	rs1404897	1
chr7	125265006	rs11771079	1
chr7	133620975	rs6956399	1
chr7	137986794	rs11770714	1
chr7	142727107	rs6959895	1

chr7	148303600	rs1110038	1
chr7	152678103	rs2040639	1
chr7	153048725	rs4725479	1
chr7	153127146	rs940650	1
chr7	155435665	rs11768588	1
chr7	155437846	rs6948976	1
chr8	699190	rs12156406	1
chr8	1733567	rs2972180	1
chr8	2560586	rs6996532	1
chr8	3011224	rs11786445	1
chr8	3785733	rs1383951	1
chr8	5514091	rs1011000	1
chr8	6771406	rs17078076	1
chr8	8351675	rs4840932	1
chr8	8384155	rs4840341	1
chr8	9030178	rs2953807	1
chr8	9103507	rs1809006	1
chr8	10367649	rs7015455	1
chr8	10403043	rs1962073	1
chr8	10477522	rs7842777	1
chr8	11554314	rs17744726	1
chr8	11722293	rs10112596	1
chr8	11778508	rs4840584	1
chr8	11789425	rs904015	1
chr8	11908511	rs6993684	1
chr8	17598351	rs2588139	1
chr8	19652775	rs17128793	1
chr8	22318020	rs4242431	1
chr8	22585707	rs11994391	1
chr8	23392434	rs7824768	1
chr8	23401864	rs17696480	1
chr8	26282307	rs4545109	1
chr8	39692348	rs7465283	1
chr8	40947632	rs7827529	1
chr8	51447983	rs7846408	1
chr8	53396524	rs10958356	1
chr8	54559955	rs7827437	1
chr8	54892163	rs13249752	1
chr8	58116301	rs9792139	1
chr8	58281277	rs4602872	1
chr8	58436494	rs6993992	1

chr8	58547499	rs12375332	1
chr8	72824531	rs1107217	1
chr8	73318674	rs16938613	1
chr8	75235719	rs16939046	1
chr8	92345976	rs1125730	1
chr8	105280710	rs16872638	1
chr8	106830067	rs2930492	1
chr8	128988278	rs7829061	1
chr8	129447166	rs1368136	1
chr8	132282725	rs1457780	1
chr8	133049467	rs2252917	1
chr8	135363415	rs7813136	1
chr8	135634305	rs4909494	1
chr8	136274480	rs13260793	1
chr8	138773995	rs11781300	1
chr8	142040310	rs4917300	1
chr8	143358686	rs2931710	1
chr8	143944186	rs11786896	1
chr9	2140197	rs7035986	1
chr9	6672097	rs1658957	1
chr9	8180576	rs10815776	1
chr9	10292422	rs10959029	1
chr9	10295912	rs616040	1
chr9	11022581	rs10959466	1
chr9	12234969	rs946452	1
chr9	13106599	rs3264	1
chr9	15288047	rs10810369	1
chr9	17616195	rs3808755	1
chr9	18973920	rs6475294	1
chr9	19126567	rs3824369	1
chr9	21925856	rs7852128	1
chr9	22911501	rs10965517	1
chr9	23569872	rs17698074	1
chr9	27233970	rs1590255	1
chr9	27468463	rs10511816	1
chr9	27478054	rs1822723	1
chr9	27502988	rs1977661	1
chr9	27519175	rs11792285	1
chr9	27543283	rs3849942	1
chr9	27559735	rs1565948	1
chr9	27572257	rs2282241	1

chr9	27586164	rs2453556	1
chr9	27596680	rs10812621	1
chr9	27609516	rs810174	1
chr9	28298085	rs10968468	1
chr9	34017108	rs1785506	1
chr9	34023574	rs1785502	1
chr9	34085861	rs7036030	1
chr9	34124862	rs2275003	1
chr9	34983977	rs4879847	1
chr9	36782016	rs2381583	1
chr9	68603133	rs1888722	1
chr9	68856996	rs6560397	1
chr9	68982398	rs4745451	1
chr9	69005698	rs10869686	1
chr9	74833606	rs11144108	1
chr9	88188557	rs17053864	1
chr9	90110024	rs7851675	1
chr9	98171545	rs4743179	1
chr9	98366576	rs3780429	1
chr9	102187693	rs10820085	1
chr9	106556480	rs1387596	1
chr9	106848175	rs4333673	1
chr9	108098233	rs10979206	1
chr9	112197858	rs10981305	1
chr9	112634926	rs11789059	1
chr9	114133316	rs10982083	1
chr9	128016235	rs7026544	1
chr9	129904796	rs6478924	1
chr9	129944885	rs7870884	1
chr9	130014745	rs10760653	1
chr9	130149826	rs7866942	1
chr9	134005665	rs419218	1
chr9	136027362	rs7388909	1
chr10	89913	rs7906287	1
chr10	2713735	rs945142	1
chr10	5654519	rs3750640	1
chr10	6262594	rs4750195	1
chr10	6480544	rs3793729	1
chr10	7655541	rs11255267	1
chr10	12755759	rs4132050	1
chr10	13832453	rs10906506	1

chr10	14670444	rs1459019	1
chr10	15354752	rs7083529	1
chr10	16792661	rs3824633	1
chr10	16903194	rs7099855	1
chr10	27434104	rs7342136	1
chr10	27521938	rs2642274	1
chr10	28432348	rs11007106	1
chr10	29787425	rs10826694	1
chr10	31700680	rs7901343	1
chr10	33410745	rs2785273	1
chr10	37411269	rs11011172	1
chr10	48790524	rs726786	1
chr10	49593296	rs1917804	1
chr10	52214509	rs12256137	1
chr10	59620687	rs2126772	1
chr10	69643146	rs10509319	1
chr10	73799109	rs2271271	1
chr10	75058179	rs1993005	1
chr10	77406587	rs670898	1
chr10	78566097	rs164226	1
chr10	83990267	rs10887195	1
chr10	92760912	rs10509646	1
chr10	93448022	rs809812	1
chr10	100219661	rs11595324	1
chr10	100641711	rs2495712	1
chr10	105962048	rs517866	1
chr10	106634856	rs10491052	1
chr10	106778012	rs12256169	1
chr10	107081261	rs17209374	1
chr10	110673089	rs10509925	1
chr10	111410490	rs9421093	1
chr10	111994786	rs1421050	1
chr10	112342122	rs11195905	1
chr10	118637083	rs877721	1
chr10	127507271	rs10830109	1
chr10	128448909	rs9804335	1
chr10	128449863	rs7100024	1
chr10	129562960	rs511770	1
chr10	129796586	rs1887181	1
chr10	131126683	rs1106524	1
chr10	131150577	rs7081792	1

chr10	131944854	rs9419387	1
chr10	131950560	rs4880246	1
chr10	132124775	rs7083603	1
chr10	132141327	rs10870265	1
chr10	132834775	rs12356978	1
chr11	5904202	rs11039518	1
chr11	8240854	rs3849994	1
chr11	9565931	rs7937639	1
chr11	14581152	rs17567703	1
chr11	21041143	rs11025886	1
chr11	22318896	rs7935452	1
chr11	23147258	rs11027007	1
chr11	34485666	rs1980719	1
chr11	35167698	rs353626	1
chr11	36944654	rs7109945	1
chr11	41109279	rs578718	1
chr11	43998165	rs10838212	1
chr11	45106751	rs3740787	1
chr11	57610852	rs2511989	1
chr11	58287727	rs12362065	1
chr11	59861832	rs557564	1
chr11	73806214	rs11235911	1
chr11	73837225	rs1792165	1
chr11	75143624	rs10899075	1
chr11	78114274	rs665278	1
chr11	78453938	rs4945276	1
chr11	79473855	rs7102569	1
chr11	82354196	rs7930387	1
chr11	82524933	rs2226620	1
chr11	88169611	rs302648	1
chr11	88377297	rs10765490	1
chr11	93510356	rs2605618	1
chr11	95341096	rs514284	1
chr11	99527840	rs2515376	1
chr11	102733876	rs2155053	1
chr11	103859255	rs12574570	1
chr11	107494465	rs4754226	1
chr11	107558697	rs1950031	1
chr11	113956604	rs17116178	1
chr11	117690550	rs10892162	1
chr11	122770548	rs11218802	1

chr11	123112220	rs4936777	1
chr11	123149523	rs12578013	1
chr11	126498537	rs11823106	1
chr11	127211696	rs4529888	1
chr11	133726227	rs10894740	1
chr11	133816572	rs2851128	1
chr12	2600714	rs215986	1
chr12	6386109	rs2364480	1
chr12	8055648	rs11057065	1
chr12	22851463	rs2467458	1
chr12	25681937	rs10771232	1
chr12	28084361	rs231210	1
chr12	29658538	rs16934692	1
chr12	39803007	rs4254134	1
chr12	39814235	rs10784079	1
chr12	40580596	rs12422396	1
chr12	46657293	rs10492250	1
chr12	50695504	rs2139930	1
chr12	50787916	rs10783388	1
chr12	51112545	rs4768967	1
chr12	56280982	rs773652	1
chr12	57454856	rs3809114	1
chr12	57584957	rs775251	1
chr12	57618450	rs11613457	1
chr12	57725064	rs4760169	1
chr12	57893419	rs17119981	1
chr12	58176018	rs17120420	1
chr12	63051312	rs10506458	1
chr12	64091580	rs789744	1
chr12	64092505	rs7960270	1
chr12	64213702	rs812590	1
chr12	64316939	rs12308116	1
chr12	64336726	rs10784404	1
chr12	64338514	rs11609829	1
chr12	64453031	rs7316037	1
chr12	64470203	rs11837890	1
chr12	64521280	rs1317532	1
chr12	64802197	rs7132617	1
chr12	65330944	rs10878260	1
chr12	68402668	rs7307444	1
chr12	71559910	rs7973299	1

chr12	71640351	rs1149009	1
chr12	72346482	rs4309196	1
chr12	77921555	rs2731424	1
chr12	89378126	rs704061	1
chr12	89386220	rs7305095	1
chr12	99997310	rs7306416	1
chr12	108332900	rs10735429	1
chr12	108467757	rs2111949	1
chr12	109438323	rs10850141	1
chr12	111446804	rs3184504	1
chr12	111536476	rs616668	1
chr12	116838412	rs10047539	1
chr12	122043384	rs12827036	1
chr12	124660848	rs838875	1
chr12	126998884	rs11058903	1
chr12	130229408	rs549257	1
chr13	21152899	rs729105	1
chr13	23417765	rs7320968	1
chr13	25676590	rs17082637	1
chr13	28318848	rs9319425	1
chr13	30561355	rs9508801	1
chr13	33227831	rs17078802	1
chr13	37929941	rs4943550	1
chr13	39268126	rs1413056	1
chr13	40181504	rs10507482	1
chr13	44234038	rs9533799	1
chr13	45524859	rs3014966	1
chr13	48074014	rs9567990	1
chr13	48557633	rs4942767	1
chr13	51064099	rs6561611	1
chr13	53017291	rs4884317	1
chr13	66860029	rs9564355	1
chr13	66913281	rs1323922	1
chr13	70416742	rs12583232	1
chr13	71699240	rs7993403	1
chr13	73182499	rs9600085	1
chr13	73448776	rs7327345	1
chr13	83498434	rs9575317	1
chr13	85955140	rs7997693	1
chr13	87319081	rs7999126	1
chr13	88252095	rs4278614	1

chr13	88380811	rs978165	1
chr13	89522272	rs2221406	1
chr13	92717616	rs1933192	1
chr13	98447084	rs2281767	1
chr13	99240059	rs2296911	1
chr13	102292510	rs17505423	1
chr13	102673692	rs660596	1
chr13	102700673	rs12427471	1
chr13	105251989	rs3015363	1
chr13	107575531	rs9301233	1
chr13	110292448	rs2391823	1
chr13	113570181	rs4907646	1
chr13	114275850	rs9562187	1
chr14	20656875	rs8003288	1
chr14	30116428	rs190123	1
chr14	30630532	rs229150	1
chr14	30669172	rs12894695	1
chr14	30766309	rs179534	1
chr14	30770756	rs179525	1
chr14	31503083	rs2378931	1
chr14	31829453	rs12886280	1
chr14	32259027	rs6571501	1
chr14	32858311	rs990721	1
chr14	33269492	rs1500719	1
chr14	34091538	rs4982138	1
chr14	34331617	rs1958578	1
chr14	44172432	rs1040691	1
chr14	47826113	rs1956314	1
chr14	47860435	rs451505	1
chr14	48217286	rs4319673	1
chr14	49943829	rs941605	1
chr14	52417362	rs809100	1
chr14	53554017	rs10145210	1
chr14	53584358	rs7152946	1
chr14	55077758	rs749053	1
chr14	55966754	rs1188147	1
chr14	59198052	rs1252917	1
chr14	67499905	rs10873202	1
chr14	72002874	rs2239268	1
chr14	76519067	rs1642851	1
chr14	76838946	rs6574333	1

chr14	79854741	rs17764956	1
chr14	82217289	rs1457979	1
chr14	88963991	rs1885185	1
chr14	92037924	rs2235978	1
chr14	92133833	rs17807992	1
chr14	93232657	rs740791	1
chr14	96681891	rs10484208	1
chr14	96865281	rs10135704	1
chr14	97004831	rs8017230	1
chr14	99619113	rs2144800	1
chr14	100148698	rs878078	1
chr14	103528624	rs1467561	1
chr15	25708051	rs17555920	1
chr15	27629529	rs3098551	1
chr15	32959526	rs6494794	1
chr15	33007031	rs12441885	1
chr15	33722317	rs11635093	1
chr15	40033659	rs8042947	1
chr15	51602114	rs12372948	1
chr15	60392977	rs11631777	1
chr15	60960980	rs12903220	1
chr15	60971178	rs17204698	1
chr15	60977248	rs8031801	1
chr15	60980538	rs1589703	1
chr15	63586733	rs7183892	1
chr15	70348536	rs6494855	1
chr15	79029175	rs10851915	1
chr15	81011791	rs11072972	1
chr15	82161045	rs8034586	1
chr15	82176114	rs1501372	1
chr15	82809417	rs8042254	1
chr15	82896041	rs7496832	1
chr15	82942343	rs7170046	1
chr15	83049857	rs12907032	1
chr15	83227856	rs7162082	1
chr15	85838928	rs7180790	1
chr15	91312351	rs8026639	1
chr15	92590195	rs285718	1
chr15	93463669	rs16948067	1
chr15	96658186	rs4335743	1
chr15	97480552	rs8038622	1

chr15	98898207	rs1464433	1
chr16	667523	rs3752493	1
chr16	674084	rs1045763	1
chr16	4880099	rs1876359	1
chr16	4885635	rs2075639	1
chr16	5359850	rs7198653	1
chr16	6412230	rs9926622	1
chr16	7392248	rs4786149	1
chr16	8565207	rs4580141	1
chr16	9777611	rs2267779	1
chr16	10066931	rs1448259	1
chr16	11269835	rs415595	1
chr16	12583902	rs7197841	1
chr16	13571085	rs2226994	1
chr16	19449520	rs6497373	1
chr16	50592284	rs7204135	1
chr16	50711288	rs2066843	1
chr16	60061475	rs36553	1
chr16	76982383	rs10431977	1
chr16	77677207	rs7193406	1
chr16	77734943	rs308927	1
chr16	77750112	rs9927857	1
chr16	77809313	rs2914446	1
chr16	79530084	rs9936252	1
chr16	80883984	rs9935116	1
chr16	81148012	rs4889247	1
chr16	81282928	rs11865869	1
chr16	84424708	rs247804	1
chr16	84751597	rs8058755	1
chr16	84979651	rs9935170	1
chr16	85193213	rs11646864	1
chr16	85337082	rs4783169	1
chr17	5478547	rs3865350	1
chr17	5492063	rs8081387	1
chr17	8473883	rs11650137	1
chr17	9220577	rs9897341	1
chr17	11059042	rs11078879	1
chr17	11184970	rs9891347	1
chr17	13452712	rs8080163	1
chr17	14303045	rs719470	1
chr17	27524665	rs2945378	1

chr17	28269102	rs9909055	1
chr17	28396803	rs739439	1
chr17	31679811	rs7210009	1
chr17	31729919	rs9909665	1
chr17	31756668	rs7212433	1
chr17	33285735	rs1497366	1
chr17	36025629	rs854658	1
chr17	36507271	rs8081787	1
chr17	36549567	rs9906189	1
chr17	37863709	rs10512474	1
chr17	41968753	rs8078650	1
chr17	42166716	rs9912576	1
chr17	42398593	rs4796649	1
chr17	43891933	rs1642592	1
chr17	48869635	rs12449856	1
chr17	53033072	rs792768	1
chr17	53192146	rs17626856	1
chr17	54721227	rs758647	1
chr17	55381043	rs8066468	1
chr17	57034731	rs8078110	1
chr17	57376248	rs1982270	1
chr17	57632675	rs3826301	1
chr17	58097128	rs8071026	1
chr17	67379776	rs11652752	1
chr17	75898723	rs7370	1
chr17	76916871	rs12603183	1
chr17	77012989	rs6501930	1
chr17	78368412	rs8074003	1
chr17	78396063	rs17561950	1
chr17	78399553	rs2292641	1
chr17	78500101	rs2028733	1
chr17	83073892	rs8072895	1
chr18	3087945	rs948299	1
chr18	6600606	rs11659717	1
chr18	8355050	rs7226445	1
chr18	12516769	rs11663391	1
chr18	22499276	rs494470	1
chr18	23732088	rs2337186	1
chr18	25434737	rs12971136	1
chr18	25459550	rs4800633	1
chr18	27067723	rs10502485	1

chr18	30276169	rs2960051	1
chr18	32683529	rs1403755	1
chr18	34076385	rs4239384	1
chr18	37334783	rs3865390	1
chr18	37390664	rs488797	1
chr18	38551867	rs1365468	1
chr18	44018694	rs4500824	1
chr18	44100804	rs1607100	1
chr18	46588774	rs1078174	1
chr18	50276088	rs140687	1
chr18	52429089	rs1145242	1
chr18	56187234	rs4570960	1
chr18	56238709	rs1229585	1
chr18	58370256	rs2075404	1
chr18	69534681	rs1442669	1
chr18	74147203	rs3813119	1
chr18	74190040	rs7240367	1
chr18	75528719	rs11665332	1
chr18	75573361	rs952058	1
chr18	77005659	rs509620	1
chr18	78874943	rs936501	1
chr19	6833766	rs186295	1
chr19	7693453	rs12971845	1
chr19	14566500	rs3752221	1
chr19	16624977	rs3786603	1
chr19	20025895	rs8106847	1
chr19	45351661	rs13181	1
chr19	45408744	rs735482	1
chr19	49374939	rs2288481	1
chr19	51529101	rs8182477	1
chr19	52118391	rs8111640	1
chr20	402232	rs6037542	1
chr20	671453	rs753490	1
chr20	21043741	rs6047216	1
chr20	34598676	rs2378199	1
chr20	34737712	rs1018503	1
chr20	36338061	rs6024358	1
chr20	36372640	rs2425222	1
chr20	38408937	rs1739646	1
chr20	41393111	rs4810316	1
chr20	44391463	rs3092370	1

chr20	49938133	rs2038127	1
chr20	49975221	rs6020157	1
chr20	50195590	rs2904254	1
chr20	56861662	rs6069911	1
chr20	58631557	rs6100145	1
chr20	59392851	rs1877751	1
chr20	61693013	rs12479878	1
chr20	63899393	rs4809244	1
chr20	63922145	rs6090020	1
chr21	16257417	rs2823702	1
chr21	16375756	rs370428	1
chr21	16376665	rs435082	1
chr21	19696842	rs1850587	1
chr21	19860120	rs2407489	1
chr21	21613860	rs2739356	1
chr21	23110971	rs2408666	1
chr21	26054932	rs2830028	1
chr21	26996145	rs2830607	1
chr21	29806309	rs2256853	1
chr21	31064491	rs2833249	1
chr21	32830972	rs6517112	1
chr21	34991865	rs11088302	1
chr21	38361607	rs2836340	1
chr21	39010116	rs2236436	1
chr21	39914089	rs2837291	1
chr21	40886403	rs13049240	1
chr21	41888378	rs4920102	1
chr21	44835280	rs9808690	1
chr21	45207772	rs2838813	1
chr22	23366646	rs5996528	1
chr22	31096496	rs2074736	1
chr22	34292320	rs1489877	1
chr22	40099469	rs6001762	1
chr22	43122269	rs742134	1
chr22	43986973	rs2294928	1
chr22	44149485	rs5764560	1
chr22	44310934	rs12158741	1
chr22	44924751	rs9614985	1
chr22	45188031	rs9614616	1
chr22	45259963	rs1533371	1
chr22	50473447	rs4824152	1

chr4	84550561	rs1596673	1
chr1	2308567	rs2173049	2
chr1	4801100	rs4654607	2
chr1	4812996	rs10915612	2
chr1	5874176	rs868162	2
chr1	11233902	rs11121704	2
chr1	15768821	rs2271545	2
chr1	17723377	rs11581535	2
chr1	19000319	rs6699706	2
chr1	21553031	rs3753782	2
chr1	21599110	rs10917023	2
chr1	26024891	rs17163470	2
chr1	27405147	rs11548323	2
chr1	27655356	rs12749246	2
chr1	31971667	rs624017	2
chr1	37726624	rs6686248	2
chr1	40500511	rs7512061	2
chr1	54643305	rs10888858	2
chr1	55342470	rs207145	2
chr1	60334990	rs390331	2
chr1	60942867	rs543314	2
chr1	61663460	rs6587943	2
chr1	63082002	rs17124579	2
chr1	67605699	rs787499	2
chr1	70456677	rs1967189	2
chr1	77437296	rs10873941	2
chr1	77438131	rs7545638	2
chr1	80124273	rs12119851	2
chr1	81206156	rs17106149	2
chr1	94115569	rs10782976	2
chr1	94256104	rs12750249	2
chr1	97069794	rs290868	2
chr1	97246005	rs12758854	2
chr1	97449657	rs11165875	2
chr1	98130297	rs1487302	2
chr1	100810988	rs6699536	2
chr1	111588002	rs2800880	2
chr1	111589599	rs12139261	2
chr1	111598795	rs12123811	2
chr1	156135546	rs2485664	2
chr1	160549450	rs3766387	2

chr1	167669014	rs1229363	2
chr1	168608236	rs7517738	2
chr1	168958376	rs10489370	2
chr1	168991048	rs1322487	2
chr1	182135054	rs3845452	2
chr1	182137567	rs3795490	2
chr1	182268623	rs11802583	2
chr1	189053479	rs7546105	2
chr1	196150180	rs12727479	2
chr1	202705069	rs4072661	2
chr1	205267105	rs4951182	2
chr1	205292046	rs1172122	2
chr1	205349855	rs913722	2
chr1	206634569	rs3813965	2
chr1	206771966	rs3024490	2
chr1	208133135	rs17187133	2
chr1	211720313	rs7512315	2
chr1	229053694	rs505557	2
chr1	236598478	rs2298099	2
chr1	237179412	rs12746235	2
chr1	242538265	rs11804678	2
chr1	245486539	rs12089406	2
chr1	246799717	rs3935608	2
chr2	855459	rs11682609	2
chr2	3078332	rs11127398	2
chr2	6215243	rs12464218	2
chr2	10472504	rs818167	2
chr2	14998162	rs6432496	2
chr2	18480716	rs1489689	2
chr2	19524342	rs13407212	2
chr2	19539249	rs3914966	2
chr2	21460413	rs6749957	2
chr2	29087182	rs7568662	2
chr2	30144358	rs1509578	2
chr2	30692520	rs530076	2
chr2	32270402	rs409188	2
chr2	32863477	rs6759807	2
chr2	37245661	rs2110965	2
chr2	37477793	rs957982	2
chr2	40064728	rs17025029	2
chr2	41878491	rs10204805	2

chr2	52325885	rs12468211	2
chr2	53429466	rs1521943	2
chr2	55667112	rs782637	2
chr2	67285986	rs13385154	2
chr2	67413146	rs13424211	2
chr2	78951004	rs11126689	2
chr2	79232860	rs1434194	2
chr2	80517172	rs744957	2
chr2	100061736	rs11695782	2
chr2	103993282	rs935070	2
chr2	104072986	rs2376064	2
chr2	104098868	rs11896903	2
chr2	114461398	rs4849337	2
chr2	114985993	rs7603116	2
chr2	126586570	rs10204012	2
chr2	126891370	rs11899448	2
chr2	129375792	rs2030664	2
chr2	133819684	rs7603317	2
chr2	137333487	rs6740175	2
chr2	151083575	rs12692951	2
chr2	154194291	rs6435067	2
chr2	154242633	rs9288324	2
chr2	154314345	rs7566164	2
chr2	155627317	rs843236	2
chr2	157806765	rs17798043	2
chr2	158220317	rs6732439	2
chr2	158235629	rs12994903	2
chr2	169415592	rs10168193	2
chr2	176517861	rs2969344	2
chr2	178293219	rs836882	2
chr2	180037368	rs12468587	2
chr2	180760937	rs4362518	2
chr2	181583683	rs12105941	2
chr2	201343270	rs7577057	2
chr2	214016128	rs6749347	2
chr2	217229157	rs6435966	2
chr2	222132385	rs2894450	2
chr2	223247821	rs4674771	2
chr2	228465605	rs7592831	2
chr2	228878293	rs7578485	2
chr2	230682460	rs17762352	2

chr2	232879311	rs709937	2
chr2	237537599	rs10186871	2
chr2	239118750	rs3791428	2
chr2	239123654	rs3791446	2
chr2	239725708	rs6734016	2
chr2	240228496	rs7420256	2
chr3	1150935	rs2036236	2
chr3	1280141	rs17037117	2
chr3	2038877	rs6767146	2
chr3	5295442	rs1391961	2
chr3	6097693	rs17288023	2
chr3	8428098	rs359006	2
chr3	10294102	rs2619507	2
chr3	14309159	rs4684204	2
chr3	20528881	rs1403651	2
chr3	20747089	rs964911	2
chr3	21978302	rs2291818	2
chr3	23146552	rs9879958	2
chr3	23344386	rs13076527	2
chr3	24723009	rs9310754	2
chr3	24724765	rs6794687	2
chr3	28078149	rs4680899	2
chr3	30156401	rs7638300	2
chr3	30166176	rs1433986	2
chr3	30807569	rs1393751	2
chr3	46748907	rs7614762	2
chr3	48503583	rs7434077	2
chr3	48621576	rs9834639	2
chr3	52036939	rs9848727	2
chr3	62045175	rs9311834	2
chr3	62138804	rs2366684	2
chr3	63405065	rs1901304	2
chr3	65316647	rs1479958	2
chr3	66746608	rs9845175	2
chr3	67410572	rs6782194	2
chr3	68316729	rs1491758	2
chr3	69062361	rs9851078	2
chr3	70121641	rs13084430	2
chr3	87358096	rs9861650	2
chr3	88413455	rs1458005	2
chr3	97056677	rs928958	2

chr3	102972148	rs17779020	2
chr3	105003955	rs6797921	2
chr3	114233314	rs2279828	2
chr3	116409558	rs4831139	2
chr3	123069512	rs9873177	2
chr3	126254742	rs10934761	2
chr3	131557875	rs12639456	2
chr3	133579276	rs6781238	2
chr3	133623144	rs10935070	2
chr3	133756878	rs1799852	2
chr3	140405774	rs10513112	2
chr3	140523972	rs349567	2
chr3	143294361	rs6806500	2
chr3	143425726	rs1554672	2
chr3	148495609	rs3915005	2
chr3	152755987	rs1381094	2
chr3	158904996	rs17646246	2
chr3	163855119	rs6774925	2
chr3	165053083	rs9883965	2
chr3	165550120	rs11917072	2
chr3	166566369	rs522409	2
chr3	166762771	rs9874962	2
chr3	166977837	rs1608323	2
chr3	167070719	rs6773278	2
chr3	167631050	rs7627289	2
chr3	171803823	rs13075194	2
chr3	174684796	rs12493995	2
chr3	176535281	rs7636407	2
chr3	179310025	rs6794192	2
chr3	180071246	rs9868787	2
chr3	186185593	rs741483	2
chr3	186964439	rs11712248	2
chr3	187132898	rs2270354	2
chr3	188908099	rs6797432	2
chr3	191571662	rs4074485	2
chr3	192142746	rs1460924	2
chr3	192376955	rs12632797	2
chr3	194749262	rs9881429	2
chr4	7156740	rs10034147	2
chr4	7182257	rs7692512	2
chr4	11305279	rs17468208	2

chr4	11502237	rs924000	2
chr4	13219766	rs6838271	2
chr4	16493328	rs1501141	2
chr4	21115590	rs16870429	2
chr4	21502990	rs187196	2
chr4	31211185	rs1875050	2
chr4	34560067	rs1480990	2
chr4	35247225	rs7686175	2
chr4	37263209	rs3910363	2
chr4	37392774	rs17421722	2
chr4	37479065	rs1382979	2
chr4	37857269	rs6855439	2
chr4	52052572	rs2860055	2
chr4	54777238	rs2955174	2
chr4	61214497	rs6845545	2
chr4	61238394	rs1827770	2
chr4	61244052	rs2342453	2
chr4	62001735	rs13115125	2
chr4	63468092	rs10021833	2
chr4	70196125	rs1383436	2
chr4	72508307	rs788930	2
chr4	72755212	rs7683184	2
chr4	73426690	rs16849364	2
chr4	74150033	rs7660423	2
chr4	75004148	rs11098369	2
chr4	76806352	rs13124337	2
chr4	77271165	rs10491463	2
chr4	77348973	rs11936421	2
chr4	82739689	rs17354029	2
chr4	82743054	rs7679085	2
chr4	83715043	rs17007216	2
chr4	87811759	rs1054628	2
chr4	88172912	rs2622629	2
chr4	90048414	rs11929845	2
chr4	94658554	rs11097432	2
chr4	100289558	rs4235450	2
chr4	105089276	rs12509636	2
chr4	106378884	rs940618	2
chr4	107867524	rs11940819	2
chr4	114492457	rs7698391	2
chr4	120788775	rs13111620	2

chr4	123670948	rs2589840	2
chr4	129626198	rs10001198	2
chr4	132325751	rs10518610	2
chr4	134529832	rs10005804	2
chr4	139178614	rs1471928	2
chr4	140114972	rs11100497	2
chr4	148226671	rs3910044	2
chr4	152951291	rs6836054	2
chr4	154074071	rs2404917	2
chr4	162404259	rs17042479	2
chr4	164370625	rs2102296	2
chr4	164384737	rs7658861	2
chr4	164443105	rs12152616	2
chr4	164638911	rs9790550	2
chr4	167800352	rs13124475	2
chr4	168421369	rs2014622	2
chr4	172230668	rs9312518	2
chr4	172855932	rs6833917	2
chr4	172871712	rs17058978	2
chr4	178306726	rs1567474	2
chr4	180582886	rs1422505	2
chr4	181292756	rs4861940	2
chr4	183679448	rs4241779	2
chr4	184441883	rs793794	2
chr4	185290827	rs1288542	2
chr4	187577436	rs11734029	2
chr5	4588380	rs1541834	2
chr5	7876175	rs326121	2
chr5	8061839	rs9313221	2
chr5	9373336	rs42192	2
chr5	11487473	rs26150	2
chr5	16186665	rs342561	2
chr5	16588931	rs2451852	2
chr5	17199965	rs11952802	2
chr5	20443591	rs6877123	2
chr5	23371054	rs10058259	2
chr5	31713339	rs7725382	2
chr5	31736475	rs375183	2
chr5	32596740	rs11952155	2
chr5	36482728	rs2468532	2
chr5	36520411	rs4869483	2

chr5	36541200	rs9292633	2
chr5	39435122	rs2887029	2
chr5	56056943	rs10042443	2
chr5	62737212	rs17471355	2
chr5	62798207	rs7708428	2
chr5	66450712	rs13180603	2
chr5	73945145	rs283592	2
chr5	74012518	rs194274	2
chr5	74273438	rs569120	2
chr5	74423835	rs460738	2
chr5	86451054	rs1500258	2
chr5	100878913	rs13355889	2
chr5	104991691	rs17500322	2
chr5	110035287	rs2900041	2
chr5	110403370	rs11241068	2
chr5	121908825	rs12652819	2
chr5	125867587	rs389333	2
chr5	126342662	rs6595693	2
chr5	126627359	rs11958842	2
chr5	133099880	rs4367292	2
chr5	133521361	rs155578	2
chr5	135753963	rs7380956	2
chr5	137017151	rs12188192	2
chr5	137086669	rs2158409	2
chr5	143502124	rs4912922	2
chr5	143645062	rs12657648	2
chr5	143657295	rs1427864	2
chr5	144378814	rs10515542	2
chr5	145115832	rs12109366	2
chr5	148124257	rs1422993	2
chr5	154532516	rs7703330	2
chr5	161263574	rs173565	2
chr5	168430999	rs729149	2
chr5	169025472	rs6890327	2
chr5	169037736	rs1059160	2
chr5	169092640	rs11744892	2
chr5	172042829	rs6555988	2
chr5	172549963	rs7442753	2
chr5	174120380	rs11739519	2
chr5	177992966	rs1800197	2
chr6	730114	rs7758338	2

chr6	4514241	rs6926616	2
chr6	4515317	rs11242955	2
chr6	4710828	rs1849564	2
chr6	5908931	rs7775306	2
chr6	6009615	rs3763180	2
chr6	6145459	rs1050783	2
chr6	8324224	rs2792613	2
chr6	8357004	rs1572852	2
chr6	8901291	rs9378578	2
chr6	13137533	rs10485363	2
chr6	13650739	rs6458997	2
chr6	14407873	rs9476556	2
chr6	18515745	rs6459621	2
chr6	20901080	rs6456382	2
chr6	25181219	rs9358839	2
chr6	25768796	rs1892250	2
chr6	26608097	rs6919391	2
chr6	27021213	rs7741445	2
chr6	27259186	rs9393796	2
chr6	29001153	rs9257453	2
chr6	29216883	rs3116837	2
chr6	29572427	rs1003581	2
chr6	30106070	rs2844795	2
chr6	31084350	rs2535315	2
chr6	31090563	rs3130544	2
chr6	31202737	rs9263870	2
chr6	31306916	rs3094691	2
chr6	31353434	rs2596501	2
chr6	31379160	rs9501587	2
chr6	31719231	rs1065356	2
chr6	31903079	rs9267665	2
chr6	32762309	rs2051549	2
chr6	32832447	rs2228397	2
chr6	35173084	rs942375	2
chr6	39261987	rs970392	2
chr6	40019576	rs4714285	2
chr6	41653533	rs2230088	2
chr6	41657691	rs2746183	2
chr6	41659325	rs1474761	2
chr6	43831165	rs9381257	2
chr6	44472985	rs9395021	2

chr6	45627709	rs12663992	2
chr6	52692829	rs2180323	2
chr6	67020800	rs10944945	2
chr6	69677201	rs6455338	2
chr6	79723374	rs9448767	2
chr6	84872588	rs9351013	2
chr6	89945903	rs9351230	2
chr6	90792487	rs17523287	2
chr6	91685330	rs575844	2
chr6	92449257	rs878519	2
chr6	96124872	rs11754559	2
chr6	99991133	rs2001456	2
chr6	105627372	rs11152903	2
chr6	115267315	rs996139	2
chr6	118211386	rs205915	2
chr6	123896568	rs11154196	2
chr6	125133108	rs7748434	2
chr6	126160262	rs1330674	2
chr6	128630162	rs17056140	2
chr6	131805180	rs6935458	2
chr6	133202650	rs477094	2
chr6	133219057	rs9402490	2
chr6	137219938	rs1327474	2
chr6	143022633	rs200145	2
chr6	143610595	rs6570567	2
chr6	143997861	rs4552753	2
chr6	144061157	rs2328542	2
chr6	147894552	rs4385334	2
chr6	151599637	rs1340874	2
chr6	158708519	rs9456341	2
chr6	159527839	rs395538	2
chr6	160421693	rs4708867	2
chr6	161495448	rs9458289	2
chr6	161546869	rs12526148	2
chr6	164262682	rs794108	2
chr6	170210831	rs9366181	2
chr7	2208837	rs1612548	2
chr7	3553933	rs13221445	2
chr7	5578606	rs2693946	2
chr7	10852858	rs6965606	2
chr7	12102651	rs6963473	2

chr7	12160434	rs6952272	2
chr7	12218340	rs3887296	2
chr7	12244161	rs1990622	2
chr7	13236436	rs10488256	2
chr7	14176817	rs17167945	2
chr7	19940344	rs6947148	2
chr7	25036493	rs10276962	2
chr7	26190552	rs1049938	2
chr7	26868977	rs10235680	2
chr7	27313680	rs1005312	2
chr7	29205143	rs3812389	2
chr7	29304852	rs3812333	2
chr7	29332423	rs39132	2
chr7	29341533	rs986226	2
chr7	29407425	rs7800059	2
chr7	29651597	rs1728539	2
chr7	31707635	rs12669290	2
chr7	33368282	rs3823763	2
chr7	33919627	rs1427483	2
chr7	37923216	rs6964434	2
chr7	38024480	rs6462801	2
chr7	38539779	rs12538952	2
chr7	45644205	rs9639925	2
chr7	48147035	rs10081256	2
chr7	48527548	rs4072502	2
chr7	51884689	rs7801857	2
chr7	74516081	rs2267824	2
chr7	79956135	rs10229206	2
chr7	83931990	rs701307	2
chr7	86250461	rs1860784	2
chr7	94301977	rs2106295	2
chr7	94411687	rs42523	2
chr7	104629171	rs759532	2
chr7	105972360	rs2727758	2
chr7	108434322	rs2284290	2
chr7	112167721	rs2523017	2
chr7	126513322	rs2237734	2
chr7	127656948	rs806172	2
chr7	127926425	rs12706823	2
chr7	129529642	rs4574773	2
chr7	129773348	rs4626538	2

chr7	135286658	rs11762667	2
chr7	136665993	rs10235715	2
chr7	137305974	rs12666148	2
chr7	137321061	rs2242045	2
chr7	137431539	rs918892	2
chr7	138269674	rs6963140	2
chr7	144783456	rs1635357	2
chr7	145144438	rs10487524	2
chr7	145911936	rs4392813	2
chr7	145979789	rs7777578	2
chr7	155905561	rs6459961	2
chr7	155967896	rs6459975	2
chr7	156223532	rs1922086	2
chr7	156784620	rs2270264	2
chr7	157295131	rs757854	2
chr8	878266	rs11777864	2
chr8	1003948	rs13251306	2
chr8	1183355	rs12334702	2
chr8	6839928	rs2977798	2
chr8	8555870	rs2294143	2
chr8	8690132	rs17684514	2
chr8	8801528	rs3827811	2
chr8	10258211	rs4504638	2
chr8	10456707	rs7017116	2
chr8	10648603	rs7463228	2
chr8	12989398	rs6531017	2
chr8	15779883	rs1362867	2
chr8	18021883	rs208021	2
chr8	19473424	rs10503653	2
chr8	19899302	rs10102717	2
chr8	22917906	rs2466215	2
chr8	39083415	rs4130393	2
chr8	39728839	rs201995	2
chr8	46266587	rs6651412	2
chr8	47670402	rs7842068	2
chr8	51530148	rs7003517	2
chr8	57066755	rs16921553	2
chr8	57901056	rs2004902	2
chr8	69669870	rs16936267	2
chr8	71176937	rs4103014	2
chr8	73434627	rs4738353	2

chr8	73557907	rs10087203	2
chr8	73626463	rs10086512	2
chr8	73757174	rs12545117	2
chr8	74568268	rs7015712	2
chr8	76722558	rs966535	2
chr8	77161703	rs12675877	2
chr8	82662197	rs7833831	2
chr8	82778844	rs7465344	2
chr8	86812523	rs7011051	2
chr8	100585058	rs16898600	2
chr8	100588738	rs2507780	2
chr8	101050430	rs2122922	2
chr8	104629394	rs4236775	2
chr8	115683666	rs17729317	2
chr8	118103756	rs11562695	2
chr8	118307229	rs16890815	2
chr8	122285462	rs4871240	2
chr8	124688684	rs2382993	2
chr8	125105917	rs4871570	2
chr8	125260404	rs10283134	2
chr8	125317088	rs13260832	2
chr8	133523845	rs4736691	2
chr8	134504037	rs4366044	2
chr8	134519905	rs4909456	2
chr8	134529841	rs1550582	2
chr8	136767016	rs2610088	2
chr8	138060575	rs10101858	2
chr8	139120605	rs11166878	2
chr8	141611385	rs1004380	2
chr8	142593861	rs7843869	2
chr8	143580905	rs1809148	2
chr9	953465	rs10759073	2
chr9	991114	rs279877	2
chr9	2024076	rs6475412	2
chr9	3828745	rs17742098	2
chr9	4347422	rs7034125	2
chr9	4352193	rs11789687	2
chr9	4357693	rs12005536	2
chr9	4543307	rs2026828	2
chr9	4658449	rs1385452	2
chr9	4844459	rs159432	2

chr9	6910916	rs12235748	2
chr9	7379168	rs7871958	2
chr9	7829623	rs9299057	2
chr9	8468780	rs13286656	2
chr9	9229566	rs17667884	2
chr9	9264932	rs9299090	2
chr9	9283557	rs10122609	2
chr9	14562632	rs1556029	2
chr9	14880669	rs7862716	2
chr9	18332002	rs10810957	2
chr9	18656178	rs776780	2
chr9	24605823	rs1162608	2
chr9	24617371	rs1184457	2
chr9	28804100	rs824230	2
chr9	29661361	rs10511831	2
chr9	32748367	rs12551778	2
chr9	69104702	rs4744826	2
chr9	69388120	rs867366	2
chr9	69812567	rs4744942	2
chr9	75493953	rs1327369	2
chr9	75498753	rs2778899	2
chr9	76607668	rs1952461	2
chr9	77000825	rs4745607	2
chr9	79957293	rs962883	2
chr9	80336313	rs7852248	2
chr9	82735797	rs4877702	2
chr9	83609667	rs11792478	2
chr9	83717928	rs11793897	2
chr9	88276199	rs4877434	2
chr9	90342047	rs7048728	2
chr9	91184097	rs10991808	2
chr9	101770154	rs16920725	2
chr9	106965791	rs6477551	2
chr9	107143332	rs956578	2
chr9	108749712	rs10816710	2
chr9	110706320	rs4623510	2
chr9	110834829	rs2821153	2
chr9	113188426	rs10435864	2
chr9	113231643	rs10513190	2
chr9	113360046	rs1077636	2
chr9	113624974	rs540599	2

chr9	114270168	rs2636864	2
chr9	115223607	rs11794549	2
chr9	115979439	rs1349323	2
chr9	117785004	rs12352822	2
chr9	124262893	rs2282087	2
chr9	124835397	rs4838224	2
chr9	126724703	rs3850586	2
chr9	126887077	rs3120029	2
chr9	127127444	rs1281156	2
chr9	130908179	rs4740383	2
chr9	132423288	rs7470548	2
chr9	135984004	rs2152174	2
chr10	1663053	rs1909428	2
chr10	2093616	rs7101173	2
chr10	3111696	rs10508245	2
chr10	3657428	rs10904068	2
chr10	3820322	rs11252121	2
chr10	4768369	rs1901633	2
chr10	4771019	rs1901632	2
chr10	5077352	rs7901781	2
chr10	5244199	rs9423393	2
chr10	5400010	rs10795273	2
chr10	6234611	rs1064891	2
chr10	6239257	rs11599520	2
chr10	6721787	rs1327690	2
chr10	7655110	rs17142892	2
chr10	8927839	rs1243411	2
chr10	8978271	rs827628	2
chr10	9665689	rs10905601	2
chr10	9808336	rs11256348	2
chr10	13015323	rs11258127	2
chr10	16096490	rs11253779	2
chr10	19865320	rs10508599	2
chr10	19904868	rs11592239	2
chr10	22242274	rs12264839	2
chr10	24005138	rs2027238	2
chr10	24010425	rs12146261	2
chr10	25855536	rs10508707	2
chr10	26415585	rs945296	2
chr10	27404376	rs1334893	2
chr10	30109862	rs2505115	2

chr10	30629495	rs10826831	2
chr10	30726604	rs4080824	2
chr10	35600599	rs660301	2
chr10	43889310	rs12218783	2
chr10	45219921	rs1815630	2
chr10	52368653	rs1194683	2
chr10	54259434	rs2043995	2
chr10	54355097	rs11594789	2
chr10	60126288	rs12761506	2
chr10	61246113	rs1906470	2
chr10	65865461	rs16922054	2
chr10	66821589	rs12763505	2
chr10	75496823	rs3012041	2
chr10	76362971	rs12766217	2
chr10	76389285	rs7909650	2
chr10	76395529	rs749127	2
chr10	79955982	rs2146192	2
chr10	80668365	rs7079135	2
chr10	81165287	rs12781461	2
chr10	84871785	rs11201267	2
chr10	87578876	rs9664222	2
chr10	88830989	rs10736363	2
chr10	90833199	rs12249377	2
chr10	91131307	rs7067899	2
chr10	95530235	rs11188346	2
chr10	95556283	rs4918948	2
chr10	95630809	rs12773566	2
chr10	101267649	rs2863114	2
chr10	101269303	rs10883603	2
chr10	105522573	rs11192469	2
chr10	106249184	rs10509813	2
chr10	116107992	rs2694798	2
chr10	116765705	rs1615201	2
chr10	117994452	rs10886189	2
chr10	118604432	rs4751667	2
chr10	124647128	rs12411439	2
chr10	125711864	rs7909932	2
chr10	128433760	rs4556457	2
chr10	129876988	rs7076708	2
chr10	130829731	rs7893625	2
chr11	4180411	rs2044138	2

chr11	4959451	rs3850508	2
chr11	7156596	rs3105717	2
chr11	11423943	rs901545	2
chr11	12504378	rs11547363	2
chr11	13094185	rs1899302	2
chr11	17922329	rs2283240	2
chr11	18143140	rs2445162	2
chr11	21508216	rs12574030	2
chr11	21511321	rs4074586	2
chr11	21587820	rs11602745	2
chr11	25842003	rs1869020	2
chr11	26151223	rs4072100	2
chr11	26993672	rs16916399	2
chr11	30553091	rs16920925	2
chr11	32066554	rs11828792	2
chr11	32555152	rs1872706	2
chr11	34179738	rs910257	2
chr11	45395539	rs2863706	2
chr11	45463950	rs901901	2
chr11	45585433	rs1488682	2
chr11	56194765	rs1384061	2
chr11	56310347	rs11227836	2
chr11	60965272	rs548363	2
chr11	64329761	rs694739	2
chr11	69025738	rs11228444	2
chr11	70349076	rs7109643	2
chr11	70496312	rs573795	2
chr11	71165389	rs7124728	2
chr11	78008566	rs10899426	2
chr11	84842764	rs952201	2
chr11	86460412	rs618513	2
chr11	86781798	rs744293	2
chr11	86793314	rs12799567	2
chr11	100022550	rs698698	2
chr11	100052909	rs11222552	2
chr11	100143170	rs2012260	2
chr11	100170688	rs2044983	2
chr11	103695668	rs7924591	2
chr11	104837005	rs642603	2
chr11	105053519	rs1785883	2
chr11	108632945	rs10890873	2

chr11	113278920	rs668204	2
chr11	116275904	rs876411	2
chr11	118939696	rs4938589	2
chr11	119027609	rs741811	2
chr11	120853309	rs6589848	2
chr11	125118620	rs608393	2
chr11	128375436	rs11221267	2
chr11	130405451	rs2242312	2
chr11	131431722	rs12790085	2
chr11	132816089	rs12285031	2
chr11	132833690	rs4937729	2
chr11	133907493	rs1106561	2
chr11	134612572	rs1944866	2
chr12	3115820	rs10774120	2
chr12	3216677	rs887363	2
chr12	4540899	rs10849087	2
chr12	7538538	rs7136716	2
chr12	7570221	rs1419974	2
chr12	11597203	rs11054362	2
chr12	12930203	rs1291352	2
chr12	18916925	rs16915276	2
chr12	20637424	rs7304868	2
chr12	25683814	rs10842596	2
chr12	26619892	rs11611622	2
chr12	27602525	rs12319361	2
chr12	27644972	rs7295096	2
chr12	28871601	rs7294633	2
chr12	31204168	rs10492354	2
chr12	31953515	rs17510714	2
chr12	31967313	rs10771892	2
chr12	41403584	rs12305552	2
chr12	43066689	rs1183790	2
chr12	43780963	rs4251580	2
chr12	51262867	rs4761815	2
chr12	58086924	rs7978954	2
chr12	58833772	rs17121530	2
chr12	66904238	rs10506515	2
chr12	75664031	rs10879956	2
chr12	76043993	rs2043369	2
chr12	89381409	rs12318428	2
chr12	92133514	rs709219	2

chr12	94089430	rs1434704	2
chr12	95966695	rs10507068	2
chr12	97393283	rs1861370	2
chr12	101677741	rs10778137	2
chr12	103049487	rs11111423	2
chr12	107163334	rs2374671	2
chr12	107264444	rs12322483	2
chr12	125442792	rs988535	2
chr12	125748335	rs11058317	2
chr12	129358045	rs10847836	2
chr12	129369883	rs900263	2
chr12	129409961	rs1976829	2
chr12	129517949	rs12321897	2
chr13	20456753	rs3936015	2
chr13	22285269	rs2038711	2
chr13	24934511	rs2084756	2
chr13	26992117	rs9581798	2
chr13	27360569	rs9507881	2
chr13	28472190	rs615529	2
chr13	28489832	rs600640	2
chr13	30429481	rs1141362	2
chr13	30800149	rs9315071	2
chr13	34617439	rs2026064	2
chr13	38747305	rs11147730	2
chr13	39623967	rs2324452	2
chr13	41180534	rs1999507	2
chr13	43128003	rs2657107	2
chr13	43700594	rs393632	2
chr13	44897554	rs9534002	2
chr13	46176746	rs17601960	2
chr13	46871568	rs9316235	2
chr13	48676905	rs9285169	2
chr13	48748046	rs9591208	2
chr13	49409673	rs12429253	2
chr13	49651694	rs1535468	2
chr13	62706741	rs4884429	2
chr13	62828217	rs1402552	2
chr13	74187563	rs7988935	2
chr13	77338047	rs7982454	2
chr13	80031651	rs7995973	2
chr13	82031535	rs12184745	2

chr13	82056760	rs9531286	2
chr13	85306989	rs1873288	2
chr13	86007732	rs4550343	2
chr13	88365392	rs1074201	2
chr13	90084010	rs2805713	2
chr13	91433032	rs12863915	2
chr13	97407088	rs10851072	2
chr13	98111979	rs9513355	2
chr13	102147884	rs17632458	2
chr13	106568505	rs3759453	2
chr14	21585739	rs6571925	2
chr14	22703080	rs4982652	2
chr14	24434758	rs12891613	2
chr14	30338688	rs7160082	2
chr14	31083414	rs10483353	2
chr14	32382339	rs942028	2
chr14	42866381	rs17541557	2
chr14	58780541	rs17095093	2
chr14	63768838	rs7157785	2
chr14	64799363	rs1268663	2
chr14	64921525	rs10138506	2
chr14	66496531	rs3737160	2
chr14	67251254	rs7152903	2
chr14	68738840	rs6573856	2
chr14	69243212	rs10148349	2
chr14	70882372	rs8023177	2
chr14	78003171	rs1955568	2
chr14	79834334	rs2293829	2
chr14	80310393	rs178954	2
chr14	86826832	rs1642272	2
chr14	88028231	rs10151371	2
chr14	91678893	rs6575209	2
chr14	91690317	rs11160024	2
chr14	92264068	rs7156630	2
chr14	94470627	rs8014978	2
chr14	95270110	rs1884535	2
chr14	95794905	rs1951981	2
chr14	100732404	rs2273608	2
chr14	104341375	rs8016091	2
chr14	104373479	rs4074324	2
chr14	105532207	rs4074453	2

chr15	26265263	rs7183534	2
chr15	35774872	rs1846135	2
chr15	40170579	rs1801389	2
chr15	47394393	rs11070582	2
chr15	48720814	rs784417	2
chr15	52695991	rs7161887	2
chr15	53493736	rs952993	2
chr15	53552900	rs1906426	2
chr15	53606991	rs10152972	2
chr15	54504394	rs1520410	2
chr15	54506655	rs537389	2
chr15	59479763	rs7161900	2
chr15	61297886	rs4461014	2
chr15	62109283	rs2083013	2
chr15	62173190	rs17205603	2
chr15	62378487	rs289094	2
chr15	62929031	rs4774458	2
chr15	63142997	rs12899931	2
chr15	66804320	rs7183242	2
chr15	71253178	rs10518937	2
chr15	71262825	rs4777352	2
chr15	71827228	rs10518970	2
chr15	86975681	rs2082538	2
chr15	88355438	rs6496475	2
chr15	90154903	rs11853144	2
chr15	90808700	rs389480	2
chr15	90839685	rs2532101	2
chr15	91675087	rs7402600	2
chr15	92645641	rs3743360	2
chr15	95751875	rs8025900	2
chr15	96296956	rs4984430	2
chr15	97151390	rs12912621	2
chr15	97879115	rs7497359	2
chr15	98334634	rs4965807	2
chr15	98968644	rs2715423	2
chr15	101427596	rs4965384	2
chr16	4354619	rs10974	2
chr16	4634447	rs1659506	2
chr16	4829254	rs7186281	2
chr16	4941053	rs2908659	2
chr16	7102864	rs11640267	2

chr16	7176672	rs12935311	2
chr16	18933753	rs9934555	2
chr16	24309866	rs2238510	2
chr16	52027135	rs12149256	2
chr16	67648677	rs9972635	2
chr16	73678273	rs8063377	2
chr16	73743658	rs367823	2
chr16	74192322	rs807293	2
chr16	82579883	rs17245633	2
chr16	83011060	rs12925746	2
chr16	83087005	rs6565116	2
chr16	83857280	rs450204	2
chr16	83862522	rs1123075	2
chr16	84870122	rs4782677	2
chr16	85507778	rs8053670	2
chr16	85524373	rs7500151	2
chr16	86089016	rs13336935	2
chr16	89842993	rs12599531	2
chr17	1961455	rs9646401	2
chr17	1980517	rs7207686	2
chr17	7376950	rs4796412	2
chr17	8908361	rs726679	2
chr17	9252737	rs6503190	2
chr17	9658226	rs429068	2
chr17	13252736	rs11651762	2
chr17	31677115	rs425083	2
chr17	32210771	rs2069145	2
chr17	35193098	rs17547201	2
chr17	38283293	rs4329955	2
chr17	39209446	rs486512	2
chr17	40563547	rs3136685	2
chr17	41647748	rs6503650	2
chr17	44336113	rs7216307	2
chr17	44973977	rs9908256	2
chr17	44980217	rs11871217	2
chr17	45138914	rs4986172	2
chr17	45271809	rs7213493	2
chr17	45325639	rs4792814	2
chr17	46915752	rs1662596	2
chr17	47194314	rs858671	2
chr17	49278661	rs2898857	2

chr17	52440377	rs4566219	2
chr17	53210648	rs807101	2
chr17	57535839	rs12602441	2
chr17	59433712	rs4968363	2
chr17	61211535	rs1860373	2
chr17	73711840	rs2683173	2
chr17	79196371	rs2703549	2
chr17	80394251	rs8359	2
chr17	81091850	rs4969384	2
chr18	4070978	rs9789161	2
chr18	4545834	rs7238906	2
chr18	6306122	rs1946753	2
chr18	6384432	rs1436907	2
chr18	23386675	rs11659634	2
chr18	24632226	rs339858	2
chr18	36118973	rs1944320	2
chr18	38541190	rs1426685	2
chr18	40080773	rs749684	2
chr18	42797879	rs1943177	2
chr18	42891456	rs669924	2
chr18	45418207	rs2612566	2
chr18	45682394	rs3745009	2
chr18	48461304	rs1877412	2
chr18	48476387	rs4940383	2
chr18	50128582	rs17716604	2
chr18	59435094	rs9954602	2
chr18	59475548	rs656750	2
chr18	59546924	rs3133202	2
chr18	63017447	rs12958400	2
chr18	63669134	rs12960185	2
chr18	65484712	rs8091665	2
chr18	65506098	rs12965811	2
chr18	67997725	rs3862732	2
chr18	68778039	rs677592	2
chr18	69583277	rs4381690	2
chr18	69595900	rs10401068	2
chr18	72635086	rs4892018	2
chr18	75104476	rs894575	2
chr18	75303428	rs4891260	2
chr18	76704130	rs9953092	2
chr18	76720436	rs4393659	2

chr18	78677601	rs2941805	2
chr19	3421522	rs6510747	2
chr19	4635390	rs17363184	2
chr19	4804512	rs8102626	2
chr19	8831041	rs2910368	2
chr19	16758152	rs773845	2
chr19	19678719	rs2304130	2
chr19	22584928	rs10420442	2
chr19	23494599	rs17000180	2
chr19	23580362	rs9305005	2
chr19	24163222	rs10500223	2
chr19	29049890	rs6509207	2
chr19	29073254	rs10422290	2
chr19	29185867	rs10518258	2
chr19	30180286	rs2866721	2
chr19	46256322	rs10406931	2
chr19	51683264	rs2902883	2
chr19	52396303	rs324119	2
chr19	52531345	rs781129	2
chr19	53539198	rs3746305	2
chr19	57153977	rs10416425	2
chr20	100014	rs2196239	2
chr20	372441	rs742605	2
chr20	665209	rs6053798	2
chr20	3846662	rs4815617	2
chr20	6220602	rs7261122	2
chr20	8163993	rs978266	2
chr20	8181631	rs6055603	2
chr20	8512003	rs6086477	2
chr20	10255278	rs3025866	2
chr20	10262603	rs362563	2
chr20	15082304	rs383310	2
chr20	19940543	rs199549	2
chr20	19949783	rs11699866	2
chr20	20260931	rs996569	2
chr20	37526060	rs12481150	2
chr20	39129026	rs2868502	2
chr20	39661823	rs1655353	2
chr20	39671934	rs11698332	2
chr20	45268950	rs8113868	2
chr20	48159082	rs827944	2

chr20	51047504	rs6067620	2
chr20	53634337	rs204767	2
chr20	53687590	rs6063977	2
chr20	53907007	rs6022842	2
chr20	56981100	rs1276466	2
chr20	57015473	rs1299153	2
chr20	57086493	rs6069979	2
chr21	19917101	rs2825891	2
chr21	23228066	rs2827974	2
chr21	24236321	rs2828925	2
chr21	25367698	rs2151	2
chr21	26427688	rs11702375	2
chr21	30463521	rs8134027	2
chr21	31729686	rs13052593	2
chr21	33355024	rs1041868	2
chr21	35061303	rs9636889	2
chr21	39199320	rs1041439	2
chr21	39712884	rs12233347	2
chr21	39888335	rs17753847	2
chr21	41887220	rs2282109	2
chr21	42142587	rs7280633	2
chr21	45439803	rs9980531	2
chr21	46004432	rs9254	2
chr22	17556734	rs174348	2
chr22	22032219	rs9610900	2
chr22	26337333	rs6005016	2
chr22	26597742	rs5761618	2
chr22	29142480	rs713728	2
chr22	30274004	rs6006427	2
chr22	31118491	rs2017301	2
chr22	31155174	rs4820946	2
chr22	37234748	rs1476002	2
chr22	42477526	rs13057831	2
chr22	44203651	rs139171	2
chr22	45506239	rs13433666	2
chr22	47168657	rs5767557	2
chr22	47514518	rs1297370	2
chr4	25560828	rs35780192	2
chr1	2332449	rs903904	3
chr1	2350287	rs7527871	3
chr1	6110928	rs12142108	3

chr1	10144128	rs12144133	3
chr1	10549448	rs12120962	3
chr1	13953018	rs10927969	3
chr1	13954956	rs6678469	3
chr1	22113027	rs4654783	3
chr1	24307661	rs4648973	3
chr1	29277487	rs10799128	3
chr1	30697390	rs1188441	3
chr1	36689392	rs535175	3
chr1	40585561	rs16865687	3
chr1	51285237	rs474668	3
chr1	57719370	rs12030998	3
chr1	63171641	rs4915878	3
chr1	66634626	rs6696812	3
chr1	66723146	rs7515848	3
chr1	67566197	rs2153527	3
chr1	75364753	rs1249849	3
chr1	76387055	rs1925359	3
chr1	83517569	rs7553619	3
chr1	83751476	rs761401	3
chr1	95168166	rs965314	3
chr1	98398204	rs3908575	3
chr1	101798353	rs10493976	3
chr1	106091846	rs17016721	3
chr1	109939713	rs11589677	3
chr1	115292879	rs910330	3
chr1	116514108	rs10802189	3
chr1	158923642	rs11805756	3
chr1	159593693	rs1446966	3
chr1	161492938	rs4657039	3
chr1	161997497	rs6691548	3
chr1	167657588	rs1229378	3
chr1	177851726	rs1359939	3
chr1	201821142	rs1022361	3
chr1	201917520	rs2047263	3
chr1	205269147	rs1768586	3
chr1	205314715	rs12120958	3
chr1	208494541	rs841497	3
chr1	209699245	rs10863782	3
chr1	212458051	rs6657001	3
chr1	213688305	rs1509868	3

chr1	229023896	rs537250	3
chr1	232615499	rs2039490	3
chr1	233846278	rs1389971	3
chr1	244407083	rs12084013	3
chr2	36787	rs11900053	3
chr2	4354592	rs6542705	3
chr2	5832934	rs2163027	3
chr2	8840890	rs11894892	3
chr2	17613087	rs12478869	3
chr2	18376297	rs12611619	3
chr2	18651080	rs12053481	3
chr2	25255584	rs1465825	3
chr2	29169814	rs12620500	3
chr2	29331629	rs17007904	3
chr2	40421944	rs13414307	3
chr2	42213610	rs6713259	3
chr2	42215892	rs1470052	3
chr2	43764218	rs7565517	3
chr2	43841152	rs10179921	3
chr2	48780099	rs7574612	3
chr2	49147884	rs13022022	3
chr2	49190847	rs10178071	3
chr2	57608468	rs17048981	3
chr2	62060808	rs10865330	3
chr2	62315532	rs4672501	3
chr2	64191322	rs1973848	3
chr2	65060762	rs2249105	3
chr2	71638157	rs13387541	3
chr2	72163775	rs975366	3
chr2	72910862	rs10185826	3
chr2	72930266	rs999494	3
chr2	86802550	rs6753271	3
chr2	103294243	rs1430074	3
chr2	103958192	rs2570482	3
chr2	104209873	rs7597477	3
chr2	107938433	rs10206685	3
chr2	109032517	rs260613	3
chr2	109065016	rs6720695	3
chr2	112722037	rs13386452	3
chr2	114818847	rs1519673	3
chr2	116595253	rs1442772	3

chr2	117705327	rs991878	3
chr2	121160197	rs17178580	3
chr2	125813022	rs4972235	3
chr2	134581070	rs6730778	3
chr2	141162649	rs16845527	3
chr2	153030676	rs12618194	3
chr2	153637941	rs10497135	3
chr2	153656437	rs16834011	3
chr2	158749925	rs1040166	3
chr2	159412492	rs3755433	3
chr2	160047248	rs877199	3
chr2	174009726	rs2028889	3
chr2	178118481	rs11692993	3
chr2	179049620	rs16866620	3
chr2	189039948	rs10200215	3
chr2	197131776	rs2579391	3
chr2	198220937	rs4850833	3
chr2	199336882	rs1374357	3
chr2	216792379	rs715049	3
chr2	220447319	rs4535036	3
chr2	221194047	rs1583236	3
chr2	221660200	rs825287	3
chr2	224981812	rs10498177	3
chr2	225133751	rs4674972	3
chr2	234764895	rs7602550	3
chr2	237300432	rs4663712	3
chr2	238150843	rs7605194	3
chr3	2385346	rs1827451	3
chr3	7233238	rs963684	3
chr3	8638015	rs11131141	3
chr3	13144609	rs358374	3
chr3	16553611	rs13081210	3
chr3	16771164	rs1375629	3
chr3	16814451	rs6778524	3
chr3	20647387	rs11714736	3
chr3	24260045	rs9873977	3
chr3	36067502	rs6795407	3
chr3	37623894	rs4678980	3
chr3	54077850	rs750379	3
chr3	60203743	rs1496650	3
chr3	60251982	rs213396	3

chr3	87788660	rs17467509	3
chr3	88476181	rs17025798	3
chr3	88534679	rs6551337	3
chr3	103287801	rs7612427	3
chr3	104817604	rs10446267	3
chr3	106088903	rs767150	3
chr3	106302653	rs7614488	3
chr3	108836409	rs1348994	3
chr3	112411742	rs7609904	3
chr3	113288678	rs11919134	3
chr3	115639308	rs714697	3
chr3	117875664	rs13082166	3
chr3	117921352	rs6805565	3
chr3	127065572	rs4679328	3
chr3	127208573	rs11920916	3
chr3	128458280	rs9850678	3
chr3	130488933	rs1391054	3
chr3	156367068	rs1450110	3
chr3	161433653	rs1450525	3
chr3	169008936	rs13089423	3
chr3	171510899	rs260785	3
chr3	172654139	rs17756228	3
chr3	172995100	rs16846388	3
chr3	178546793	rs1477779	3
chr3	178653566	rs9856750	3
chr3	184575822	rs9823034	3
chr3	193058101	rs992431	3
chr4	6114729	rs11930138	3
chr4	6342477	rs4689410	3
chr4	7153016	rs3915879	3
chr4	7959063	rs4696856	3
chr4	10750119	rs4420973	3
chr4	11742764	rs993835	3
chr4	24998431	rs4697519	3
chr4	37555738	rs2941011	3
chr4	37613989	rs17423188	3
chr4	38005938	rs17579337	3
chr4	38137235	rs13110318	3
chr4	40688268	rs6447325	3
chr4	44370028	rs7674464	3
chr4	44426771	rs17461197	3

chr4	67048906	rs1425663	3
chr4	74688917	rs11097054	3
chr4	75650932	rs9306994	3
chr4	78985869	rs2046105	3
chr4	86831756	rs7690165	3
chr4	94074959	rs7658581	3
chr4	98715064	rs17027976	3
chr4	100214009	rs4450933	3
chr4	104876319	rs4579119	3
chr4	107198265	rs2029635	3
chr4	107200656	rs2866911	3
chr4	108255005	rs1003169	3
chr4	112996626	rs13148888	3
chr4	113055675	rs10007543	3
chr4	122629543	rs4833838	3
chr4	123055484	rs11731720	3
chr4	126441236	rs6836726	3
chr4	129110964	rs1867951	3
chr4	140310104	rs17005729	3
chr4	145448604	rs13109195	3
chr4	145630280	rs13130076	3
chr4	157629089	rs17036576	3
chr4	157816782	rs6812217	3
chr4	159031201	rs1559887	3
chr4	160863879	rs13130758	3
chr4	162263499	rs6536646	3
chr4	168845812	rs1392751	3
chr4	173346075	rs12640749	3
chr4	183283624	rs10520555	3
chr4	188960203	rs7658965	3
chr5	3180894	rs1215665	3
chr5	5318468	rs6885895	3
chr5	10812699	rs2083232	3
chr5	14772136	rs17251715	3
chr5	16903609	rs10520847	3
chr5	17042095	rs7723292	3
chr5	18723359	rs872026	3
chr5	25673542	rs248755	3
chr5	30792702	rs10064804	3
chr5	36631551	rs3776565	3
chr5	38778412	rs9687972	3

chr5	40622847	rs4957326	3
chr5	51482964	rs16878921	3
chr5	51718363	rs16879124	3
chr5	52539565	rs16879891	3
chr5	58669405	rs17346755	3
chr5	66627756	rs4700141	3
chr5	69152867	rs4976175	3
chr5	72820579	rs10069833	3
chr5	77224687	rs12520862	3
chr5	79799594	rs10043986	3
chr5	83097869	rs1011981	3
chr5	84707871	rs10076429	3
chr5	95048084	rs1426099	3
chr5	108123828	rs12652687	3
chr5	109302590	rs1990892	3
chr5	114059429	rs11241260	3
chr5	114098735	rs2721304	3
chr5	123800962	rs6891344	3
chr5	123843555	rs6875311	3
chr5	126460059	rs4836264	3
chr5	126483006	rs4526101	3
chr5	127854224	rs245190	3
chr5	142445759	rs9800206	3
chr5	142636150	rs4912870	3
chr5	145856408	rs10053569	3
chr5	146689099	rs7736604	3
chr5	151338863	rs9324690	3
chr5	166300630	rs13361834	3
chr5	167733427	rs7717495	3
chr5	173545814	rs6897691	3
chr5	173548688	rs10866665	3
chr5	173616963	rs1469068	3
chr6	529104	rs9504229	3
chr6	650071	rs1612811	3
chr6	1307611	rs11242674	3
chr6	1318485	rs12214302	3
chr6	4004445	rs736859	3
chr6	7376770	rs9405336	3
chr6	10242978	rs12526330	3
chr6	11214472	rs1009667	3
chr6	14162718	rs853340	3

chr6	14210232	rs1537147	3
chr6	33005110	rs376892	3
chr6	34073529	rs2499689	3
chr6	34286401	rs6908522	3
chr6	34918659	rs9380470	3
chr6	46869653	rs1928274	3
chr6	53614071	rs10948754	3
chr6	55137239	rs7761785	3
chr6	55430460	rs9370420	3
chr6	55570243	rs4715554	3
chr6	64799196	rs12215142	3
chr6	70892717	rs9294881	3
chr6	71046457	rs9354960	3
chr6	71945154	rs13437473	3
chr6	73807957	rs2882694	3
chr6	84637539	rs13217066	3
chr6	84638970	rs9353206	3
chr6	94370862	rs9354114	3
chr6	107661761	rs4945782	3
chr6	107678919	rs9373966	3
chr6	108740248	rs579702	3
chr6	116204442	rs1204850	3
chr6	116932446	rs2180621	3
chr6	126091807	rs11154343	3
chr6	127957653	rs802734	3
chr6	132504925	rs17192622	3
chr6	133696143	rs6569897	3
chr6	137805141	rs774628	3
chr6	138781229	rs6927344	3
chr6	149018824	rs3798592	3
chr6	149297268	rs531486	3
chr6	151160229	rs752104	3
chr6	153343583	rs17083928	3
chr6	154482550	rs9397724	3
chr6	161520987	rs2223768	3
chr6	166553042	rs9366027	3
chr6	168024396	rs6922131	3
chr6	168962777	rs3818698	3
chr7	861937	rs7809344	3
chr7	6696099	rs3801037	3
chr7	9041142	rs17158609	3

chr7	12435009	rs847982	3
chr7	14204006	rs10486049	3
chr7	15398337	rs13242762	3
chr7	22089981	rs10267816	3
chr7	25525531	rs6956023	3
chr7	31131917	rs4723048	3
chr7	36274469	rs196580	3
chr7	37103082	rs6971267	3
chr7	37151322	rs4602779	3
chr7	47397553	rs12540482	3
chr7	47984157	rs2708906	3
chr7	51738416	rs17152331	3
chr7	71154112	rs2867041	3
chr7	71209285	rs986779	3
chr7	72405691	rs6460722	3
chr7	75899972	rs10231459	3
chr7	78826832	rs519514	3
chr7	81834919	rs10278470	3
chr7	85380033	rs2463468	3
chr7	94571485	rs12539995	3
chr7	106589855	rs10486014	3
chr7	110731707	rs12155444	3
chr7	122085518	rs2215359	3
chr7	128200967	rs6956123	3
chr7	131019300	rs765966	3
chr7	151575378	rs7791529	3
chr7	151833652	rs11764602	3
chr7	151836199	rs1881623	3
chr7	155984534	rs11769678	3
chr8	1429749	rs12674781	3
chr8	11396370	rs10105588	3
chr8	11805376	rs10098874	3
chr8	11824316	rs17756892	3
chr8	11835539	rs3735809	3
chr8	11844424	rs4839	3
chr8	15419198	rs6985514	3
chr8	26354897	rs2046222	3
chr8	32307171	rs10503904	3
chr8	35143179	rs6468305	3
chr8	42486720	rs4368963	3
chr8	51347506	rs16916039	3

chr8	52006570	rs16917100	3
chr8	73479545	rs4237005	3
chr8	84248386	rs10102752	3
chr8	85543966	rs923817	3
chr8	92495786	rs561608	3
chr8	93688173	rs17710303	3
chr8	100440707	rs10107200	3
chr8	106473817	rs2510817	3
chr8	106628879	rs10096751	3
chr8	107074234	rs13255596	3
chr8	112670933	rs6469434	3
chr8	113448450	rs16892653	3
chr8	113540292	rs12679004	3
chr8	114952409	rs1872780	3
chr8	115272939	rs10113167	3
chr8	115631894	rs2737227	3
chr8	117086368	rs7833712	3
chr8	120861105	rs6996149	3
chr8	127460075	rs1447293	3
chr8	129161221	rs4571700	3
chr8	136090192	rs930647	3
chr8	136794531	rs2613837	3
chr8	138295718	rs10109439	3
chr8	141212346	rs3739238	3
chr9	4172087	rs10974362	3
chr9	4203214	rs10974390	3
chr9	7607425	rs10815647	3
chr9	7735283	rs17629093	3
chr9	8423755	rs10977081	3
chr9	9032872	rs172862	3
chr9	12698363	rs2075508	3
chr9	14763857	rs7039708	3
chr9	15330633	rs12554359	3
chr9	17008139	rs263628	3
chr9	17165700	rs2815175	3
chr9	21604068	rs16938481	3
chr9	26028354	rs877755	3
chr9	32563916	rs10971025	3
chr9	32592505	rs10971038	3
chr9	34942076	rs10283808	3
chr9	35063792	rs623318	3

chr9	35088145	rs527459	3
chr9	35427327	rs10972462	3
chr9	37438529	rs2840246	3
chr9	76343683	rs10781357	3
chr9	81638512	rs2378591	3
chr9	84345145	rs10780664	3
chr9	85302543	rs1983944	3
chr9	86271659	rs10512174	3
chr9	98149140	rs2795491	3
chr9	98845743	rs10987952	3
chr9	101000235	rs10989350	3
chr9	110258636	rs3758192	3
chr9	110557052	rs9299186	3
chr9	117225120	rs1016731	3
chr9	132401743	rs3739915	3
chr9	133331135	rs2491	3
chr9	134932694	rs2382712	3
chr9	135050008	rs616656	3
chr10	1126899	rs7894626	3
chr10	1450317	rs10903450	3
chr10	12894781	rs2399915	3
chr10	12932824	rs1251015	3
chr10	13563368	rs4279918	3
chr10	13608629	rs2478120	3
chr10	15103949	rs10242	3
chr10	18326066	rs11013624	3
chr10	18523505	rs10828834	3
chr10	33895135	rs1757476	3
chr10	34643300	rs2031335	3
chr10	43952572	rs1254860	3
chr10	50449629	rs10826013	3
chr10	54154011	rs11004102	3
chr10	54444531	rs7476422	3
chr10	56798193	rs16909484	3
chr10	58137824	rs12359064	3
chr10	58980556	rs204940	3
chr10	69239736	rs7085830	3
chr10	78734425	rs7917285	3
chr10	87885716	rs1234220	3
chr10	88787747	rs11202855	3
chr10	104355789	rs12769490	3

chr10	105562272	rs7076193	3
chr10	111072467	rs17128356	3
chr10	113592246	rs11196389	3
chr10	120804434	rs10886770	3
chr10	124089909	rs4397783	3
chr10	129316250	rs2622441	3
chr10	129862234	rs10734091	3
chr10	131693012	rs10872828	3
chr11	4673078	rs11033277	3
chr11	6920508	rs12288515	3
chr11	8068452	rs7114039	3
chr11	12872400	rs11022522	3
chr11	12918649	rs747113	3
chr11	15782247	rs11600427	3
chr11	20952310	rs1987307	3
chr11	22804013	rs1544570	3
chr11	24647091	rs12576803	3
chr11	24756565	rs2716548	3
chr11	24806695	rs12800427	3
chr11	28143314	rs2128958	3
chr11	30760793	rs621897	3
chr11	38151859	rs2127541	3
chr11	39844936	rs11035522	3
chr11	59449573	rs17153737	3
chr11	61902474	rs3815045	3
chr11	66495135	rs2511224	3
chr11	67387739	rs10896171	3
chr11	71206597	rs4245413	3
chr11	78793722	rs627195	3
chr11	83845024	rs7951794	3
chr11	86506719	rs638492	3
chr11	97888820	rs6589075	3
chr11	98709456	rs1541859	3
chr11	98827348	rs4556507	3
chr11	99217316	rs7103056	3
chr11	109519077	rs1837397	3
chr11	120148035	rs675647	3
chr11	128934894	rs2155548	3
chr11	131537869	rs425354	3
chr12	2841338	rs4766000	3
chr12	3951793	rs7978351	3

chr12	7855536	rs2889504	3
chr12	12212391	rs17302049	3
chr12	21846929	rs829080	3
chr12	25808109	rs2880274	3
chr12	25823617	rs1462705	3
chr12	27851953	rs258394	3
chr12	30076673	rs7964334	3
chr12	30232799	rs3862404	3
chr12	45959496	rs1012642	3
chr12	59671419	rs4760299	3
chr12	62780955	rs337515	3
chr12	66610621	rs10506491	3
chr12	66730981	rs1480027	3
chr12	69987575	rs1689467	3
chr12	71217099	rs3851621	3
chr12	84606143	rs1471239	3
chr12	84750310	rs4761090	3
chr12	84936717	rs12316387	3
chr12	85826318	rs7313153	3
chr12	92351137	rs7137475	3
chr12	101528756	rs7312125	3
chr12	106229679	rs1215761	3
chr12	113425138	rs1465542	3
chr12	115818096	rs7964855	3
chr12	116416318	rs1732325	3
chr12	118402652	rs12316703	3
chr12	123635436	rs11572920	3
chr12	126028552	rs1468560	3
chr12	126765026	rs1194047	3
chr12	131855412	rs4964933	3
chr13	21052734	rs7330095	3
chr13	23392006	rs1008812	3
chr13	23459063	rs17388607	3
chr13	26869673	rs7338990	3
chr13	27016263	rs9512492	3
chr13	27819535	rs1616483	3
chr13	30795572	rs4769889	3
chr13	34289583	rs9540388	3
chr13	38144398	rs9548217	3
chr13	42264468	rs9315903	3
chr13	51318354	rs9535643	3

chr13	53262823	rs9596772	3
chr13	70973309	rs17087794	3
chr13	75512027	rs17064600	3
chr13	75669920	rs2147006	3
chr13	82962033	rs2255386	3
chr13	84520864	rs17374187	3
chr13	85059443	rs1500682	3
chr13	97566332	rs9556739	3
chr13	104221522	rs10161771	3
chr13	105154603	rs3007243	3
chr13	107830140	rs9555414	3
chr13	110521312	rs10220027	3
chr13	112198359	rs4907674	3
chr14	21302522	rs17792599	3
chr14	21534470	rs1263812	3
chr14	22600948	rs970348	3
chr14	25915740	rs1956613	3
chr14	29105264	rs10483345	3
chr14	29488414	rs1191571	3
chr14	32321351	rs17098722	3
chr14	39546772	rs12437323	3
chr14	42000631	rs8019977	3
chr14	43404779	rs12432613	3
chr14	47162695	rs7153058	3
chr14	47277083	rs1028829	3
chr14	52905612	rs8008270	3
chr14	59134583	rs17833716	3
chr14	78315652	rs17107326	3
chr14	79521446	rs10130369	3
chr14	79638233	rs17109649	3
chr14	79705751	rs10400751	3
chr14	82158154	rs799040	3
chr14	82230310	rs17588820	3
chr14	87793014	rs1152368	3
chr14	87943183	rs2285008	3
chr14	90368516	rs1958349	3
chr14	91369893	rs1285817	3
chr14	97123227	rs4905580	3
chr14	103216241	rs2771369	3
chr15	23003206	rs2289815	3
chr15	29990316	rs7164994	3

chr15	36588417	rs6495850	3
chr15	50988066	rs10519288	3
chr15	51154719	rs4775920	3
chr15	53775921	rs4774675	3
chr15	55009194	rs12594729	3
chr15	58341621	rs261316	3
chr15	58720123	rs4775090	3
chr15	61174799	rs4774388	3
chr15	69979558	rs2415071	3
chr15	70088148	rs2651499	3
chr15	79471205	rs1046582	3
chr15	81478924	rs6495589	3
chr15	86303996	rs7167360	3
chr15	86612325	rs12595578	3
chr15	90465304	rs12912995	3
chr15	92229538	rs11632145	3
chr16	7117353	rs8046633	3
chr16	7483305	rs17143804	3
chr16	8446033	rs12930293	3
chr16	10251811	rs3852749	3
chr16	10877617	rs12928665	3
chr16	12432959	rs1019575	3
chr16	19341759	rs2097266	3
chr16	22820840	rs4783491	3
chr16	25905266	rs13380588	3
chr16	27811072	rs8059523	3
chr16	28043533	rs205393	3
chr16	30070046	rs2071390	3
chr16	58913874	rs9925672	3
chr16	59688845	rs8051651	3
chr16	65189269	rs8059958	3
chr16	70690098	rs11648765	3
chr16	75090096	rs4447456	3
chr16	77837600	rs9938116	3
chr16	82315639	rs2906801	3
chr16	82504559	rs16957560	3
chr16	83453711	rs8054845	3
chr16	83514921	rs4476163	3
chr16	85262625	rs8053353	3
chr16	86370598	rs1532167	3
chr17	3724918	rs220461	3

chr17	9209786	rs7224773	3
chr17	12299021	rs2529737	3
chr17	13700792	rs9910556	3
chr17	16901209	rs11657586	3
chr17	35750409	rs11650416	3
chr17	41318541	rs9916485	3
chr17	45118646	rs3744761	3
chr17	45705974	rs1526123	3
chr17	50276870	rs9891711	3
chr17	57933599	rs8074980	3
chr17	77088933	rs8082404	3
chr18	3624533	rs2290871	3
chr18	5417184	rs17466502	3
chr18	8972529	rs1893143	3
chr18	11722049	rs12604626	3
chr18	25955956	rs8091949	3
chr18	26899996	rs1030198	3
chr18	28463039	rs12966353	3
chr18	31962241	rs4799610	3
chr18	39470720	rs1681045	3
chr18	40919993	rs12957072	3
chr18	51337688	rs2445473	3
chr18	69917640	rs17081819	3
chr18	70661305	rs966667	3
chr18	76035205	rs1857977	3
chr18	76574371	rs12456298	3
chr18	77168272	rs1789138	3
chr19	6452294	rs173229	3
chr19	6927985	rs3895916	3
chr19	7814440	rs12984528	3
chr19	10271861	rs5030390	3
chr19	32908438	rs892024	3
chr19	35521182	rs2106446	3
chr19	43942698	rs10416702	3
chr19	53266608	rs16984664	3
chr19	55051034	rs6509916	3
chr20	2263375	rs1575124	3
chr20	5298347	rs6053268	3
chr20	5967581	rs16991615	3
chr20	10083881	rs551927	3
chr20	12254154	rs6134489	3

chr20	12502954	rs10460629	3
chr20	15656879	rs459928	3
chr20	20823221	rs6106294	3
chr20	23335864	rs6036381	3
chr20	34977952	rs11906160	3
chr20	39085333	rs6016010	3
chr20	40176426	rs6028939	3
chr20	40255548	rs6129532	3
chr20	40264672	rs6071993	3
chr20	42277858	rs6102757	3
chr20	42315056	rs6030128	3
chr20	42399347	rs10485693	3
chr20	42485579	rs6093656	3
chr20	48249979	rs170536	3
chr21	18052633	rs2260211	3
chr21	30314691	rs7277936	3
chr21	37149542	rs2835630	3
chr21	37830091	rs3787840	3
chr22	17843396	rs9618142	3
chr22	23064885	rs13054331	3
chr22	25113063	rs2294371	3
chr22	25212329	rs12169716	3
chr22	25542058	rs542162	3
chr22	27625829	rs5762284	3
chr22	30595137	rs7285863	3
chr22	33651807	rs5754630	3
chr22	48921802	rs761639	3
chr6	31333881	rs9378199	3
chr1	39809399	rs7531010	4
chr1	86040072	rs12143304	4
chr1	87213097	rs4506441	4
chr1	115628084	rs4839463	4
chr1	180622101	rs3908502	4
chr1	186958237	rs6683363	4
chr1	204475907	rs1553920	4
chr2	85457087	rs6716108	4
chr2	102793226	rs7604365	4
chr2	136716572	rs1427611	4
chr2	222125296	rs16863422	4
chr2	224160403	rs1453672	4
chr3	21678882	rs10510515	4

chr3	42180055	rs6775303	4
chr3	60032600	rs17061880	4
chr3	66759247	rs9870722	4
chr3	119612055	rs2688647	4
chr3	158819321	rs2615055	4
chr4	947730	rs2290402	4
chr4	16869692	rs13104451	4
chr4	28096463	rs6824779	4
chr4	72524658	rs12511787	4
chr4	88855095	rs11735482	4
chr4	134530724	rs4293833	4
chr4	169657851	rs17627811	4
chr4	188923347	rs7677253	4
chr5	84722520	rs10067358	4
chr5	95824118	rs3756705	4
chr5	133425999	rs1011166	4
chr5	151083077	rs13168551	4
chr5	151093543	rs6579838	4
chr5	170583376	rs10462997	4
chr6	27457406	rs1883216	4
chr6	31025756	rs2523898	4
chr6	32756140	rs7756516	4
chr6	106180035	rs9398071	4
chr7	12246783	rs6945902	4
chr7	153950146	rs11974308	4
chr7	155168662	rs6971088	4
chr8	2609219	rs4279621	4
chr8	11830639	rs4841600	4
chr8	19942130	rs1534649	4
chr8	73615761	rs949493	4
chr9	4657040	rs2146423	4
chr9	9763352	rs1174581	4
chr9	27373417	rs17768620	4
chr9	83753745	rs7047907	4
chr9	107103555	rs7030936	4
chr10	27380895	rs606448	4
chr10	27526736	rs2642273	4
chr10	112437884	rs7070744	4
chr10	130032635	rs2477970	4
chr11	22195849	rs4581442	4
chr11	35139529	rs3751031	4

chr11	35147371	rs7126359	4
chr12	7697835	rs7132821	4
chr12	57149789	rs10876966	4
chr12	80592093	rs1528287	4
chr12	101619104	rs825090	4
chr14	21099330	rs1952512	4
chr15	33884469	rs1477436	4
chr15	63503638	rs289818	4
chr15	97902567	rs6496267	4
chr16	77741562	rs11648791	4
chr16	82588535	rs4783229	4
chr17	31737201	rs16966855	4
chr17	44360314	rs708384	4
chr17	46951928	rs3809854	4
chr17	57673751	rs2240601	4
chr17	70319165	rs11868369	4
chr18	3954314	rs11081080	4
chr19	3201697	rs311625	4
chr19	45417538	rs3212964	4
chr20	755214	rs6085691	4
chr20	44031646	rs2179593	4
chr20	44784557	rs1080026	4
chr21	32866016	rs743328	4
chr21	41402614	rs466092	4
chr22	27131043	rs1003704	4
chr22	31140147	rs2232176	4
chr1	5104364	rs2169090	5
chr1	7388196	rs845232	5
chr1	22041849	rs2501279	5
chr1	80018940	rs1340674	5
chr1	205237367	rs1172161	5
chr1	229029741	rs484947	5
chr1	233540587	rs3845310	5
chr2	8769019	rs718763	5
chr2	47523088	rs6744097	5
chr2	47797737	rs3136329	5
chr2	103965555	rs2375937	5
chr2	104097673	rs2889276	5
chr2	108058237	rs1486176	5
chr2	124155940	rs960537	5
chr2	153393709	rs10931598	5

chr3	24719697	rs4411831	5
chr3	116421727	rs13316464	5
chr3	151814106	rs2293004	5
chr4	34586491	rs4859325	5
chr4	185309122	rs1288569	5
chr5	36482938	rs2455253	5
chr5	36523504	rs2562534	5
chr5	62683162	rs4700505	5
chr5	126550379	rs1038381	5
chr6	25874195	rs13198474	5
chr6	31379280	rs9266638	5
chr6	31386405	rs6932730	5
chr6	54845113	rs1503133	5
chr6	117361609	rs1407180	5
chr6	154488268	rs12202842	5
chr6	161518615	rs11965303	5
chr7	12057041	rs10261234	5
chr7	12058075	rs10251790	5
chr7	12124474	rs1003433	5
chr7	28894886	rs4719959	5
chr7	127524904	rs6467136	5
chr8	8768260	rs656070	5
chr8	97167985	rs6990629	5
chr8	136803442	rs2649127	5
chr9	4833437	rs458552	5
chr9	7639776	rs12378544	5
chr9	70374916	rs4745008	5
chr10	20747145	rs11012310	5
chr10	29235521	rs581090	5
chr10	49161350	rs2725180	5
chr10	80343474	rs12411833	5
chr11	25829847	rs1596071	5
chr11	27209391	rs1442931	5
chr11	44763736	rs715059	5
chr12	23863248	rs10505909	5
chr12	47718594	rs929270	5
chr12	123585667	rs786448	5
chr12	129336952	rs10847834	5
chr13	27356763	rs6491207	5
chr14	23646900	rs17184233	5
chr14	64787747	rs2269303	5

chr14	84977188	rs7147908	5
chr14	87807501	rs2401711	5
chr14	87824961	rs2167239	5
chr15	53582394	rs10518730	5
chr16	27602444	rs11074865	5
chr16	86716941	rs9888784	5
chr17	45707411	rs1617406	5
chr18	59479122	rs668432	5
chr19	43311416	rs8113515	5
chr20	20048820	rs6046570	5
chr20	39129112	rs2868503	5
chr20	42444015	rs3890324	5
chr21	33853831	rs2249221	5
chr21	35061666	rs928282	5
chr22	25072863	rs17668150	5
chr22	47964736	rs16998000	5
