# Supplementary material for: Polygenic associations with clinical and neuropathological trait heterogeneity across TDP-43 proteinopathies
Source: PLoS One. 2025 Dec 30;20(12):e0338398. doi: 10.1371/journal.pone.0338398 (PMC12752993; doi:10.1371/journal.pone.0338398)
Supplement: S2 File — Pathways that met FDR-adjusted p < .05 are listed for each cluster. (PDF) [file pone.0338398.s009.pdf]

Module	GO BP Term Name	Fold Enrichment	p-value
C1 ALS	positive regulation of retrograde transport endosome to Golgi	54.40721649	0.01334544
C1 ALS	response to stem cell factor	40.80541237	0.0015537
C1 ALS	Kit signaling pathway	40.80541237	0.0015537
C1 ALS	cellular response to stem cell factor stimulus	40.80541237	0.0015537
C1 ALS	regulation of synaptic activity	36.27147766	0.0309056
C1 ALS	lateral motor column neuron migration	36.27147766	0.0309056
C1 ALS	spinal cord ventral commissure morphogenesis	36.27147766	0.0309056
C1 ALS	clathrin-dependent synaptic vesicle endocytosis	36.27147766	0.0309056
C1 ALS	spinal cord motor neuron migration	27.20360825	0.04903205
C1 ALS	radial glia guided migration of Purkinje cell	27.20360825	0.04903205
C1 ALS	epithelial cell proliferation involved in renal tubule morphogenesis	27.20360825	0.04903205
C1 ALS	regulation of blastocyst development	27.20360825	0.04903205
C1 ALS	anterior/posterior axon guidance	23.3173785	0.00913185
C1 ALS	negative regulation of chemokine-mediated signaling pathway	23.3173785	0.00913185
C1 ALS	trans-synaptic signaling by trans-synaptic complex	20.40270619	0.01288859
C1 ALS	regulation of glucokinase activity	16.32216495	0.02272135
C1 ALS	regulation of chemokine-mediated signaling pathway	16.32216495	0.02272135
C1 ALS	negative regulation of collateral sprouting	14.83833177	0.02929983
C1 ALS	regulation of hexokinase activity	14.83833177	0.02929983
C1 ALS	suckling behavior	13.60180412	0.03427898
C1 ALS	negative regulation of platelet aggregation	13.60180412	0.03427898
C1 ALS	regulation of NMDA receptor activity	11.82765576	0.00283115
C1 ALS	motor neuron migration	11.65868925	0.04903205
C1 ALS	negative regulation of homotypic cell-cell adhesion	11.65868925	0.04903205
C1 ALS	retinal ganglion cell axon guidance	10.36327933	0.0188547
C1 ALS	negative regulation of receptor signaling pathway via JAK-STAT	9.892221181	0.02206827
C1 ALS	collateral sprouting	9.715574374	0.00690813
C1 ALS	positive regulation of long-term synaptic potentiation	9.067869416	0.02907847
C1 ALS	regulation of platelet aggregation	8.775357499	0.01020247
C1 ALS	negative regulation of receptor signaling pathway via STAT	8.705154639	0.03141479
C1 ALS	regulation of dendrite extension	8.705154639	0.03141479
C1 ALS	dendrite extension	7.772459499	0.0159531

C1 ALS	startle response	7.504443654	0.04779814
C1 ALS	central nervous system projection neuron axonogenesis	7.504443654	0.04779814
C1 ALS	regulation of homotypic cell-cell adhesion	7.158844276	0.02189422
C1 ALS	neuron recognition	6.662108142	0.01119044
C1 ALS	negative regulation of axonogenesis	6.496384059	0.00191083
C1 ALS	modulation of excitatory postsynaptic potential	6.045246277	0.0381649
C1 ALS	regulation of dendrite development	5.6460319	0.00032491
C1 ALS	vitamin transport	5.551756785	0.04962092
C1 ALS	regulation of dendrite morphogenesis	5.519572688	0.01133676
C1 ALS	regulation of synapse assembly	5.204168534	0.00065435
C1 ALS	positive regulation of synapse assembly	5.100676546	0.03297457
C1 ALS	axon guidance	5.025566182	4.47E-08
C1 ALS	regulation of neurotransmitter receptor activity	5.0222046	0.03467138
C1 ALS	neuron projection guidance	5.005463918	4.58E-08
C1 ALS	regulation of blood coagulation	4.6634757	0.04703214
C1 ALS	developmental growth involved in morphogenesis	4.477960205	3.30E-06
C1 ALS	dendrite development	4.477960205	3.30E-06
C1 ALS	axonogenesis	4.257956074	6.80E-11
C1 ALS	actin cytoskeleton reorganization	4.257956074	0.01133676
C1 ALS	regulation of axonogenesis	4.106205018	0.00237165
C1 ALS	neuron projection morphogenesis	4.10146709	4.35E-14
C1 ALS	cell part morphogenesis	4.094320199	2.32E-14
C1 ALS	plasma membrane bounded cell projection morphogenesis	4.084625863	4.35E-14
C1 ALS	positive regulation of cell junction assembly	4.067829271	0.02662524
C1 ALS	cell projection morphogenesis	4.054189009	4.35E-14
C1 ALS	synapse assembly	4.040139839	0.00040383
C1 ALS	developmental cell growth	4.03896367	0.00011169
C1 ALS	smooth muscle contraction	3.956888472	0.0308857
C1 ALS	axon development	3.95494501	2.65E-10
C1 ALS	positive regulation of neuron projection development	3.911629944	0.0062706
C1 ALS	cell morphogenesis involved in neuron differentiation	3.873056089	1.88E-11
C1 ALS	negative regulation of neuron projection development	3.858667837	0.01164265
C1 ALS	dendrite morphogenesis	3.778278923	0.01334544

C1 ALS	cellular component morphogenesis	3.690941823	5.80E-14
C1 ALS	neuron migration	3.667902236	0.00584023
C1 ALS	sensory perception of sound	3.627147766	0.01057726
C1 ALS	cell morphogenesis involved in differentiation	3.534003488	1.69E-10
C1 ALS	regulation of neuron projection development	3.514051845	7.82E-07
C1 ALS	learning	3.510143	0.02147228
C1 ALS	negative regulation of cell projection organization	3.436245252	0.00959581
C1 ALS	cell morphogenesis	3.393497143	4.35E-14
C1 ALS	neuron projection extension	3.362243716	0.01748972
C1 ALS	neuron projection development	3.352363845	5.80E-14
C1 ALS	negative regulation of neurogenesis	3.308546949	0.04506652
C1 ALS	adult behavior	3.308546949	0.04506652
C1 ALS	regulation of cell junction assembly	3.305111282	0.00831417
C1 ALS	response to mechanical stimulus	3.229652121	0.00968554
C1 ALS	regulation of plasma membrane bounded cell projection organization	3.220364839	4.47E-08
C1 ALS	homophilic cell adhesion via plasma membrane adhesion molecules	3.2004245	0.03467138
C1 ALS	sensory perception of mechanical stimulus	3.183400965	0.02479919
C1 ALS	regulation of cell projection organization	3.151637541	7.20E-08
C1 ALS	neuron development	3.138877875	5.80E-14
C1 ALS	regulation of synaptic plasticity	3.12385932	0.018623
C1 ALS	regulation of synapse organization	2.94705756	0.01891824
C1 ALS	positive regulation of cell projection organization	2.928433749	0.00199725
C1 ALS	regulation of cell morphogenesis	2.918394755	0.01412691
C1 ALS	regulation of synapse structure or activity	2.875178107	0.02272135
C1 ALS	cell-cell adhesion via plasma-membrane adhesion molecules	2.86353771	0.01133676
C1 ALS	protein autophosphorylation	2.826348909	0.03467138
C1 ALS	peptidyl-tyrosine phosphorylation	2.778863208	0.00340274
C1 ALS	glycoprotein biosynthetic process	2.772342242	0.01065319
C1 ALS	peptidyl-tyrosine modification	2.764002977	0.00361527
C1 ALS	chemotaxis	2.759161534	2.64E-05
C1 ALS	synapse organization	2.754795772	0.0006328
C1 ALS	taxis	2.750443804	2.77E-05
C1 ALS	neuron differentiation	2.729973407	2.67E-12

C1 ALS	generation of neurons	2.669171239	3.05E-12
C1 ALS	plasma membrane bounded cell projection organization	2.658333053	1.76E-12
C1 ALS	trans-synaptic signaling	2.652262201	8.90E-06
C1 ALS	synaptic signaling	2.647214119	6.32E-06
C1 ALS	anterograde trans-synaptic signaling	2.60460079	1.92E-05
C1 ALS	chemical synaptic transmission	2.60460079	1.92E-05
C1 ALS	regulation of anatomical structure morphogenesis	2.59670806	2.23E-06
C1 ALS	cell projection organization	2.594113946	5.01E-12
C1 ALS	cell-cell adhesion	2.577183939	8.71E-07
C1 ALS	cell junction assembly	2.573314294	0.00414167
C1 ALS	modulation of chemical synaptic transmission	2.553808121	0.00247856
C1 ALS	learning or memory	2.553407272	0.04903205
C1 ALS	neurogenesis	2.552831272	1.76E-12
C1 ALS	regulation of trans-synaptic signaling	2.548606883	0.00249839
C1 ALS	regulation of developmental growth	2.526650921	0.0309342
C1 ALS	negative regulation of cell motility	2.513864789	0.04187775
C1 ALS	positive regulation of DNA metabolic process	2.497380429	0.04404055
C1 ALS	developmental growth	2.49193358	0.00036708
C1 ALS	glycoprotein metabolic process	2.49193358	0.01484144
C1 ALS	morphogenesis of an epithelium	2.477748	0.00260971
C1 ALS	epithelial tube morphogenesis	2.40739896	0.04395455
C1 ALS	cell growth	2.37491818	0.00833488
C1 ALS	small GTPase mediated signal transduction	2.365531152	0.00856626
C1 ALS	regulation of nervous system development	2.360399848	0.01454589
C1 ALS	tissue morphogenesis	2.309740323	0.00286387
C1 ALS	cell adhesion	2.275857422	7.20E-08
C1 ALS	actin filament-based process	2.264192608	0.00063834
C1 ALS	positive regulation of MAPK cascade	2.252886811	0.02272135
C1 ALS	actin cytoskeleton organization	2.232854302	0.00234209
C1 ALS	wound healing	2.210676968	0.04158023
C1 ALS	cell junction organization	2.205697966	0.00263566
C1 ALS	nervous system development	2.200213309	1.76E-12
C1 ALS	response to wounding	2.172510381	0.01688988

C1 ALS	locomotion	2.1523734	9.49E-06
C1 ALS	positive regulation of cellular component organization	2.139336484	0.00014508
C1 ALS	regulation of MAPK cascade	2.105041114	0.01281515
C1 ALS	behavior	2.095809572	0.0165412
C1 ALS	sensory organ development	2.082139733	0.02662524
C1 ALS	cell surface receptor signaling pathway involved in cell-cell signaling	2.067286292	0.03307901
C1 ALS	anatomical structure morphogenesis	2.066350774	5.70E-11
C1 ALS	brain development	2.045384079	0.00849433
C1 ALS	growth	2.032286635	0.00315518
C1 ALS	central nervous system development	1.992938333	0.00172601
C1 ALS	cell-cell signaling	1.988943056	1.55E-05
C1 ALS	cell development	1.969135595	1.91E-09
C1 ALS	regulation of kinase activity	1.956552737	0.02996933
C1 ALS	regulation of cell migration	1.936826477	0.01028536
C1 ALS	regulation of cell motility	1.927381151	0.00808347
C1 ALS	head development	1.924783602	0.01843686
C1 ALS	MAPK cascade	1.902843064	0.03297457
C1 ALS	regulation of locomotion	1.896088861	0.00856626
C1 ALS	system development	1.883169269	1.76E-12
C1 ALS	cell migration	1.843699695	0.00118643
C1 ALS	tube morphogenesis	1.819849225	0.04091279
C1 ALS	regulation of cellular component biogenesis	1.793028762	0.03220714
C1 ALS	negative regulation of multicellular organismal process	1.764558373	0.02390932
C1 ALS	regulation of developmental process	1.731736544	6.04E-05
C1 ALS	regulation of cellular component organization	1.723705478	8.53E-05
C1 ALS	cell motility	1.723161109	0.00315452
C1 ALS	cellular developmental process	1.715881367	1.74E-09
C1 ALS	intracellular signal transduction	1.712329032	4.18E-05
C1 ALS	cell differentiation	1.701013021	4.77E-09
C1 ALS	peptidyl-amino acid modification	1.700225515	0.0309342
C1 ALS	multicellular organism development	1.68197492	1.73E-09
C1 ALS	response to external stimulus	1.669738713	4.86E-05
C1 ALS	regulation of intracellular signal transduction	1.668739858	0.00766474

C1 ALS	negative regulation of signal transduction	1.651337242	0.04506652
C1 ALS	negative regulation of response to stimulus	1.616055935	0.02272135
C1 ALS	anatomical structure development	1.614868197	6.55E-11
C1 ALS	animal organ development	1.60021225	0.00016759
C1 ALS	developmental process	1.584676209	1.88E-11
C1 ALS	protein phosphorylation	1.5826275	0.04158023
C1 ALS	cytoskeleton organization	1.582232337	0.04521999
C1 ALS	regulation of signaling	1.578175925	0.00011669
C1 ALS	regulation of cell communication	1.57356542	0.00013103
C1 ALS	regulation of signal transduction	1.558115424	0.00099043
C1 ALS	phosphorylation	1.552752122	0.03265517
C1 ALS	regulation of biological quality	1.52354891	0.00258029
C1 ALS	protein modification process	1.48916628	0.00118643
C1 ALS	phosphorus metabolic process	1.463608765	0.01538412
C1 ALS	phosphate-containing compound metabolic process	1.45716034	0.01843686
C1 ALS	system process	1.453718881	0.04672498
C1 ALS	regulation of response to stimulus	1.450678337	0.00144686
C1 ALS	macromolecule modification	1.445191688	0.00247856
C1 ALS	cell communication	1.441741806	8.35E-07
C1 ALS	signaling	1.441250768	1.27E-06
C1 ALS	regulation of molecular function	1.430661532	0.03984122
C1 ALS	multicellular organismal process	1.417295297	7.20E-08
C1 ALS	signal transduction	1.4066168	4.53E-05
C1 ALS	regulation of multicellular organismal process	1.404754348	0.03114313
C1 ALS	negative regulation of cellular process	1.385077466	0.00248361
C1 ALS	protein metabolic process	1.367918638	0.00125753
C1 ALS	positive regulation of cellular process	1.356546805	0.00144686
C1 ALS	response to stimulus	1.354039305	1.25E-07
C1 ALS	cellular response to stimulus	1.30936128	0.00033926
C1 ALS	organonitrogen compound metabolic process	1.309287154	0.00247856
C1 ALS	positive regulation of biological process	1.300707283	0.0038885
C1 ALS	negative regulation of biological process	1.256538884	0.0318867
C1 ALS	regulation of cellular process	1.239199295	2.78E-05

C1 ALS	biological regulation	1.194417987	4.55E-05
C1 ALS	regulation of biological process	1.190157861	0.00019209
C2 FTLD-TDP	negative regulation of interleukin-18 production	44.53586498	0.03017553
C2 FTLD-TDP	actin filament network formation	22.26793249	0.01361393
C2 FTLD-TDP	lipid phosphorylation	16.70094937	0.00527982
C2 FTLD-TDP	positive regulation of lamellipodium assembly	11.13396624	0.0052586
C2 FTLD-TDP	nucleus localization	8.907172996	0.04300969
C2 FTLD-TDP	positive regulation of lamellipodium organization	8.789973351	0.01288875
C2 FTLD-TDP	neuron recognition	8.180056833	0.00551352
C2 FTLD-TDP	regulation of lamellipodium assembly	7.952833032	0.01965879
C2 FTLD-TDP	lamellipodium assembly	5.416524119	0.0373547
C2 FTLD-TDP	regulation of postsynapse organization	5.239513527	0.00849898
C2 FTLD-TDP	transmission of nerve impulse	5.138753651	0.04699514
C2 FTLD-TDP	synaptic transmission glutamatergic	5.08981314	0.00978467
C2 FTLD-TDP	dendritic spine development	5.02824282	0.0236587
C2 FTLD-TDP	dendrite morphogenesis	4.639152602	0.00463132
C2 FTLD-TDP	actin filament bundle assembly	4.621646366	0.00237844
C2 FTLD-TDP	actin filament bundle organization	4.536060322	0.00272757
C2 FTLD-TDP	postsynapse organization	4.197097223	0.00265348
C2 FTLD-TDP	adult behavior	4.062393089	0.01979985
C2 FTLD-TDP	synapse assembly	3.968542424	0.00413602
C2 FTLD-TDP	axon guidance	3.756036805	0.00217394
C2 FTLD-TDP	neuron projection guidance	3.741012658	0.00223027
C2 FTLD-TDP	extracellular matrix organization	3.711322082	0.00028331
C2 FTLD-TDP	extracellular structure organization	3.699902629	0.00028746
C2 FTLD-TDP	external encapsulating structure organization	3.677273255	0.00030472
C2 FTLD-TDP	synapse organization	3.664343321	4.65E-06
C2 FTLD-TDP	regulation of synapse organization	3.61853903	0.00503494
C2 FTLD-TDP	establishment or maintenance of cell polarity	3.573865708	0.00537865
C2 FTLD-TDP	dendrite development	3.573865708	0.00537865
C2 FTLD-TDP	regulation of synapse structure or activity	3.53028198	0.00559981
C2 FTLD-TDP	regulation of plasma membrane bounded cell projection assembly	3.373929165	0.03725509
C2 FTLD-TDP	regulation of cell projection assembly	3.340189873	0.03945445

C2 FTLD-TDP actin filament organization	3.244334535	0.000211
C2 FTLD-TDP cell morphogenesis involved in neuron differentiation	3.057122935	5.47E-05
C2 FTLD-TDP regulation of cell projection organization	3.055051713	1.64E-05
C2 FTLD-TDP positive regulation of cell projection organization	3.027934163	0.00559981
C2 FTLD-TDP regulation of plasma membrane bounded cell projection organization	3.01761702	3.06E-05
C2 FTLD-TDP plasma membrane bounded cell projection morphogenesis	3.009180066	2.16E-05
C2 FTLD-TDP cell part morphogenesis	2.996986572	1.59E-05
C2 FTLD-TDP cell projection morphogenesis	2.986756966	2.41E-05
C2 FTLD-TDP neuron projection morphogenesis	2.980477118	3.62E-05
C2 FTLD-TDP cell junction organization	2.979088265	7.58E-06
C2 FTLD-TDP regulation of neuron projection development	2.975670266	0.00149714
C2 FTLD-TDP axon development	2.887393997	0.00100056
C2 FTLD-TDP modulation of chemical synaptic transmission	2.863019892	0.00158237
C2 FTLD-TDP regulation of trans-synaptic signaling	2.857188894	0.00159895
C2 FTLD-TDP neuron projection development	2.834100499	5.26E-07
C2 FTLD-TDP cell morphogenesis involved in differentiation	2.825538262	0.00013207
C2 FTLD-TDP regulation of actin filament-based process	2.783491561	0.00866902
C2 FTLD-TDP axonogenesis	2.759287287	0.00503494
C2 FTLD-TDP neuron development	2.744561609	1.96E-07
C2 FTLD-TDP actin cytoskeleton organization	2.741605915	0.00010608
C2 FTLD-TDP regulation of actin cytoskeleton organization	2.737860552	0.0214888
C2 FTLD-TDP cell junction assembly	2.70826206	0.00804313
C2 FTLD-TDP cell adhesion	2.707081989	2.06E-09
C2 FTLD-TDP cellular component morphogenesis	2.685579798	7.19E-05
C2 FTLD-TDP actin filament-based process	2.616550207	0.00011805
C2 FTLD-TDP plasma membrane bounded cell projection organization	2.567702965	2.48E-08
C2 FTLD-TDP cell projection organization	2.548142306	2.48E-08
C2 FTLD-TDP cell morphogenesis	2.527342031	4.31E-05
C2 FTLD-TDP anterograde trans-synaptic signaling	2.487375438	0.00100056
C2 FTLD-TDP chemical synaptic transmission	2.487375438	0.00100056
C2 FTLD-TDP generation of neurons	2.469226385	2.20E-07
C2 FTLD-TDP trans-synaptic signaling	2.464435216	0.00115422
C2 FTLD-TDP chemotaxis	2.435003711	0.00550724

C2 FTLD-TDP taxis	2.427310177	0.00559981
C2 FTLD-TDP neuron differentiation	2.407769379	1.66E-06
C2 FTLD-TDP synaptic signaling	2.395014506	0.00171314
C2 FTLD-TDP cell-cell adhesion	2.390872751	0.00030811
C2 FTLD-TDP neurogenesis	2.31168566	5.06E-07
C2 FTLD-TDP supramolecular fiber organization	2.283890512	0.00339742
C2 FTLD-TDP cell projection assembly	2.272238009	0.02775009
C2 FTLD-TDP plasma membrane bounded cell projection assembly	2.199778426	0.04967834
C2 FTLD-TDP enzyme-linked receptor protein signaling pathway	2.159314666	0.00298757
C2 FTLD-TDP negative regulation of cellular component organization	2.119292885	0.02858684
C2 FTLD-TDP nervous system development	2.072045544	1.08E-07
C2 FTLD-TDP locomotion	2.055501461	0.00080159
C2 FTLD-TDP positive regulation of cellular component organization	1.97008518	0.00903216
C2 FTLD-TDP cell migration	1.953067833	0.00148314
C2 FTLD-TDP central nervous system development	1.8964448	0.0236587
C2 FTLD-TDP cell motility	1.880693418	0.00149714
C2 FTLD-TDP regulation of cellular component organization	1.872242902	3.49E-05
C2 FTLD-TDP anatomical structure morphogenesis	1.87208282	9.20E-06
C2 FTLD-TDP system development	1.826339083	3.23E-08
C2 FTLD-TDP cellular response to endogenous stimulus	1.819027841	0.00969606
C2 FTLD-TDP cytoskeleton organization	1.810281359	0.00903216
C2 FTLD-TDP regulation of organelle organization	1.791887275	0.04494317
C2 FTLD-TDP cell development	1.740815569	0.00020214
C2 FTLD-TDP multicellular organism development	1.721008093	7.48E-08
C2 FTLD-TDP response to endogenous stimulus	1.708934354	0.01683466
C2 FTLD-TDP positive regulation of cell communication	1.647111979	0.03704921
C2 FTLD-TDP regulation of cell communication	1.639356993	0.00022704
C2 FTLD-TDP regulation of signaling	1.585440256	0.00100056
C2 FTLD-TDP regulation of signal transduction	1.557198076	0.00550724
C2 FTLD-TDP intracellular signal transduction	1.55183044	0.01361393
C2 FTLD-TDP animal organ development	1.537683318	0.00586787
C2 FTLD-TDP anatomical structure development	1.529598483	3.42E-06
C2 FTLD-TDP cellular developmental process	1.522462235	0.00047051

C2 FTLD-TDP	cell differentiation	1.516158442	0.00063411
C2 FTLD-TDP	developmental process	1.513354635	9.41E-07
C2 FTLD-TDP	regulation of response to stimulus	1.414982005	0.01765022
C2 FTLD-TDP	multicellular organismal process	1.376594582	5.84E-05
C2 FTLD-TDP	positive regulation of cellular process	1.356301498	0.00715418
C2 FTLD-TDP	cell communication	1.315035383	0.00715832
C2 FTLD-TDP	signal transduction	1.306405055	0.02112523
C2 FTLD-TDP	cellular response to stimulus	1.305698475	0.00302001
C2 FTLD-TDP	response to stimulus	1.287376341	0.00064914
C2 FTLD-TDP	signaling	1.286073415	0.02366772
C2 FTLD-TDP	positive regulation of biological process	1.271352547	0.04555974
C2 FTLD-TDP	regulation of cellular process	1.208819166	0.00339742
C3 HS-Aging	neuron cell-cell adhesion	17.51382743	0.03628126
C3 HS-Aging	postsynaptic specialization assembly	12.45427729	0.0188749
C3 HS-Aging	hippo signaling	10.37856441	0.00975753
C3 HS-Aging	postsynaptic specialization organization	10.15294344	0.01060613
C3 HS-Aging	neuron recognition	9.531334658	0.01317713
C3 HS-Aging	postsynaptic density organization	9.340707965	0.04543473
C3 HS-Aging	postsynapse assembly	9.112885819	0.04907619
C3 HS-Aging	proteoglycan biosynthetic process	7.076293913	0.03997556
C3 HS-Aging	receptor localization to synapse	6.76862896	0.04543473
C3 HS-Aging	regulation of postsynapse organization	6.41028978	0.00975753
C3 HS-Aging	postsynapse organization	5.37946532	0.00133163
C3 HS-Aging	synapse assembly	4.161701568	0.02162645
C3 HS-Aging	regulation of synapse organization	3.891961652	0.0188749
C3 HS-Aging	regulation of synapse structure or activity	3.797035758	0.02162645
C3 HS-Aging	regulation of cell morphogenesis	3.578815312	0.03215232
C3 HS-Aging	modulation of chemical synaptic transmission	3.05002709	0.00823362
C3 HS-Aging	regulation of trans-synaptic signaling	3.043815223	0.00823362
C3 HS-Aging	neuron projection morphogenesis	3.017767189	0.00133163
C3 HS-Aging	synapse organization	2.955920242	0.01423969
C3 HS-Aging	plasma membrane bounded cell projection morphogenesis	2.945268277	0.00183167
C3 HS-Aging	neuron development	2.939383625	6.92E-06

C3	HS-Aging	cellular component morphogenesis	2.933639436	0.00051754
C3	HS-Aging	cell projection morphogenesis	2.92332142	0.00191615
C3	HS-Aging	cell part morphogenesis	2.838710091	0.00266558
C3	HS-Aging	cell morphogenesis	2.769739581	0.00032843
C3	HS-Aging	generation of neurons	2.762037301	2.19E-06
C3	HS-Aging	neuron differentiation	2.706494887	6.92E-06
C3	HS-Aging	cell morphogenesis involved in neuron differentiation	2.69139043	0.01602431
C3	HS-Aging	cell morphogenesis involved in differentiation	2.680867845	0.00898568
C3	HS-Aging	neuron projection development	2.641816394	0.00067232
C3	HS-Aging	anterograde trans-synaptic signaling	2.608442384	0.00709442
C3	HS-Aging	chemical synaptic transmission	2.608442384	0.00709442
C3	HS-Aging	trans-synaptic signaling	2.584385603	0.0076124
C3	HS-Aging	developmental growth	2.566912112	0.01829224
C3	HS-Aging	cell junction organization	2.524515666	0.01149036
C3	HS-Aging	actin filament-based process	2.515245719	0.0076124
C3	HS-Aging	synaptic signaling	2.511586008	0.00941944
C3	HS-Aging	circulatory system process	2.495013814	0.04205927
C3	HS-Aging	neurogenesis	2.465289492	1.02E-05
C3	HS-Aging	regulation of cell projection organization	2.420610296	0.04111035
C3	HS-Aging	plasma membrane bounded cell projection organization	2.251506154	0.00067232
C3	HS-Aging	cell projection organization	2.197115033	0.00096514
C3	HS-Aging	growth	2.193122468	0.02975521
C3	HS-Aging	nervous system development	2.127055602	1.02E-05
C3	HS-Aging	cytoskeleton organization	2.037299159	0.00769386
C3	HS-Aging	cell adhesion	2.014662502	0.00875573
C3	HS-Aging	anatomical structure morphogenesis	1.928759757	0.00032843
C3	HS-Aging	cell-cell signaling	1.927622516	0.01060705
C3	HS-Aging	cell development	1.893158328	0.00047684
C3	HS-Aging	system development	1.733665386	0.00026258
C3	HS-Aging	animal organ development	1.702111106	0.00362076
C3	HS-Aging	system process	1.6774815	0.03697364
C3	HS-Aging	cell differentiation	1.644033824	0.00067232
C3	HS-Aging	phosphorus metabolic process	1.641659578	0.0188749

C3 HS-Aging	cellular developmental process	1.634193843	0.00067232
C3 HS-Aging	phosphate-containing compound metabolic process	1.622707138	0.02666709
C3 HS-Aging	regulation of cell communication	1.609996406	0.00875573
C3 HS-Aging	intracellular signal transduction	1.609863493	0.03944028
C3 HS-Aging	multicellular organism development	1.604243532	0.00067232
C3 HS-Aging	regulation of signaling	1.587345625	0.01257244
C3 HS-Aging	anatomical structure development	1.536717559	0.00034695
C3 HS-Aging	cellular localization	1.490401821	0.04543473
C3 HS-Aging	developmental process	1.468257378	0.00071945
C3 HS-Aging	localization	1.450109909	0.00643852
C3 HS-Aging	signaling	1.438581236	0.00191615
C3 HS-Aging	cell communication	1.428545585	0.00219601
C3 HS-Aging	signal transduction	1.377731205	0.01905274
C3 HS-Aging	cellular response to stimulus	1.366145295	0.00459353
C3 HS-Aging	multicellular organismal process	1.35583112	0.00497077
C3 HS-Aging	response to stimulus	1.316606783	0.00333607
C5 FTLD-HS	negative regulation of protein phosphorylation	13.11180124	0.02994303
C5 FTLD-HS	negative regulation of phosphorylation	12.02849003	0.02994303
C5 FTLD-HS	negative regulation of phosphate metabolic process	10.37346437	0.03048542
C5 FTLD-HS	negative regulation of phosphorus metabolic process	10.34803922	0.03048542
