# Supplementary material for: Polygenic associations with clinical and neuropathological trait heterogeneity across TDP-43 proteinopathies
Source: PLoS One. 2025 Dec 30;20(12):e0338398. doi: 10.1371/journal.pone.0338398 (PMC12752993; doi:10.1371/journal.pone.0338398)
Supplement: S3 File — (PDF) [file pone.0338398.s010.pdf]

Chromosome	Position_GRCh38	rsID
chr3	58868540	rs17360960
chr10	107081261	rs17209374
chr2	33818359	rs13417671
chr2	20543956	rs11096672
chr10	83990267	rs10887195
chr4	6876175	rs6828754
chr3	98747191	rs828598
chr16	4885635	rs2075639
chr6	66950965	rs6930795
chr8	39692348	rs7465283
chr5	151052885	rs17111695
chr4	37769347	rs1030350
chr1	216540972	rs17042779
chr13	53017291	rs4884317
chr9	19126567	rs3824369
chr14	100148698	rs878078
chr17	31729919	rs9909665
chr8	17598351	rs2588139
chr13	48074014	rs9567990
chr3	171223294	rs7374644
chr21	39010116	rs2236436
chr4	20482633	rs17612037
chr8	51447983	rs7846408
chr4	2309634	rs4974674
chr15	27629529	rs3098551
chr12	6386109	rs2364480
chr7	8992665	rs10255740
chr13	88252095	rs4278614
chr9	27502988	rs1977661
chr13	98447084	rs2281767
chr8	75235719	rs16939046
chr18	77005659	rs509620
chr12	28084361	rs231210
chr2	227706159	rs7585761
chr13	21152899	rs729105
chr17	41968753	rs8078650
chr17	57034731	rs8078110
chr11	43998165	rs10838212
chr4	80078917	rs7693145
chr15	98898207	rs1464433
chr19	6833766	rs186295
chr3	376902	rs3773379

chr17	9220577	rs9897341
chr1	164847489	rs6426881
chr3	65653197	rs1499497
chr5	113718283	rs985411
chr3	13841863	rs9863149
chr5	8439243	rs4461605
chr14	79854741	rs17764956
chr4	109733484	rs2346841
chr2	138690053	rs7594562
chr9	108098233	rs10979206
chr5	173184645	rs255313
chr3	10458120	rs12492242
chr8	11908511	rs6993684
chr8	6771406	rs17078076
chr18	34076385	rs4239384
chr10	131126683	rs1106524
chr1	4321193	rs2171985
chr6	81725071	rs12206095
chr22	43122269	rs742134
chr4	95414339	rs17439081
chr1	20078672	rs521179
chr15	60971178	rs17204698
chr3	72122732	rs17009412
chr7	43660747	rs10281917
chr6	12302337	rs11755836
chr11	78453938	rs4945276
chr10	5654519	rs3750640
chr1	237291723	rs7538075
chr4	703059	rs12646225
chr5	113344640	rs1116547
chr1	34149975	rs4653395
chr17	53192146	rs17626856
chr2	238590694	rs1510510
chr12	72346482	rs4309196
chr5	61677470	rs391101
chr8	128988278	rs7829061
chr3	55275572	rs7649746
chr17	28396803	rs739439
chr4	174304301	rs4695918
chr5	82666969	rs2055438
chr1	52590925	rs11591099
chr16	19449520	rs6497373
chr10	27434104	rs7342136

chr4	152714217	rs11930234
chr13	73448776	rs7327345
chr1	76373234	rs11162133
chr5	128837115	rs2158175
chr5	82882038	rs4383715
chr5	127320808	rs152120
chr12	71559910	rs7973299
chr7	42348598	rs1012325
chr2	204192945	rs13401647
chr8	11789425	rs904015
chr3	51058304	rs4927960
chr4	140891491	rs1863307
chr5	133190859	rs7715671
chr12	63051312	rs10506458
chr9	6672097	rs1658957
chr1	224076680	rs10916260
chr12	57454856	rs3809114
chr1	23212517	rs10917360
chr3	44570558	rs9873604
chr11	88377297	rs10765490
chr4	1318606	rs17742599
chr14	44172432	rs1040691
chr14	76519067	rs1642851
chr8	9030178	rs2953807
chr2	59884087	rs12991146
chr11	122770548	rs11218802
chr5	178902845	rs953741
chr17	75898723	rs7370
chr8	40947632	rs7827529
chr10	69643146	rs10509319
chr1	114685275	rs2268699
chr11	102733876	rs2155053
chr7	133620975	rs6956399
chr8	132282725	rs1457780
chr9	21925856	rs7852128
chr16	12583902	rs7197841
chr2	104983381	rs4851727
chr1	22770325	rs7516175
chr2	230925382	rs3111780
chr9	10295912	rs616040
chr14	47826113	rs1956314
chr1	161242663	rs11584174
chr16	5359850	rs7198653

chr3	111604007	rs7431849
chr2	228291114	rs10498227
chr12	116838412	rs10047539
chr1	228939221	rs6701969
chr4	85818652	rs12498568
chr16	81282928	rs11865869
chr4	169393217	rs7672874
chr15	83227856	rs7162082
chr2	239892262	rs10933613
chr6	83116819	rs7764458
chr2	168793861	rs3845727
chr15	70348536	rs6494855
chr6	135800421	rs7767870
chr10	14670444	rs1459019
chr9	27586164	rs2453556
chr1	180169665	rs6703180
chr12	22851463	rs2467458
chr13	102700673	rs12427471
chr13	87319081	rs7999126
chr14	31503083	rs2378931
chr6	169217631	rs8089
chr8	11554314	rs17744726
chr12	50787916	rs10783388
chr9	68982398	rs4745451
chr12	64213702	rs812590
chr9	90110024	rs7851675
chr8	72824531	rs1107217
chr11	123149523	rs12578013
chr11	34485666	rs1980719
chr21	26996145	rs2830607
chr7	103552269	rs362791
chr16	81148012	rs4889247
chr11	57610852	rs2511989
chr21	41888378	rs4920102
chr12	65330944	rs10878260
chr6	103176933	rs494028
chr17	31679811	rs7210009
chr3	66662140	rs9839845
chr2	111032214	rs13012948
chr10	132834775	rs12356978
chr4	137358691	rs10519423
chr1	204938656	rs2802822
chr6	108760394	rs566575

chr6	50661441	rs280317
chr6	120635397	rs9401311
chr12	109438323	rs10850141
chr14	20656875	rs8003288
chr15	33722317	rs11635093
chr6	170483679	rs12189977
chr4	158698883	rs7679753
chr9	27478054	rs1822723
chr15	82896041	rs7496832
chr22	44310934	rs12158741
chr2	31004165	rs12991678
chr2	78968955	rs17693274
chr4	108742458	rs17527308
chr9	27519175	rs11792285
chr20	59392851	rs1877751
chr15	85838928	rs7180790
chr14	30630532	rs229150
chr4	1526039	rs7666926
chr13	28318848	rs9319425
chr2	163169687	rs1489636
chr4	107678390	rs727137
chr6	151261497	rs9383562
chr7	37901650	rs1530820
chr3	60103845	rs10510827
chr14	72002874	rs2239268
chr13	71699240	rs7993403
chr15	32959526	rs6494794
chr12	57893419	rs17119981
chr10	131950560	rs4880246
chr3	130020067	rs6439205
chr8	58436494	rs6993992
chr11	88169611	rs302648
chr14	67499905	rs10873202
chr20	21043741	rs6047216
chr1	239898335	rs10802816
chr6	23035104	rs2498259
chr1	180572227	rs12030731
chr1	150986360	rs267733
chr5	167596996	rs1903110
chr2	51785140	rs1516199
chr4	69441766	rs12650761
chr19	51529101	rs8182477
chr1	237032806	rs12032218

chr7	106508773	rs17153258
chr5	119079098	rs11742151
chr17	78368412	rs8074003
chr6	2954323	rs2295767
chr5	167475105	rs7378751
chr18	56187234	rs4570960
chr15	82161045	rs8034586
chr5	180307312	rs3763131
chr2	5690467	rs3922853
chr16	77677207	rs7193406
chr5	171141736	rs4868069
chr3	23157962	rs9818043
chr3	146126586	rs4681298
chr14	92133833	rs17807992
chr10	129796586	rs1887181
chr13	23417765	rs7320968
chr3	97883893	rs10935130
chr17	77012989	rs6501930
chr19	52118391	rs8111640
chr6	166947236	rs1079145
chr2	44675221	rs12612153
chr9	28298085	rs10968468
chr14	32259027	rs6571501
chr6	72405882	rs11754392
chr8	11722293	rs10112596
chr5	113591058	rs2303718
chr3	187500711	rs2600990
chr11	9565931	rs7937639
chr4	41705076	rs12650313
chr14	96865281	rs10135704
chr14	92037924	rs2235978
chr12	39803007	rs4254134
chr4	99831995	rs2162386
chr10	110673089	rs10509925
chr7	22276689	rs1636899
chr18	50276088	rs140687
chr5	174322572	rs6861937
chr14	30116428	rs190123
chr6	151254115	rs7764018
chr8	2560586	rs6996532
chr4	154051916	rs6535980
chr9	112634926	rs11789059
chr13	39268126	rs1413056

chr8	106830067	rs2930492
chr2	58758906	rs10176091
chr12	46657293	rs10492250
chr8	22585707	rs11994391
chr4	7432290	rs10033032
chr21	16375756	rs370428
chr18	46588774	rs1078174
chr20	63899393	rs4809244
chr1	180200270	rs7513063
chr4	114908763	rs7675371
chr5	153418119	rs13361457
chr17	11059042	rs11078879
chr4	167041297	rs10517919
chr5	151181776	rs1061837
chr4	101786634	rs17199964
chr15	96658186	rs4335743
chr9	98171545	rs4743179
chr5	129427451	rs12656498
chr12	71640351	rs1149009
chr1	13646755	rs12561812
chr2	171847131	rs925881
chr13	25676590	rs17082637
chr5	134594838	rs17167632
chr1	17086006	rs2076616
chr7	122677224	rs4140960
chr5	5938358	rs1174902
chr13	107575531	rs9301233
chr4	8727745	rs10755200
chr10	12755759	rs4132050
chr10	15354752	rs7083529
chr12	57584957	rs775251
chr10	100641711	rs2495712
chr21	39914089	rs2837291
chr9	36782016	rs2381583
chr5	16837753	rs17651266
chr22	45188031	rs9614616
chr12	64470203	rs11837890
chr9	130014745	rs10760653
chr10	78566097	rs164226
chr5	108696765	rs2174973
chr2	179643054	rs10930879
chr9	12234969	rs946452
chr4	146076224	rs1035803

chr6	27427985	rs6926142
chr20	63922145	rs6090020
chr2	75999249	rs6708139
chr9	27572257	rs2282241
chr7	25260370	rs2107124
chr13	92717616	rs1933192
chr11	8240854	rs3849994
chr21	31064491	rs2833249
chr10	93448022	rs809812
chr7	101049095	rs6968731
chr10	6262594	rs4750195
chr9	128016235	rs7026544
chr10	59620687	rs2126772
chr2	48352639	rs7606104
chr16	77734943	rs308927
chr22	31096496	rs2074736
chr3	100874422	rs9863850
chr6	152658699	rs9383636
chr1	181033343	rs3806284
chr18	22499276	rs494470
chr21	34991865	rs11088302
chr16	13571085	rs2226994
chr12	2600714	rs215986
chr4	98546473	rs6532746
chr21	16376665	rs435082
chr4	158756560	rs7666007
chr22	45259963	rs1533371
chr20	49975221	rs6020157
chr10	52214509	rs12256137
chr7	43307079	rs12532870
chr17	78396063	rs17561950
chr8	143944186	rs11786896
chr8	135363415	rs7813136
chr8	19652775	rs17128793
chr3	175451941	rs6801770
chr6	90902361	rs9451525
chr10	106778012	rs12256169
chr1	101048152	rs11166538
chr2	106583062	rs968914
chr18	25459550	rs4800633
chr2	216663886	rs9341218
chr2	35118154	rs2119082
chr6	34514490	rs11753634

chr12	64336726	rs10784404
chr7	5686391	rs4724712
chr8	58281277	rs4602872
chr18	8355050	rs7226445
chr11	78114274	rs665278
chr9	27468463	rs10511816
chr2	137641336	rs881639
chr3	899015	rs2313182
chr21	32830972	rs6517112
chr2	104873939	rs2060783
chr14	34331617	rs1958578
chr10	31700680	rs7901343
chr4	14007236	rs9291619
chr6	16805989	rs11752823
chr5	179523760	rs1344158
chr1	33436150	rs7544329
chr7	13838346	rs10230244
chr3	71316220	rs17655524
chr8	8384155	rs4840341
chr17	5478547	rs3865350
chr8	129447166	rs1368136
chr3	57985796	rs1866164
chr17	55381043	rs8066468
chr17	28269102	rs9909055
chr17	5492063	rs8081387
chr1	233127221	rs6691107
chr12	64453031	rs7316037
chr14	93232657	rs740791
chr5	3503064	rs4866578
chr5	29249437	rs7717683
chr7	19476869	rs2192491
chr11	103859255	rs12574570
chr1	102765472	rs618072
chr6	70956443	rs1320315
chr22	34292320	rs1489877
chr1	18799290	rs12042960
chr3	3013563	rs17024684
chr1	4337468	rs684965
chr4	41207844	rs4590083
chr1	224095362	rs1573185
chr3	66671258	rs6782747
chr3	60424454	rs241692
chr4	2342124	rs2071678

chr7	155437846	rs6948976
chr2	1618368	rs9752413
chr20	61693013	rs12479878
chr17	78399553	rs2292641
chr13	30561355	rs9508801
chr16	84424708	rs247804
chr9	34017108	rs1785506
chr18	3087945	rs948299
chr7	4406242	rs1447402
chr3	14200648	rs2305843
chr2	3337168	rs11885038
chr5	77295873	rs12514694
chr19	16624977	rs3786603
chr11	123112220	rs4936777
chr17	76916871	rs12603183
chr4	47509764	rs10000432
chr12	39814235	rs10784079
chr1	204991111	rs3820336
chr5	151094113	rs999556
chr5	38135616	rs3812048
chr15	40033659	rs8042947
chr5	136413179	rs1499772
chr17	36025629	rs854658
chr13	114275850	rs9562187
chr6	161902965	rs6455783
chr6	2970334	rs7743193
chr11	36944654	rs7109945
chr14	31829453	rs12886280
chr6	73970322	rs3003187
chr4	134500552	rs10006423
chr11	99527840	rs2515376
chr10	129562960	rs511770
chr10	92760912	rs10509646
chr20	41393111	rs4810316
chr13	70416742	rs12583232
chr17	42398593	rs4796649
chr4	123556545	rs4076851
chr12	57618450	rs11613457
chr5	38735182	rs17456364
chr3	157068755	rs11719102
chr7	22272446	rs17146488
chr10	132124775	rs7083603
chr6	46850245	rs6458525

chr13	66860029	rs9564355
chr9	68856996	rs6560397
chr5	96291166	rs13158163
chr2	163975310	rs165231
chr15	60392977	rs11631777
chr19	20025895	rs8106847
chr1	22170210	rs909813
chr13	102292510	rs17505423
chr4	168675498	rs13137200
chr3	146143518	rs1707469
chr11	58287727	rs12362065
chr6	11500384	rs9394119
chr5	34826684	rs10461948
chr9	98366576	rs3780429
chr4	29724472	rs12641755
chr2	177283476	rs2364731
chr2	230920311	rs16827668
chr10	16903194	rs7099855
chr1	114601683	rs12730318
chr16	11269835	rs415595
chr10	73799109	rs2271271
chr7	42388980	rs12530988
chr6	66268753	rs7763510
chr10	77406587	rs670898
chr5	41617154	rs17276531
chr15	82942343	rs7170046
chr10	27521938	rs2642274
chr1	211181496	rs10494934
chr16	79530084	rs9936252
chr15	93463669	rs16948067
chr7	152678103	rs2040639
chr4	121321648	rs12650310
chr2	200779509	rs2348129
chr10	89913	rs7906287
chr16	7392248	rs4786149
chr21	44835280	rs9808690
chr8	699190	rs12156406
chr12	50695504	rs2139930
chr1	4560863	rs10915560
chr1	209418897	rs11580645
chr18	52429089	rs1145242
chr17	31756668	rs7212433
chr14	53584358	rs7152946

chr6	40885000	rs4711652
chr21	26054932	rs2830028
chr9	129904796	rs6478924
chr9	102187693	rs10820085
chr9	18973920	rs6475294
chr8	26282307	rs4545109
chr4	25016113	rs10488953
chr22	40099469	rs6001762
chr9	27543283	rs3849942
chr4	79477554	rs10027646
chr21	21613860	rs2739356
chr9	34124862	rs2275003
chr21	29806309	rs2256853
chr6	156265619	rs9478696
chr20	671453	rs753490
chr8	143358686	rs2931710
chr18	74147203	rs3813119
chr18	74190040	rs7240367
chr7	5455046	rs6964614
chr7	137986794	rs11770714
chr9	27596680	rs10812621
chr15	82809417	rs8042254
chr1	181070392	rs17302632
chr10	100219661	rs11595324
chr12	56280982	rs773652
chr2	40581909	rs10198059
chr16	667523	rs3752493
chr1	152286602	rs11204971
chr8	9103507	rs1809006
chr6	155428270	rs231951
chr5	10219056	rs10866471
chr7	37438260	rs17171046
chr15	82176114	rs1501372
chr6	122657277	rs2316962
chr12	57725064	rs4760169
chr2	112820380	rs7596461
chr11	82524933	rs2226620
chr4	169619890	rs3797041
chr7	13963480	rs2282869
chr7	71300110	rs1317429
chr13	105251989	rs3015363
chr16	674084	rs1045763
chr15	25708051	rs17555920

chr10	127507271	rs10830109
chr2	151475231	rs16830067
chr1	102451358	rs2376037
chr17	8473883	rs11650137
chr8	23392434	rs7824768
chr4	25940970	rs1456435
chr14	97004831	rs8017230
chr2	222231249	rs1549773
chr13	40181504	rs10507482
chr1	63121375	rs17124643
chr5	113986227	rs12658199
chr7	30745210	rs4645485
chr5	160656487	rs2217637
chr17	57376248	rs1982270
chr5	178964901	rs12719860
chr18	75528719	rs11665332
chr14	49943829	rs941605
chr10	49593296	rs1917804
chr10	132141327	rs10870265
chr6	9116165	rs6911727
chr8	3785733	rs1383951
chr6	167283460	rs3010558
chr14	99619113	rs2144800
chr6	157035957	rs2207227
chr1	57175802	rs7530049
chr1	48610454	rs320019
chr9	27559735	rs1565948
chr5	169973390	rs13165483
chr2	75959356	rs2197078
chr4	77776632	rs9307306
chr7	103537500	rs362710
chr9	34983977	rs4879847
chr1	224064136	rs7531519
chr6	121452853	rs2389541
chr1	110861171	rs12026259
chr18	37390664	rs488797
chr5	179517406	rs936817
chr2	42388356	rs7560797
chr2	103919258	rs1441104
chr10	28432348	rs11007106
chr13	85955140	rs7997693
chr13	45524859	rs3014966
chr11	21041143	rs11025886

chr7	88719132	rs1966296
chr11	79473855	rs7102569
chr20	56861662	rs6069911
chr10	111994786	rs1421050
chr6	148448972	rs7763869
chr6	108714193	rs522611
chr9	2140197	rs7035986
chr6	10887018	rs9366663
chr3	71311557	rs7633896
chr2	164010159	rs357309
chr11	35167698	rs353626
chr2	88460447	rs11693918
chr3	196990442	rs569330
chr2	222014814	rs17315907
chr11	82354196	rs7930387
chr6	155539784	rs11156029
chr6	52262783	rs1413920
chr2	59251853	rs5008666
chr5	113451146	rs26990
chr2	129063127	rs10928866
chr18	75573361	rs952058
chr1	86116996	rs1507292
chr20	34737712	rs1018503
chr10	128448909	rs9804335
chr15	60960980	rs12903220
chr14	30669172	rs12894695
chr14	48217286	rs4319673
chr6	127725568	rs564461
chr11	5904202	rs11039518
chr7	148303600	rs1110038
chr1	7232712	rs4908608
chr11	73806214	rs11235911
chr13	48557633	rs4942767
chr13	110292448	rs2391823
chr16	85193213	rs11646864
chr2	145586665	rs10186882
chr13	33227831	rs17078802
chr12	64316939	rs12308116
chr3	873706	rs1464701
chr21	16257417	rs2823702
chr17	14303045	rs719470
chr9	22911501	rs10965517
chr6	165384456	rs11754977

chr16	8565207	rs4580141
chr5	116607498	rs607930
chr8	10477522	rs7842777
chr18	38551867	rs1365468
chr5	157914383	rs17608701
chr5	144672484	rs17387218
chr5	142283199	rs4912841
chr4	79677642	rs13123790
chr12	64521280	rs1317532
chr20	49938133	rs2038127
chr5	178330763	rs2913788
chr16	50592284	rs7204135
chr6	86234881	rs7765374
chr12	51112545	rs4768967
chr4	186410505	rs11945366
chr6	156068014	rs4870428
chr5	108512271	rs9326729
chr17	43891933	rs1642592
chr3	72217160	rs4677113
chr8	22318020	rs4242431
chr15	51602114	rs12372948
chr12	8055648	rs11057065
chr15	91312351	rs8026639
chr12	77921555	rs2731424
chr21	19696842	rs1850587
chr12	124660848	rs838875
chr5	133447495	rs25869
chr2	170572859	rs11693682
chr5	53537360	rs10078892
chr9	10292422	rs10959029
chr7	78729198	rs4370470
chr10	128449863	rs7100024
chr4	112494246	rs7699752
chr6	25151193	rs9356963
chr2	7826846	rs7601867
chr5	68958125	rs11951431
chr10	16792661	rs3824633
chr8	10403043	rs1962073
chr10	37411269	rs11011172
chr9	27233970	rs1590255
chr4	62329832	rs1478528
chr13	51064099	rs6561611
chr9	106556480	rs1387596

chr6	169040761	rs7765333
chr1	153668351	rs7536700
chr14	33269492	rs1500719
chr7	36689719	rs4077337
chr16	80883984	rs9935116
chr6	148516555	rs17715583
chr6	162651750	rs1893540
chr7	8988208	rs6979515
chr5	31951855	rs16901697
chr5	151066407	rs3792789
chr14	103528624	rs1467561
chr3	104041332	rs4420906
chr15	60977248	rs8031801
chr12	122043384	rs12827036
chr6	106497026	rs9480677
chr1	234144826	rs12073596
chr7	155435665	rs11768588
chr16	50711288	rs2066843
chr6	23089271	rs12523660
chr16	76982383	rs10431977
chr5	19048511	rs4333315
chr6	9137048	rs10484328
chr9	34023574	rs1785502
chr4	87622676	rs2627730
chr9	112197858	rs10981305
chr18	37334783	rs3865390
chr6	93321933	rs164302
chr15	92590195	rs285718
chr13	73182499	rs9600085
chr11	133726227	rs10894740
chr17	67379776	rs11652752
chr7	9097190	rs2286111
chr1	161751917	rs1063178
chr22	50473447	rs4824152
chr1	201750207	rs239989
chr4	134242606	rs2421243
chr22	44924751	rs9614985
chr1	96649493	rs6673531
chr22	44149485	rs5764560
chr1	92080868	rs12739985
chr18	6600606	rs11659717
chr2	228318301	rs16824642
chr1	223663031	rs1109223

chr11	22318896	rs7935452
chr9	106848175	rs4333673
chr5	172920900	rs7717884
chr9	74833606	rs11144108
chr5	38036178	rs10473079
chr16	9777611	rs2267779
chr13	44234038	rs9533799
chr17	37863709	rs10512474
chr9	15288047	rs10810369
chr3	134655756	rs11719419
chr18	56238709	rs1229585
chr5	76509280	rs6453227
chr12	99997310	rs7306416
chr18	69534681	rs1442669
chr19	7693453	rs12971845
chr14	47860435	rs451505
chr3	1074220	rs1857699
chr6	1529552	rs2569865
chr20	34598676	rs2378199
chr2	181264071	rs1899037
chr1	213282877	rs1187802
chr16	60061475	rs36553
chr9	23569872	rs17698074
chr15	97480552	rs8038622
chr1	161234748	rs2501873
chr3	14263841	rs13072044
chr12	58176018	rs17120420
chr21	40886403	rs13049240
chr1	29815360	rs2166884
chr12	111446804	rs3184504
chr3	114298411	rs2693051
chr8	142040310	rs4917300
chr18	27067723	rs10502485
chr7	107836101	rs4730271
chr9	68603133	rs1888722
chr5	143176167	rs6894946
chr16	77809313	rs2914446
chr9	27609516	rs810174
chr17	27524665	rs2945378
chr11	59861832	rs557564
chr11	14581152	rs17567703
chr1	55539504	rs1740113
chr5	113939678	rs4705607

chr14	76838946	rs6574333
chr7	125265006	rs11771079
chr6	53098273	rs2876
chr11	126498537	rs11823106
chr1	103087379	rs1415363
chr1	160407087	rs6663715
chr6	13597890	rs7759452
chr11	95341096	rs514284
chr18	32683529	rs1403755
chr2	224151769	rs12619528
chr2	33822143	rs7565016
chr17	33285735	rs1497366
chr6	31113122	rs2233965
chr1	93769311	rs2747038
chr5	173827349	rs1506726
chr9	130149826	rs7866942
chr1	6508327	rs4908554
chr3	137488261	rs4234226
chr1	86022231	rs11161721
chr14	59198052	rs1252917
chr7	8984751	rs6962574
chr10	29787425	rs10826694
chr5	118459959	rs10046044
chr10	131150577	rs7081792
chr11	127211696	rs4529888
chr17	54721227	rs758647
chr7	142727107	rs6959895
chr15	33007031	rs12441885
chr14	55966754	rs1188147
chr2	65168812	rs11903118
chr10	13832453	rs10906506
chr15	63586733	rs7183892
chr3	29669222	rs7634507
chr7	38144185	rs1357647
chr21	23110971	rs2408666
chr22	23366646	rs5996528
chr1	197999256	rs1125953
chr3	70856430	rs2687195
chr3	29749272	rs6792183
chr2	42235790	rs13416119
chr8	10367649	rs7015455
chr13	37929941	rs4943550
chr6	73975267	rs9293946

chr7	21339069	rs6461554
chr15	60980538	rs1589703
chr13	89522272	rs2221406
chr5	76891483	rs7722773
chr4	4937696	rs11946368
chr17	57632675	rs3826301
chr5	22849375	rs4701204
chr3	58669165	rs1444186
chr2	76005200	rs4853189
chr17	58097128	rs8071026
chr2	29731798	rs2631956
chr17	53033072	rs792768
chr3	186115970	rs7635103
chr11	75143624	rs10899075
chr2	201999764	rs4675246
chr12	108467757	rs2111949
chr1	167446554	rs1554669
chr2	127762973	rs6731093
chr14	96681891	rs10484208
chr2	224346689	rs6745530
chr9	134005665	rs419218
chr17	42166716	rs9912576
chr9	88188557	rs17053864
chr2	207807187	rs10932209
chr18	25434737	rs12971136
chr1	22814207	rs2869511
chr3	153790428	rs924825
chr3	171962723	rs7619106
chr5	151052827	rs871269
chr1	207062609	rs2075863
chr8	8351675	rs4840932
chr10	7655541	rs11255267
chr8	5514091	rs1011000
chr1	158766655	rs1864346
chr1	96973935	rs3002273
chr1	204986288	rs11240326
chr6	140315494	rs1157002
chr5	151022235	rs3828599
chr14	88963991	rs1885185
chr1	180815742	rs2944259
chr9	136027362	rs7388909
chr11	93510356	rs2605618
chr2	80343381	rs1373264

chr19	49374939	rs2288481
chr4	54675242	rs6820303
chr4	129942948	rs10012483
chr4	38425077	rs7679805
chr5	109108853	rs4957798
chr5	22826012	rs1661975
chr10	6480544	rs3793729
chr8	138773995	rs11781300
chr2	119520595	rs4383344
chr7	46573011	rs10261793
chr6	118278665	rs1889468
chr7	36691170	rs4072404
chr8	11778508	rs4840584
chr3	44645808	rs1402752
chr12	40580596	rs12422396
chr20	36338061	rs6024358
chr10	111410490	rs9421093
chr21	19860120	rs2407489
chr4	120666114	rs1580743
chr2	218741495	rs1554622
chr2	179585255	rs16866809
chr8	58547499	rs12375332
chr12	64802197	rs7132617
chr1	106249336	rs7543568
chr20	44391463	rs3092370
chr6	5387280	rs2432755
chr9	11022581	rs10959466
chr21	38361607	rs2836340
chr7	24784176	rs17211952
chr13	113570181	rs4907646
chr14	82217289	rs1457979
chr13	88380811	rs978165
chr7	22361019	rs7785685
chr1	163397064	rs10799916
chr8	23401864	rs17696480
chr7	55056275	rs11773818
chr2	47004933	rs11899526
chr12	89386220	rs7305095
chr11	45106751	rs3740787
chr21	45207772	rs2838813
chr18	58370256	rs2075404
chr17	13452712	rs8080163
chr6	34515238	rs4713808

chr10	112342122	rs11195905
chr6	52326459	rs6458829
chr10	48790524	rs726786
chr1	85996226	rs11161714
chr2	167646759	rs1147155
chr2	183606069	rs6710374
chr11	117690550	rs10892162
chr3	39481512	rs1768208
chr10	75058179	rs1993005
chr11	113956604	rs17116178
chr13	66913281	rs1323922
chr4	123827822	rs979755
chr13	99240059	rs2296911
chr1	7698700	rs12756299
chr18	44018694	rs4500824
chr12	25681937	rs10771232
chr6	162847872	rs1333962
chr7	153048725	rs4725479
chr12	64338514	rs11609829
chr18	78874943	rs936501
chr4	47531821	rs7674353
chr14	55077758	rs749053
chr14	30766309	rs179534
chr12	108332900	rs10735429
chr10	131944854	rs9419387
chr5	173229188	rs3095872
chr1	87774383	rs2436974
chr9	13106599	rs3264
chr4	83239126	rs7658518
chr14	34091538	rs4982138
chr8	105280710	rs16872638
chr16	77750112	rs9927857
chr5	88861735	rs4518438
chr11	23147258	rs11027007
chr20	402232	rs6037542
chr20	36372640	rs2425222
chr6	156360035	rs9384456
chr12	111536476	rs616668
chr18	44100804	rs1607100
chr10	2713735	rs945142
chr1	164680043	rs6426879
chr14	32858311	rs990721
chr15	81011791	rs11072972

chr7	26519873	rs10240247
chr12	126998884	rs11058903
chr9	69005698	rs10869686
chr2	241130647	rs3213680
chr4	101866266	rs4698977
chr5	96773540	rs28096
chr7	9010163	rs13241564
chr17	83073892	rs8072895
chr2	51793222	rs17864614
chr4	41130074	rs2256007
chr3	75196623	rs9828201
chr19	45351661	rs13181
chr15	79029175	rs10851915
chr11	41109279	rs578718
chr19	45408744	rs735482
chr4	133166608	rs17020496
chr8	92345976	rs1125730
chr1	237643519	rs11583646
chr1	234314248	rs561159
chr6	46407589	rs2153615
chr10	118637083	rs877721
chr18	23732088	rs2337186
chr6	9301633	rs11967465
chr1	224213894	rs6689187
chr8	54892163	rs13249752
chr4	99321852	rs1159918
chr11	107558697	rs1950031
chr12	89378126	rs704061
chr2	7986660	rs2111074
chr4	169991127	rs7661337
chr5	81949312	rs11740142
chr3	14393563	rs721377
chr11	107494465	rs4754226
chr14	52417362	rs809100
chr16	6412230	rs9926622
chr8	133049467	rs2252917
chr2	217077992	rs2372960
chr16	4880099	rs1876359
chr16	84751597	rs8058755
chr6	11612742	rs515145
chr16	10066931	rs1448259
chr7	95481979	rs43065
chr11	73837225	rs1792165

chr15	83049857	rs12907032
chr12	29658538	rs16934692
chr2	135901925	rs12478902
chr12	130229408	rs549257
chr7	153127146	rs940650
chr13	102673692	rs660596
chr2	179568810	rs999000
chr5	7983578	rs11748296
chr8	136274480	rs13260793
chr4	60268481	rs6853571
chr7	53716938	rs1012052
chr7	26852046	rs7804356
chr14	53554017	rs10145210
chr12	68402668	rs7307444
chr9	34085861	rs7036030
chr20	58631557	rs6100145
chr12	64091580	rs789744
chr6	27332801	rs1156457
chr8	1733567	rs2972180
chr6	42252734	rs12194245
chr1	93748070	rs7552104
chr10	105962048	rs517866
chr9	17616195	rs3808755
chr19	14566500	rs3752221
chr4	31865585	rs6827011
chr9	8180576	rs10815776
chr5	151165010	rs7731328
chr17	11184970	rs9891347
chr1	162044842	rs2880058
chr2	241454726	rs764081
chr4	136930704	rs1373895
chr16	85337082	rs4783169
chr3	2293378	rs11915941
chr1	5238999	rs551536
chr7	125053989	rs1404897
chr4	117636163	rs17862059
chr1	50644495	rs12567589
chr8	73318674	rs16938613
chr6	120608745	rs1203087
chr4	72278465	rs1369095
chr6	153122024	rs6557273
chr17	48869635	rs12449856
chr9	114133316	rs10982083

chr2	24267544	rs4268898
chr4	8116834	rs10938692
chr12	64092505	rs7960270
chr11	133816572	rs2851128
chr8	54559955	rs7827437
chr18	12516769	rs11663391
chr18	30276169	rs2960051
chr14	30770756	rs179525
chr5	88812757	rs700588
chr9	129944885	rs7870884
chr8	53396524	rs10958356
chr1	186179900	rs1407434
chr10	33410745	rs2785273
chr17	36507271	rs8081787
chr1	1869595	rs4648592
chr20	50195590	rs2904254
chr8	3011224	rs11786445
chr3	191227844	rs6801419
chr3	7798131	rs721257
chr4	2829656	rs231399
chr2	112948594	rs3923566
chr13	83498434	rs9575317
chr5	113718683	rs6885608
chr2	114864622	rs13007523
chr3	47998793	rs11711953
chr4	28024330	rs4140905
chr4	109627238	rs10017480
chr16	84979651	rs9935170
chr8	58116301	rs9792139
chr10	106634856	rs10491052
chr17	78500101	rs2028733
chr8	135634305	rs4909494
chr22	43986973	rs2294928
