# Supplementary material for: Polygenic associations with clinical and neuropathological trait heterogeneity across TDP-43 proteinopathies
Source: PLoS One. 2025 Dec 30;20(12):e0338398. doi: 10.1371/journal.pone.0338398 (PMC12752993; doi:10.1371/journal.pone.0338398)
Supplement: S4 File — (PDF) [file pone.0338398.s011.pdf]

Chromosome	Position_GRCh38	rsID
chr22	42477526	rs13057831
chr15	26265263	rs7183534
chr4	94658554	rs11097432
chr1	100810988	rs6699536
chr8	118103756	rs11562695
chr19	52396303	rs324119
chr14	91690317	rs11160024
chr7	26190552	rs1049938
chr18	6384432	rs1436907
chr3	148495609	rs3915005
chr4	61244052	rs2342453
chr10	84871785	rs11201267
chr12	4540899	rs10849087
chr4	120788775	rs13111620
chr6	143610595	rs6570567
chr8	77161703	rs12675877
chr9	14880669	rs7862716
chr8	142593861	rs7843869
chr7	112167721	rs2523017
chr1	237179412	rs12746235
chr3	23146552	rs9879958
chr10	75496823	rs3012041
chr1	206771966	rs3024490
chr15	59479763	rs7161900
chr6	44472985	rs9395021
chr6	18515745	rs6459621
chr2	217229157	rs6435966
chr3	69062361	rs9851078
chr1	97246005	rs12758854
chr21	23228066	rs2827974
chr5	74423835	rs460738
chr7	126513322	rs2237734
chr1	31971667	rs624017
chr13	98111979	rs9513355
chr22	29142480	rs713728
chr7	31707635	rs12669290
chr15	71262825	rs4777352
chr19	51683264	rs2902883
chr3	97056677	rs928958
chr5	110035287	rs2900041
chr4	21502990	rs187196
chr8	76722558	rs966535

chr9	126724703	rs3850586
chr16	52027135	rs12149256
chr7	108434322	rs2284290
chr14	79834334	rs2293829
chr18	72635086	rs4892018
chr6	126160262	rs1330674
chr3	67410572	rs6782194
chr3	48503583	rs7434077
chr2	151083575	rs12692951
chr5	169092640	rs11744892
chr5	177992966	rs1800197
chr1	60942867	rs543314
chr15	53606991	rs10152972
chr1	205267105	rs4951182
chr5	133521361	rs155578
chr9	132423288	rs7470548
chr5	100878913	rs13355889
chr9	110706320	rs4623510
chr7	29407425	rs7800059
chr5	9373336	rs42192
chr6	27259186	rs9393796
chr17	38283293	rs4329955
chr14	67251254	rs7152903
chr10	19904868	rs11592239
chr3	20528881	rs1403651
chr13	43128003	rs2657107
chr6	4515317	rs11242955
chr1	205292046	rs1172122
chr14	30338688	rs7160082
chr9	6910916	rs12235748
chr5	86451054	rs1500258
chr11	128375436	rs11221267
chr3	23344386	rs13076527
chr16	86089016	rs13336935
chr14	95794905	rs1951981
chr5	4588380	rs1541834
chr4	184441883	rs793794
chr22	26337333	rs6005016
chr2	19539249	rs3914966
chr5	62798207	rs7708428
chr18	42891456	rs669924
chr12	31967313	rs10771892
chr22	37234748	rs1476002

chr6	6145459	rs1050783
chr11	32066554	rs11828792
chr13	49651694	rs1535468
chr3	2038877	rs6767146
chr8	10648603	rs7463228
chr18	36118973	rs1944320
chr9	24605823	rs1162608
chr17	32210771	rs2069145
chr12	58086924	rs7978954
chr5	172042829	rs6555988
chr21	25367698	rs2151
chr9	113624974	rs540599
chr9	91184097	rs10991808
chr4	54777238	rs2955174
chr10	128433760	rs4556457
chr2	154314345	rs7566164
chr20	37526060	rs12481150
chr17	52440377	rs4566219
chr13	48676905	rs9285169
chr7	94411687	rs42523
chr1	229053694	rs505557
chr18	40080773	rs749684
chr20	57015473	rs1299153
chr14	86826832	rs1642272
chr19	3421522	rs6510747
chr2	201343270	rs7577057
chr8	8555870	rs2294143
chr8	39728839	rs201995
chr6	69677201	rs6455338
chr12	95966695	rs10507068
chr8	57066755	rs16921553
chr9	75493953	rs1327369
chr4	52052572	rs2860055
chr4	100289558	rs4235450
chr10	8927839	rs1243411
chr12	18916925	rs16915276
chr11	30553091	rs16920925
chr3	143294361	rs6806500
chr13	85306989	rs1873288
chr17	44336113	rs7216307
chr21	39199320	rs1041439
chr12	20637424	rs7304868
chr2	232879311	rs709937

chr8	6839928	rs2977798
chr15	98334634	rs4965807
chr15	48720814	rs784417
chr11	86460412	rs618513
chr8	74568268	rs7015712
chr18	76720436	rs4393659
chr4	172855932	rs6833917
chr5	154532516	rs7703330
chr20	6220602	rs7261122
chr15	90154903	rs11853144
chr20	665209	rs6053798
chr3	1280141	rs17037117
chr13	74187563	rs7988935
chr1	111589599	rs12139261
chr11	105053519	rs1785883
chr10	90833199	rs12249377
chr11	120853309	rs6589848
chr3	133579276	rs6781238
chr13	43700594	rs393632
chr15	88355438	rs6496475
chr7	29205143	rs3812389
chr6	39261987	rs970392
chr9	32748367	rs12551778
chr10	101267649	rs2863114
chr6	35173084	rs942375
chr3	68316729	rs1491758
chr8	878266	rs11777864
chr2	78951004	rs11126689
chr17	79196371	rs2703549
chr6	25768796	rs1892250
chr5	74012518	rs194274
chr1	242538265	rs11804678
chr4	75004148	rs11098369
chr8	18021883	rs208021
chr17	13252736	rs11651762
chr6	144061157	rs2328542
chr6	96124872	rs11754559
chr5	74273438	rs569120
chr1	182137567	rs3795490
chr8	51530148	rs7003517
chr9	83609667	rs11792478
chr9	76607668	rs1952461
chr21	35061303	rs9636889

chr8	46266587	rs6651412
chr13	80031651	rs7995973
chr10	106249184	rs10509813
chr5	36482728	rs2468532
chr11	118939696	rs4938589
chr6	131805180	rs6935458
chr1	19000319	rs6699706
chr10	130829731	rs7893625
chr5	11487473	rs26150
chr4	148226671	rs3910044
chr16	85507778	rs8053670
chr2	53429466	rs1521943
chr10	52368653	rs1194683
chr18	69595900	rs10401068
chr3	114233314	rs2279828
chr17	1980517	rs7207686
chr7	157295131	rs757854
chr8	143580905	rs1809148
chr8	133523845	rs4736691
chr5	143502124	rs4912922
chr4	70196125	rs1383436
chr2	228465605	rs7592831
chr10	129876988	rs7076708
chr2	80517172	rs744957
chr8	115683666	rs17729317
chr7	156784620	rs2270264
chr11	71165389	rs7124728
chr13	62706741	rs4884429
chr5	56056943	rs10042443
chr3	102972148	rs17779020
chr2	180760937	rs4362518
chr18	48476387	rs4940383
chr5	66450712	rs13180603
chr9	9229566	rs17667884
chr1	211720313	rs7512315
chr2	30144358	rs1509578
chr6	31306916	rs3094691
chr19	29049890	rs6509207
chr15	91675087	rs7402600
chr7	26868977	rs10235680
chr3	116409558	rs4831139
chr6	52692829	rs2180323
chr1	27655356	rs12749246

chr17	47194314	rs858671
chr18	78677601	rs2941805
chr5	104991691	rs17500322
chr2	6215243	rs12464218
chr4	13219766	rs6838271
chr6	164262682	rs794108
chr14	22703080	rs4982652
chr6	41653533	rs2230088
chr12	94089430	rs1434704
chr10	43889310	rs12218783
chr17	73711840	rs2683173
chr5	73945145	rs283592
chr10	76389285	rs7909650
chr6	31379160	rs9501587
chr2	239123654	rs3791446
chr17	59433712	rs4968363
chr2	114985993	rs7603116
chr2	32270402	rs409188
chr6	84872588	rs9351013
chr7	74516081	rs2267824
chr7	10852858	rs6965606
chr3	166762771	rs9874962
chr10	65865461	rs16922054
chr2	237537599	rs10186871
chr3	123069512	rs9873177
chr20	20260931	rs996569
chr13	30800149	rs9315071
chr14	88028231	rs10151371
chr11	4959451	rs3850508
chr18	45418207	rs2612566
chr4	82739689	rs17354029
chr11	26993672	rs16916399
chr9	75498753	rs2778899
chr11	21587820	rs11602745
chr6	125133108	rs7748434
chr9	9264932	rs9299090
chr4	180582886	rs1422505
chr4	88172912	rs2622629
chr17	45271809	rs7213493
chr9	4844459	rs159432
chr3	180071246	rs9868787
chr10	5077352	rs7901781
chr6	41657691	rs2746183

chr6	4710828	rs1849564
chr10	80668365	rs7079135
chr12	75664031	rs10879956
chr6	31719231	rs1065356
chr20	100014	rs2196239
chr16	83862522	rs1123075
chr6	90792487	rs17523287
chr3	8428098	rs359006
chr13	90084010	rs2805713
chr3	158904996	rs17646246
chr15	61297886	rs4461014
chr12	31953515	rs17510714
chr22	47514518	rs1297370
chr6	13137533	rs10485363
chr4	152951291	rs6836054
chr1	15768821	rs2271545
chr6	29216883	rs3116837
chr4	162404259	rs17042479
chr15	98968644	rs2715423
chr6	31202737	rs9263870
chr3	21978302	rs2291818
chr1	70456677	rs1967189
chr10	22242274	rs12264839
chr2	32863477	rs6759807
chr3	140523972	rs349567
chr12	92133514	rs709219
chr13	91433032	rs12863915
chr7	145911936	rs4392813
chr10	95556283	rs4918948
chr12	66904238	rs10506515
chr11	21508216	rs12574030
chr13	27360569	rs9507881
chr16	4829254	rs7186281
chr6	143022633	rs200145
chr10	5400010	rs10795273
chr1	156135546	rs2485664
chr13	22285269	rs2038711
chr12	107163334	rs2374671
chr15	97879115	rs7497359
chr7	137305974	rs12666148
chr7	94301977	rs2106295
chr2	126586570	rs10204012
chr1	97449657	rs11165875

chr16	73678273	rs8063377
chr5	39435122	rs2887029
chr9	18656178	rs776780
chr1	77437296	rs10873941
chr13	77338047	rs7982454
chr6	8901291	rs9378578
chr2	37477793	rs957982
chr11	78008566	rs10899426
chr11	131431722	rs12790085
chr7	27313680	rs1005312
chr18	59475548	rs656750
chr6	25181219	rs9358839
chr1	97069794	rs290868
chr4	154074071	rs2404917
chr11	70349076	rs7109643
chr8	73557907	rs10087203
chr11	25842003	rs1869020
chr19	22584928	rs10420442
chr1	4801100	rs4654607
chr2	214016128	rs6749347
chr4	187577436	rs11734029
chr7	137321061	rs2242045
chr3	166566369	rs522409
chr14	95270110	rs1884535
chr20	8512003	rs6086477
chr2	180037368	rs12468587
chr9	113188426	rs10435864
chr9	4658449	rs1385452
chr20	51047504	rs6067620
chr3	133623144	rs10935070
chr1	168958376	rs10489370
chr8	82778844	rs7465344
chr13	86007732	rs4550343
chr9	107143332	rs956578
chr19	29073254	rs10422290
chr1	182268623	rs11802583
chr2	40064728	rs17025029
chr8	10456707	rs7017116
chr14	92264068	rs7156630
chr17	53210648	rs807101
chr6	29001153	rs9257453
chr3	46748907	rs7614762
chr10	81165287	rs12781461

chr17	45325639	rs4792814
chr8	73434627	rs4738353
chr17	57535839	rs12602441
chr11	17922329	rs2283240
chr3	192376955	rs12632797
chr2	158220317	rs6732439
chr4	183679448	rs4241779
chr3	30156401	rs7638300
chr12	43066689	rs1183790
chr7	156223532	rs1922086
chr8	1003948	rs13251306
chr10	45219921	rs1815630
chr18	23386675	rs11659634
chr2	29087182	rs7568662
chr3	131557875	rs12639456
chr12	129358045	rs10847836
chr15	92645641	rs3743360
chr1	27405147	rs11548323
chr15	96296956	rs4984430
chr1	206634569	rs3813965
chr16	74192322	rs807293
chr19	24163222	rs10500223
chr9	88276199	rs4877434
chr21	31729686	rs13052593
chr7	13236436	rs10488256
chr6	26608097	rs6919391
chr21	39712884	rs12233347
chr6	161546869	rs12526148
chr4	140114972	rs11100497
chr12	3216677	rs887363
chr4	167800352	rs13124475
chr9	9283557	rs10122609
chr12	27644972	rs7295096
chr8	39083415	rs4130393
chr4	90048414	rs11929845
chr10	4768369	rs1901633
chr1	245486539	rs12089406
chr19	8831041	rs2910368
chr3	6097693	rs17288023
chr13	62828217	rs1402552
chr8	57901056	rs2004902
chr12	25683814	rs10842596
chr3	140405774	rs10513112

chr17	9252737	rs6503190
chr8	134519905	rs4909456
chr6	32762309	rs2051549
chr20	53687590	rs6063977
chr5	169025472	rs6890327
chr17	41647748	rs6503650
chr12	89381409	rs12318428
chr8	100588738	rs2507780
chr6	137219938	rs1327474
chr9	69104702	rs4744826
chr2	228878293	rs7578485
chr10	27404376	rs1334893
chr1	94115569	rs10782976
chr21	42142587	rs7280633
chr10	16096490	rs11253779
chr13	44897554	rs9534002
chr2	52325885	rs12468211
chr1	37726624	rs6686248
chr2	67285986	rs13385154
chr7	145144438	rs10487524
chr1	21599110	rs10917023
chr20	10255278	rs3025866
chr16	18933753	rs9934555
chr6	128630162	rs17056140
chr1	54643305	rs10888858
chr10	30726604	rs4080824
chr3	165053083	rs9883965
chr20	45268950	rs8113868
chr14	105532207	rs4074453
chr2	100061736	rs11695782
chr15	101427596	rs4965384
chr7	51884689	rs7801857
chr8	141611385	rs1004380
chr6	158708519	rs9456341
chr3	152755987	rs1381094
chr10	13015323	rs11258127
chr5	137017151	rs12188192
chr18	59435094	rs9954602
chr15	62929031	rs4774458
chr4	172871712	rs17058978
chr7	48147035	rs10081256
chr5	126342662	rs6595693
chr14	66496531	rs3737160

chr5	125867587	rs389333
chr5	121908825	rs12652819
chr22	31155174	rs4820946
chr7	48527548	rs4072502
chr1	94256104	rs12750249
chr17	1961455	rs9646401
chr15	62378487	rs289094
chr1	196150180	rs12727479
chr9	113360046	rs1077636
chr4	107867524	rs11940819
chr6	143997861	rs4552753
chr5	143657295	rs1427864
chr4	82743054	rs7679085
chr21	41887220	rs2282109
chr3	176535281	rs7636407
chr7	136665993	rs10235715
chr4	83715043	rs17007216
chr11	134612572	rs1944866
chr3	66746608	rs9845175
chr15	62173190	rs17205603
chr7	5578606	rs2693946
chr6	14407873	rs9476556
chr6	67020800	rs10944945
chr11	60965272	rs548363
chr20	57086493	rs6069979
chr12	125442792	rs988535
chr5	62737212	rs17471355
chr11	86781798	rs744293
chr22	47168657	rs5767557
chr13	88365392	rs1074201
chr9	135984004	rs2152174
chr15	71827228	rs10518970
chr9	130908179	rs4740383
chr13	28472190	rs615529
chr17	31677115	rs425083
chr13	106568505	rs3759453
chr6	105627372	rs11152903
chr5	144378814	rs10515542
chr13	38747305	rs11147730
chr3	48621576	rs9834639
chr10	105522573	rs11192469
chr21	30463521	rs8134027
chr7	14176817	rs17167945

chr8	125317088	rs13260832
chr11	69025738	rs11228444
chr9	7829623	rs9299057
chr3	171803823	rs13075194
chr16	24309866	rs2238510
chr4	106378884	rs940618
chr9	4543307	rs2026828
chr9	124835397	rs4838224
chr8	19473424	rs10503653
chr17	49278661	rs2898857
chr21	24236321	rs2828925
chr12	41403584	rs12305552
chr10	2093616	rs7101173
chr17	7376950	rs4796412
chr8	100585058	rs16898600
chr2	154194291	rs6435067
chr2	137333487	rs6740175
chr2	114461398	rs4849337
chr18	67997725	rs3862732
chr6	89945903	rs9351230
chr11	7156596	rs3105717
chr4	164370625	rs2102296
chr11	45585433	rs1488682
chr11	4180411	rs2044138
chr10	6239257	rs11599520
chr11	116275904	rs876411
chr9	117785004	rs12352822
chr16	84870122	rs4782677
chr16	83857280	rs450204
chr11	11423943	rs901545
chr19	53539198	rs3746305
chr4	178306726	rs1567474
chr10	125711864	rs7909932
chr6	31903079	rs9267665
chr11	108632945	rs10890873
chr11	26151223	rs4072100
chr9	127127444	rs1281156
chr22	22032219	rs9610900
chr14	64799363	rs1268663
chr10	19865320	rs10508599
chr1	17723377	rs11581535
chr10	116107992	rs2694798
chr5	168430999	rs729149

chr13	102147884	rs17632458
chr10	25855536	rs10508707
chr13	24934511	rs2084756
chr5	17199965	rs11952802
chr18	4070978	rs9789161
chr18	65484712	rs8091665
chr17	45138914	rs4986172
chr4	11305279	rs17468208
chr8	122285462	rs4871240
chr7	12102651	rs6963473
chr16	83011060	rs12925746
chr11	132833690	rs4937729
chr9	29661361	rs10511831
chr3	186185593	rs741483
chr8	19899302	rs10102717
chr10	54355097	rs11594789
chr11	21511321	rs4074586
chr10	26415585	rs945296
chr2	126891370	rs11899448
chr13	48748046	rs9591208
chr2	37245661	rs2110965
chr12	28871601	rs7294633
chr9	14562632	rs1556029
chr2	79232860	rs1434194
chr10	3820322	rs11252121
chr20	8181631	rs6055603
chr3	65316647	rs1479958
chr11	32555152	rs1872706
chr6	20901080	rs6456382
chr1	81206156	rs17106149
chr3	126254742	rs10934761
chr11	125118620	rs608393
chr3	179310025	rs6794192
chr8	8690132	rs17684514
chr2	19524342	rs13407212
chr15	62109283	rs2083013
chr17	44973977	rs9908256
chr13	82056760	rs9531286
chr21	19917101	rs2825891
chr19	29185867	rs10518258
chr4	62001735	rs13115125
chr5	135753963	rs7380956
chr15	47394393	rs11070582

chr9	82735797	rs4877702
chr1	21553031	rs3753782
chr20	48159082	rs827944
chr7	138269674	rs6963140
chr21	46004432	rs9254
chr1	77438131	rs7545638
chr10	116765705	rs1615201
chr3	191571662	rs4074485
chr1	205349855	rs913722
chr20	53634337	rs204767
chr5	8061839	rs9313221
chr15	40170579	rs1801389
chr7	127926425	rs12706823
chr2	181583683	rs12105941
chr22	26597742	rs5761618
chr7	12244161	rs1990622
chr5	36541200	rs9292633
chr9	69388120	rs867366
chr7	86250461	rs1860784
chr2	104098868	rs11896903
chr13	82031535	rs12184745
chr3	194749262	rs9881429
chr6	6009615	rs3763180
chr11	100170688	rs2044983
chr17	8908361	rs726679
chr5	148124257	rs1422993
chr6	43831165	rs9381257
chr21	39888335	rs17753847
chr11	84842764	rs952201
chr1	189053479	rs7546105
chr3	105003955	rs6797921
chr16	4354619	rs10974
chr12	129517949	rs12321897
chr7	155967896	rs6459975
chr4	72755212	rs7683184
chr5	23371054	rs10058259
chr3	14309159	rs4684204
chr8	138060575	rs10101858
chr5	20443591	rs6877123
chr4	139178614	rs1471928
chr9	124262893	rs2282087
chr1	202705069	rs4072661
chr9	953465	rs10759073

chr1	60334990	rs390331
chr4	61238394	rs1827770
chr2	14998162	rs6432496
chr8	10258211	rs4504638
chr2	41878491	rs10204805
chr2	103993282	rs935070
chr7	19940344	rs6947148
chr2	10472504	rs818167
chr21	33355024	rs1041868
chr5	145115832	rs12109366
chr12	27602525	rs12319361
chr9	80336313	rs7852248
chr6	115267315	rs996139
chr6	30106070	rs2844795
chr2	223247821	rs4674771
chr16	7176672	rs12935311
chr8	12989398	rs6531017
chr14	69243212	rs10148349
chr16	82579883	rs17245633
chr10	35600599	rs660301
chr10	6721787	rs1327690
chr7	29304852	rs3812333
chr7	29651597	rs1728539
chr18	24632226	rs339858
chr6	99991133	rs2001456
chr16	85524373	rs7500151
chr20	19940543	rs199549
chr1	2308567	rs2173049
chr10	9808336	rs11256348
chr4	37857269	rs6855439
chr10	61246113	rs1906470
chr10	30629495	rs10826831
chr13	20456753	rs3936015
chr14	63768838	rs7157785
chr18	69583277	rs4381690
chr9	126887077	rs3120029
chr20	19949783	rs11699866
chr12	129409961	rs1976829
chr4	181292756	rs4861940
chr10	24010425	rs12146261
chr2	158235629	rs12994903
chr10	118604432	rs4751667
chr2	176517861	rs2969344

chr3	24723009	rs9310754
chr2	133819684	rs7603317
chr7	38024480	rs6462801
chr7	33368282	rs3823763
chr3	174684796	rs12493995
chr16	7102864	rs11640267
chr1	67605699	rs787499
chr2	230682460	rs17762352
chr17	80394251	rs8359
chr7	79956135	rs10229206
chr7	83931990	rs701307
chr11	13094185	rs1899302
chr14	58780541	rs17095093
chr12	58833772	rs17121530
chr9	114270168	rs2636864
chr4	168421369	rs2014622
chr18	42797879	rs1943177
chr11	56310347	rs11227836
chr11	100022550	rs698698
chr13	28489832	rs600640
chr8	104629394	rs4236775
chr4	73426690	rs16849364
chr14	94470627	rs8014978
chr12	3115820	rs10774120
chr6	91685330	rs575844
chr1	11233902	rs11121704
chr20	10262603	rs362563
chr1	208133135	rs17187133
chr4	31211185	rs1875050
chr16	67648677	rs9972635
chr4	172230668	rs9312518
chr8	82662197	rs7833831
chr20	372441	rs742605
chr18	68778039	rs677592
chr8	15779883	rs1362867
chr13	46176746	rs17601960
chr18	6306122	rs1946753
chr10	9665689	rs10905601
chr4	134529832	rs10005804
chr3	143425726	rs1554672
chr9	3828745	rs17742098
chr4	21115590	rs16870429
chr1	168608236	rs7517738

chr17	44980217	rs11871217
chr18	45682394	rs3745009
chr9	90342047	rs7048728
chr17	9658226	rs429068
chr22	30274004	rs6006427
chr9	79957293	rs962883
chr10	1663053	rs1909428
chr11	133907493	rs1106561
chr14	104373479	rs4074324
chr6	8357004	rs1572852
chr7	33919627	rs1427483
chr11	70496312	rs573795
chr10	87578876	rs9664222
chr4	34560067	rs1480990
chr6	31090563	rs3130544
chr9	115223607	rs11794549
chr1	236598478	rs2298099
chr4	74150033	rs7660423
chr17	81091850	rs4969384
chr3	167070719	rs6773278
chr6	147894552	rs4385334
chr6	29572427	rs1003581
chr9	113231643	rs10513190
chr7	129529642	rs4574773
chr9	7379168	rs7871958
chr7	145979789	rs7777578
chr9	108749712	rs10816710
chr2	3078332	rs11127398
chr19	46256322	rs10406931
chr1	4812996	rs10915612
chr8	86812523	rs7011051
chr3	133756878	rs1799852
chr19	23580362	rs9305005
chr6	32832447	rs2228397
chr12	51262867	rs4761815
chr7	127656948	rs806172
chr19	19678719	rs2304130
chr5	7876175	rs326121
chr10	5244199	rs9423393
chr2	30692520	rs530076
chr7	155905561	rs6459961
chr15	86975681	rs2082538
chr14	80310393	rs178954

chr14	42866381	rs17541557
chr2	239725708	rs6734016
chr3	167631050	rs7627289
chr1	111598795	rs12123811
chr7	25036493	rs10276962
chr8	124688684	rs2382993
chr14	24434758	rs12891613
chr6	4514241	rs6926616
chr12	7538538	rs7136716
chr2	157806765	rs17798043
chr16	4634447	rs1659506
chr4	63468092	rs10021833
chr3	88413455	rs1458005
chr5	137086669	rs2158409
chr11	100143170	rs2012260
chr12	12930203	rs1291352
chr5	16588931	rs2451852
chr9	115979439	rs1349323
chr18	63017447	rs12958400
chr12	26619892	rs11611622
chr6	160421693	rs4708867
chr14	32382339	rs942028
chr5	169037736	rs1059160
chr2	21460413	rs6749957
chr6	5908931	rs7775306
chr5	143645062	rs12657648
chr18	76704130	rs9953092
chr7	37923216	rs6964434
chr4	77271165	rs10491463
chr11	18143140	rs2445162
chr15	54504394	rs1520410
chr8	71176937	rs4103014
chr3	1150935	rs2036236
chr4	105089276	rs12509636
chr7	38539779	rs12538952
chr17	46915752	rs1662596
chr3	166977837	rs1608323
chr7	12160434	rs6952272
chr2	169415592	rs10168193
chr20	39671934	rs11698332
chr3	163855119	rs6774925
chr9	4347422	rs7034125
chr16	89842993	rs12599531

chr10	91131307	rs7067899
chr3	187132898	rs2270354
chr4	87811759	rs1054628
chr12	125748335	rs11058317
chr7	2208837	rs1612548
chr19	30180286	rs2866721
chr1	246799717	rs3935608
chr19	23494599	rs17000180
chr6	159527839	rs395538
chr7	45644205	rs9639925
chr7	3553933	rs13221445
chr9	24617371	rs1184457
chr15	54506655	rs537389
chr10	95530235	rs11188346
chr6	133202650	rs477094
chr16	73743658	rs367823
chr3	52036939	rs9848727
chr1	167669014	rs1229363
chr17	39209446	rs486512
chr6	151599637	rs1340874
chr9	18332002	rs10810957
chr13	30429481	rs1141362
chr15	90839685	rs2532101
chr4	37479065	rs1382979
chr14	68738840	rs6573856
chr13	41180534	rs1999507
chr7	129773348	rs4626538
chr12	101677741	rs10778137
chr4	77348973	rs11936421
chr5	32596740	rs11952155
chr12	11597203	rs11054362
chr8	139120605	rs11166878
chr6	31084350	rs2535315
chr4	7156740	rs10034147
chr2	222132385	rs2894450
chr6	123896568	rs11154196
chr18	75303428	rs4891260
chr2	155627317	rs843236
chr2	129375792	rs2030664
chr15	66804320	rs7183242
chr4	35247225	rs7686175
chr10	54259434	rs2043995
chr9	4352193	rs11789687

chr12	103049487	rs11111423
chr9	69812567	rs4744942
chr5	172549963	rs7442753
chr11	100052909	rs11222552
chr10	3111696	rs10508245
chr9	77000825	rs4745607
chr2	154242633	rs9288324
chr3	87358096	rs9861650
chr20	56981100	rs1276466
chr15	95751875	rs8025900
chr1	182135054	rs3845452
chr20	3846662	rs4815617
chr14	100732404	rs2273608
chr9	4357693	rs12005536
chr10	124647128	rs12411439
chr7	29332423	rs39132
chr5	31713339	rs7725382
chr4	114492457	rs7698391
chr3	28078149	rs4680899
chr2	67413146	rs13424211
chr9	2024076	rs6475412
chr18	38541190	rs1426685
chr18	65506098	rs12965811
chr12	43780963	rs4251580
chr15	71253178	rs10518937
chr15	53552900	rs1906426
chr8	134504037	rs4366044
chr3	192142746	rs1460924
chr11	103695668	rs7924591
chr6	13650739	rs6458997
chr4	7182257	rs7692512
chr11	113278920	rs668204
chr1	61663460	rs6587943
chr10	79955982	rs2146192
chr11	86793314	rs12799567
chr18	4545834	rs7238906
chr10	95630809	rs12773566
chr6	31353434	rs2596501
chr7	144783456	rs1635357
chr4	164638911	rs9790550
chr19	4635390	rs17363184
chr13	26992117	rs9581798
chr7	105972360	rs2727758

chr4	37263209	rs3910363
chr5	36520411	rs4869483
chr20	39129026	rs2868502
chr8	134529841	rs1550582
chr16	83087005	rs6565116
chr19	57153977	rs10416425
chr2	104072986	rs2376064
chr1	26024891	rs17163470
chr8	8801528	rs3827811
chr10	8978271	rs827628
chr19	16758152	rs773845
chr10	3657428	rs10904068
chr6	45627709	rs12663992
chr3	188908099	rs6797432
chr16	4941053	rs2908659
chr18	50128582	rs17716604
chr4	164384737	rs7658861
chr4	16493328	rs1501141
chr2	855459	rs11682609
chr5	174120380	rs11739519
chr15	53493736	rs952993
chr7	137431539	rs918892
chr22	45506239	rs13433666
chr5	110403370	rs11241068
chr12	76043993	rs2043369
chr6	92449257	rs878519
chr14	64921525	rs10138506
chr8	1183355	rs12334702
chr18	59546924	rs3133202
chr4	129626198	rs10001198
chr3	186964439	rs11712248
chr8	22917906	rs2466215
chr13	39623967	rs2324452
chr9	28804100	rs824230
chr9	106965791	rs6477551
chr6	161495448	rs9458289
chr12	7570221	rs1419974
chr1	5874176	rs868162
chr3	70121641	rs13084430
chr11	34179738	rs910257
chr8	136767016	rs2610088
chr5	161263574	rs173565
chr21	26427688	rs11702375

chr12	97393283	rs1861370
chr17	40563547	rs3136685
chr6	118211386	rs205915
chr14	91678893	rs6575209
chr11	12504378	rs11547363
chr7	12218340	rs3887296
chr17	35193098	rs17547201
chr3	30166176	rs1433986
chr6	133219057	rs9402490
chr10	117994452	rs10886189
chr2	18480716	rs1489689
chr12	31204168	rs10492354
chr4	37392774	rs17421722
chr4	185290827	rs1288542
chr5	133099880	rs4367292
chr11	104837005	rs642603
chr1	63082002	rs17124579
chr3	5295442	rs1391961
chr4	132325751	rs10518610
chr10	24005138	rs2027238
chr1	160549450	rs3766387
chr2	239118750	rs3791428
chr11	132816089	rs12285031
chr6	27021213	rs7741445
chr15	35774872	rs1846135
chr6	730114	rs7758338
chr20	15082304	rs383310
chr4	123670948	rs2589840
chr9	991114	rs279877
chr3	20747089	rs964911
chr17	61211535	rs1860373
chr14	104341375	rs8016091
chr3	10294102	rs2619507
chr15	90808700	rs389480
chr14	78003171	rs1955568
chr8	69669870	rs16936267
chr21	45439803	rs9980531
chr7	29341533	rs986226
chr12	107264444	rs12322483
chr4	164443105	rs12152616
chr2	55667112	rs782637
chr6	8324224	rs2792613
chr20	53907007	rs6022842

chr11	119027609	rs741811
chr10	7655110	rs17142892
chr1	98130297	rs1487302
chr3	62138804	rs2366684
chr1	111588002	rs2800880
chr10	30109862	rs2505115
chr14	31083414	rs10483353
chr5	126627359	rs11958842
chr10	88830989	rs10736363
chr15	52695991	rs7161887
chr9	110834829	rs2821153
chr11	64329761	rs694739
chr10	76395529	rs749127
chr4	76806352	rs13124337
chr10	76362971	rs12766217
chr3	30807569	rs1393751
chr22	44203651	rs139171
chr9	8468780	rs13286656
chr1	40500511	rs7512061
chr1	55342470	rs207145
chr15	63142997	rs12899931
chr11	45395539	rs2863706
chr8	125260404	rs10283134
chr6	41659325	rs1474761
chr2	178293219	rs836882
chr7	135286658	rs11762667
chr20	39661823	rs1655353
chr4	72508307	rs788930
chr11	45463950	rs901901
chr4	61214497	rs6845545
chr9	101770154	rs16920725
chr18	63669134	rs12960185
chr3	165550120	rs11917072
chr10	101269303	rs10883603
chr8	101050430	rs2122922
chr8	73757174	rs12545117
chr19	52531345	rs781129
chr10	6234611	rs1064891
chr6	79723374	rs9448767
chr18	75104476	rs894575
chr6	40019576	rs4714285
chr2	240228496	rs7420256
chr10	60126288	rs12761506

chr3	24724765	rs6794687
chr14	70882372	rs8023177
chr11	130405451	rs2242312
chr13	49409673	rs12429253
chr8	47670402	rs7842068
chr12	129369883	rs900263
chr8	125105917	rs4871570
chr15	97151390	rs12912621
chr3	62045175	rs9311834
chr13	34617439	rs2026064
chr18	48461304	rs1877412
chr1	80124273	rs12119851
chr10	66821589	rs12763505
chr1	168991048	rs1322487
chr8	118307229	rs16890815
chr4	11502237	rs924000
chr7	104629171	rs759532
chr10	4771019	rs1901632
chr5	16186665	rs342561
chr9	83717928	rs11793897
chr11	56194765	rs1384061
chr19	4804512	rs8102626
chr13	46871568	rs9316235
chr5	31736475	rs375183
chr8	73626463	rs10086512
